# Synthesis of Novel Amino Acid–Fipronil Conjugates and Study on Their Phloem Loading Mechanism

**DOI:** 10.3390/molecules23040778

**Published:** 2018-03-28

**Authors:** Qingqing Sheng, Xinxin Liu, Yun Xie, Fei Lin, Zhixiang Zhang, Chen Zhao, Hanhong Xu

**Affiliations:** 1 State Key Laboratory for Conservation and Utilization of Subtropical Agro-Bioresources, South China Agricultural University, Guangzhou 510642, China; shengqing277@163.com (Q.S.); 15913194557@163.com (X.L.); xieyun91@foxmail.com (Y.X.); resistanc@scau.edu.cn (F.L.); zdsys@scau.edu.cn (Z.Z.); 2 Key Laboratory of Natural Pesticide and Chemical Biology, Ministry of Education, South China Agricultural University, Guangzhou 510642, China

**Keywords:** phloem-mobile pesticides, fipronil, passive diffusion, amino acid transporter

## Abstract

To develop a new pesticide with phloem mobility, a series of new amino acid–fipronil conjugates were designed and synthesized based on derivatization at the 3-position of the pyrazole ring of fipronil. Experiments using a *Ricinus communis* seedling system showed that all tested conjugates were phloem mobile except for the isoleucine–fipronil conjugate, and that the serine–fipronil conjugate (**4g**) exhibited the highest concentration in phloem sap (52.00 ± 5.80 μM). According to prediction with log *Cf* values and uptake experiments with *Xenopus* oocytes, the phloem loading process of conjugate **4g** involved both passive diffusion and an active carrier system (*RcANT15*). In particular, compared with for a previously reported glycinergic–fipronil conjugate (GlyF), passive diffusion played a more important role for conjugate **4g** in the enhancement of phloem mobility. This study suggests that associating a nutrient at a different position of an existing pesticide structure could still be effective in obtaining phloem-mobile derivatives, but the distinct physicochemical properties of resultant conjugates may lead to different phloem loading mechanisms.

## 1. Introduction

**Figure 1:**
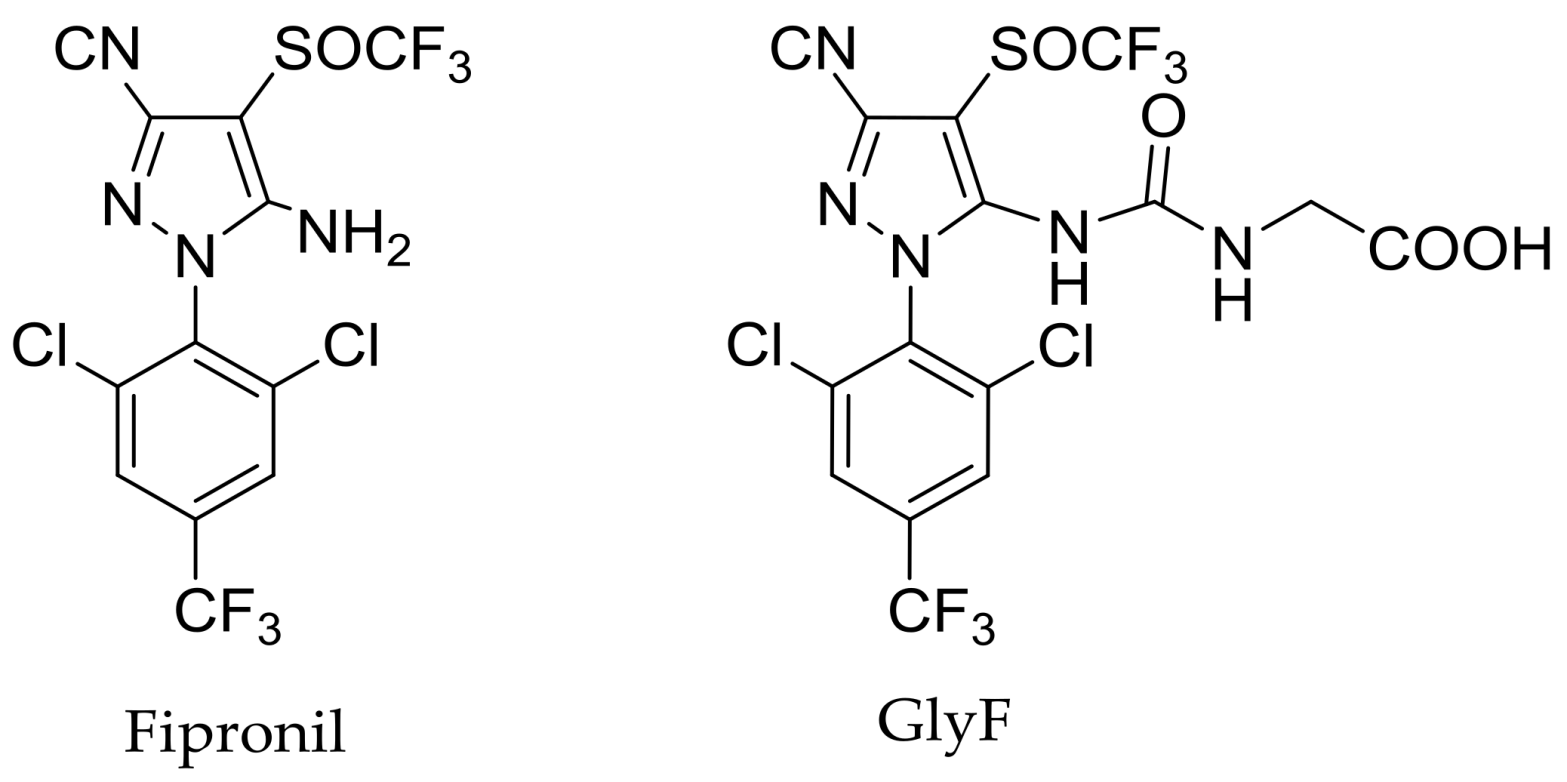
Chemical structure of fipronil and the glycinergic–fipronil conjugate (GlyF) [15].

Phloem mobility [1,2] of a pesticide is an attribute that contributes positively to its efficacy against piercing and sucking insects, especially pests hidden in nonexposed plant parts such as growing tips and roots [3]. Many attempts have been made to develop phloem-mobile pesticides via structural modifications on existing pesticide structures [4,5,6,7,8,9,10,11,12,13]. For example, in our previous work, the non-phloem-mobile pesticide fipronil was conjugated with endogenous nutrients including monosaccharides and amino acids to obtain phloem-mobile derivatives, such as glucose–fipronil conjugate (GTF) [14] and glycinergic–fipronil conjugate (GlyF) (Figure 1) [15]. The uptake process of GlyF was demonstrated to involve an active carrier system and to induce up-regulated expression of four amino acid transporter (AAT) genes (*RcLHT6*, *RcANT15*, *RcProT2*, and *RcCAT2*). 

Although proven to be feasible in conferring phloem mobility, the current derivatizations on fipronil were limited to one specific position of the parent structure: the amino group at the 5-position of pyrazole. According to the assumption that the additional phloem mobility was led by the affinity between the amino acid fragment on conjugates and amino acid transporters in plants, derivatization at other positions on the parent structure with the same nutrient substituent could also lead to phloem-mobile compounds. To support this hypothesis, in this study, a new series of amino acid–fipronil conjugates based on derivatization at the 3-position of the pyrazole ring of fipronil (**4a**–**l**) was designed and synthesized, and their phloem mobility was measured in *R. communis* seedlings. Additionally, nutrient–pesticide conjugates with different conjugating sites may present different physicochemical properties, which could also affect their ability to permeate the plasma membrane [16,17,18,19]. Thus, experiments including prediction with log *Cf* values and uptake experiments with *Xenopus* oocytes were conducted to compare the new and previous conjugates, and the results could help provide a deeper understanding of the phloem loading mechanism of similar xenobiotics. 

## 2. Results and Discussion

### 2.1. Synthesis and Characterizations

**Scheme 1:**
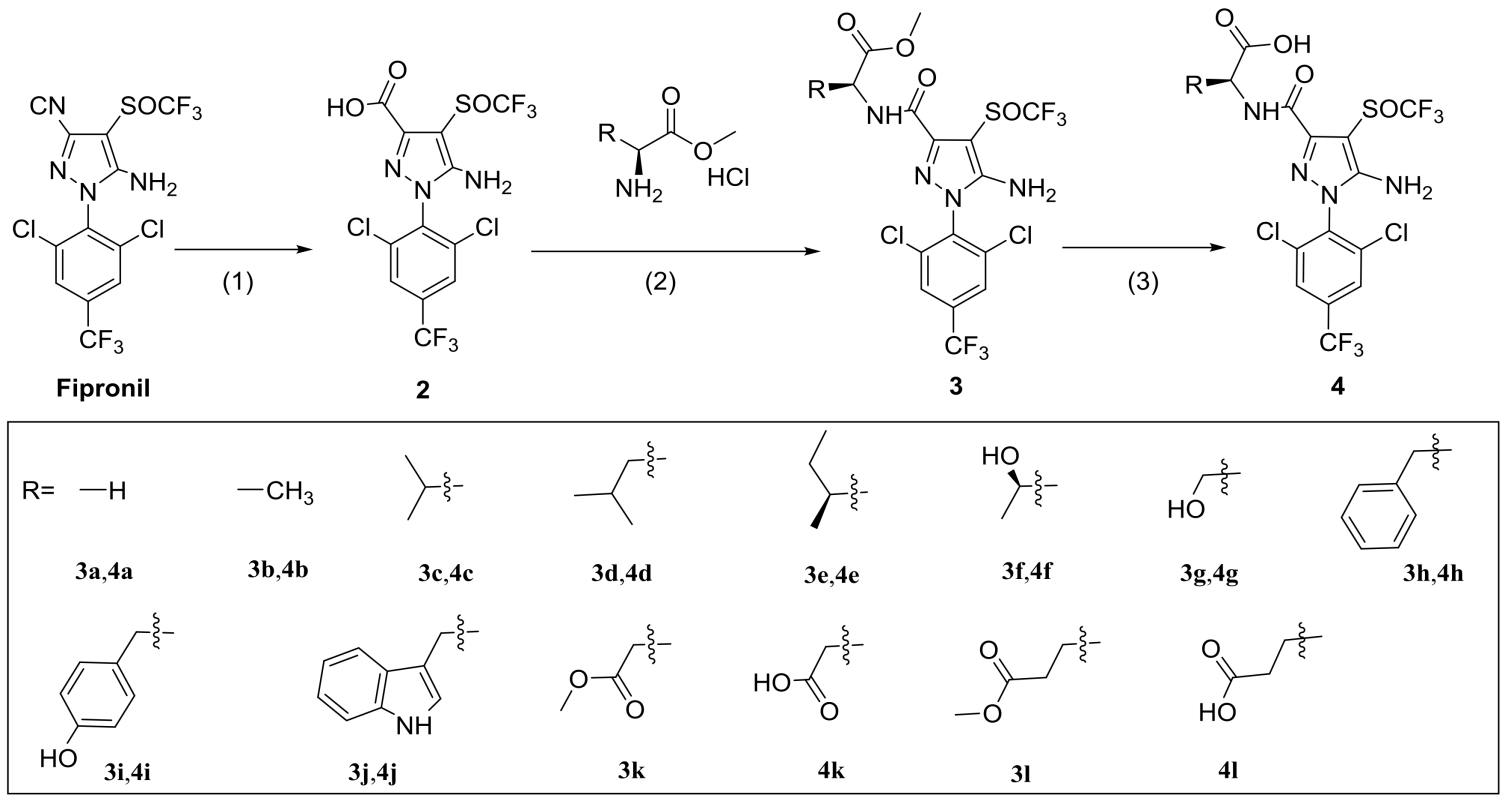
Synthetic routes and chemical structures of compounds **2**–**4**. Reagents and conditions: (1) 98% concentrated sulfuric acid, acetic acid, H_2_O, 120 °C, 2 h; (2) *N*-methylmorpholine, EDC∙HCl, DMAP, anhydrous dichloromethane, room temperature (r.t.); (3) lithium hydroxide, THF/H_2_O (*v*/*v* = 2:1), r.t.

The new conjugates (**4a**–**l**) were synthesized by combining fipronil and natural amino acids at the 3-position of the pyrazole ring of fipronil [20,21,22] following a three-step synthetic route. As shown in Scheme 1, the cyano group on the pyrazole ring was first hydrolyzed into a reactive carboxyl group [23], which enables easy chemical modification. Followed by condensation reactions with amino acid esters in the presence of EDC∙HCl and DMAP [24,25], 12 amino acid ester–fipronil conjugates (**3a**–**l**) were obtained. Hydrolysis of the methyl ester group in **3a**–**l** with lithium hydroxide [10,26] provided the new amino acid–fipronil conjugates **4a**–**l** in good yields. All structures were confirmed via ^1^H, ^13^C NMR spectroscopy and ESI mass spectrometry.

### 2.2. Phloem Mobility in R. communis Seedlings

Phloem mobility of amino acid–fipronil conjugates **4a**–**l** was measured in *R. communis* seedlings, which are an ideal biological model to evaluate the phloem mobility of xenobiotics because of their thin and highly permeable cuticles [16,27]. For each measurement, *R. communis* seedlings were incubated with 100 μM of the compound to be tested. Phloem sap was then collected and analyzed using High-Performance Liquid Chromatography (HPLC) [9]. 

As shown in Table 1, all conjugates could be detected in phloem sap except for conjugate **4e** (isoleucine–fipronil). This verified our hypothesis that conjugation of an amino acid fragment at a different position on the pyrazole ring of fipronil is feasible for acquiring phloem mobility for the non-phloem-mobile parent compound. In general, the phloem mobility of compounds that have a small substituent at the *α*-position of the amino acid fragment was better than that of the ones containing branched alkyl groups, aromatic rings, or heterocycles. Among them, conjugate **4g** (serine–fipronil) showed the highest phloem mobility, with the concentration in phloem sap being 52.00 ± 5.80 μM.

It is noteworthy that compared with previously reported GlyF [15], nine of the conjugates (**4a**, **4b**, **4c**, **4d**, **4f**, **4g**, **4i**, **4k**, and **4l**) exhibited better phloem mobility. In particular, the concentration in phloem sap of conjugate **4g** was five times as high as that of GlyF. As all compounds have a free carboxylic acid function, it was possible that the ion trap mechanism was involved in the phloem transport of these conjugates. However, according to the prediction with ACD/Labs version 14.0 software (classical method), the net charges of all compounds were at −1 between pH 5.6 and 8.0. Therefore, these compounds do not match the characteristics of the ion trap mechanism [27]. Considering that passive diffusion and active transportation are the two major mechanisms involved in phloem loading of xenobiotics, two factors may have caused the observed enhancement in phloem mobility: (1) physicochemical properties that facilitate passive diffusion; and (2) higher affinity to associate with amino acid carriers which promote active transportation [28,29]. Further experiments were then conducted to analyze the respective significance of the two aspects.

### 2.3. Prediction of Phloem Mobility Using Log Cf Values

Predictions on phloem mobility of amino acid–fipronil conjugates were then performed based on physicochemical properties (log K*_o/w_* and pK_a_) of compounds using log *Cf* values [1]. For most of the tested xenobiotics, the experimental data fitted well with theoretical predictions. However, the phloem mobility of many carrier-mediated xenobiotics would not fit in the prediction of log *Cf* values, because it lacks full consideration of biological parameters concerning penetration across the leaf cuticle, metabolism, cell compartmentation, and, in particular, the active transportation mechanism. As a result, the predicted results mainly represent the ability of xenobiotics to enter into phloem through passive diffusion [16,27]. Thus, analysis of the predicted phloem mobility may help us to reveal if the physical properties of new conjugates enabled better passive diffusion compared with those of GlyF.

As shown in Table 2 [1], conjugates **4a**–**l** were predicted to have different phloem mobility. The log *Cf* values of seven conjugates (**4a**, **4b**, **4c**, **4f**, **4g**, **4k**, and **4l**) ranged from −4 to 1, indicating that these conjugates had moderate phloem mobility, and the phloem uptake of these compounds involved the process of passive diffusion. The log *Cf* values of five conjugates (**4d**, **4e**, **4h**, **4i**, and **4j**) were below −4, suggesting that these xenobiotics have no phloem mobility. Considering that all conjugates except **4e** were actually phloem mobile in *R. communis* seedlings, the uptake of **4d**, **4h**, **4i**, and **4j** was carrier meditated. Notably, the conjugates with log *Cf* values from −4 to 1 in general showed higher phloem mobility than the ones with log *Cf* values below −4. In particular, compared with GlyF, the uptake process of which was proved to be carrier meditated [15], all conjugates with log *Cf* values ranging from −4 to 1 presented higher phloem mobility. This demonstrated that the ability to penetrate the plasma membrane through passive diffusion dominated the overall phloem loading behavior for the new conjugates with high phloem mobility, and was the key factor that caused the enhanced uptake compared with GlyF (Table 2). Uptake experiments with *Xenopus* oocytes were then conducted to provide experimental evidence for this deduction.

### 2.4. Uptake Experiments with Xenopus Oocytes

In our former research, it has been demonstrated that *RcANT15* has the function of transporting GlyF [30]. Considering the structural similarity between the new series of conjugates and GlyF (both combinations of amino acid and fipronil), it is supposed that the same active-carrier-mediated mechanism may still effectively contribute to the phloem loading of the new series of conjugates. Thus, a cellular-level uptake experiment with *Xenopus* oocytes under the mediation of *RcANT15* was conducted with conjugate **4g**, which presents the highest phloem mobility in *R. communis* seedlings. Conjugate **4a**, which contains the same glycine fragment as GlyF, was also tested under the same experimental condition as a comparison. For each measurement, cRNA of *RcANT15* was injected into *Xenopus* oocytes, and the oocytes were treated with 0.1 mM of the conjugates being tested for one hour before analysis for the amount of conjugate absorbed. *Xenopus* oocytes injected with the same volume of Nuclease-Free water were used as a control [31]. Although the contribution of other carriers to the uptake process was not excluded in this experiment, the result was able to show a general scope for the uptake process.

**Figure 2:**
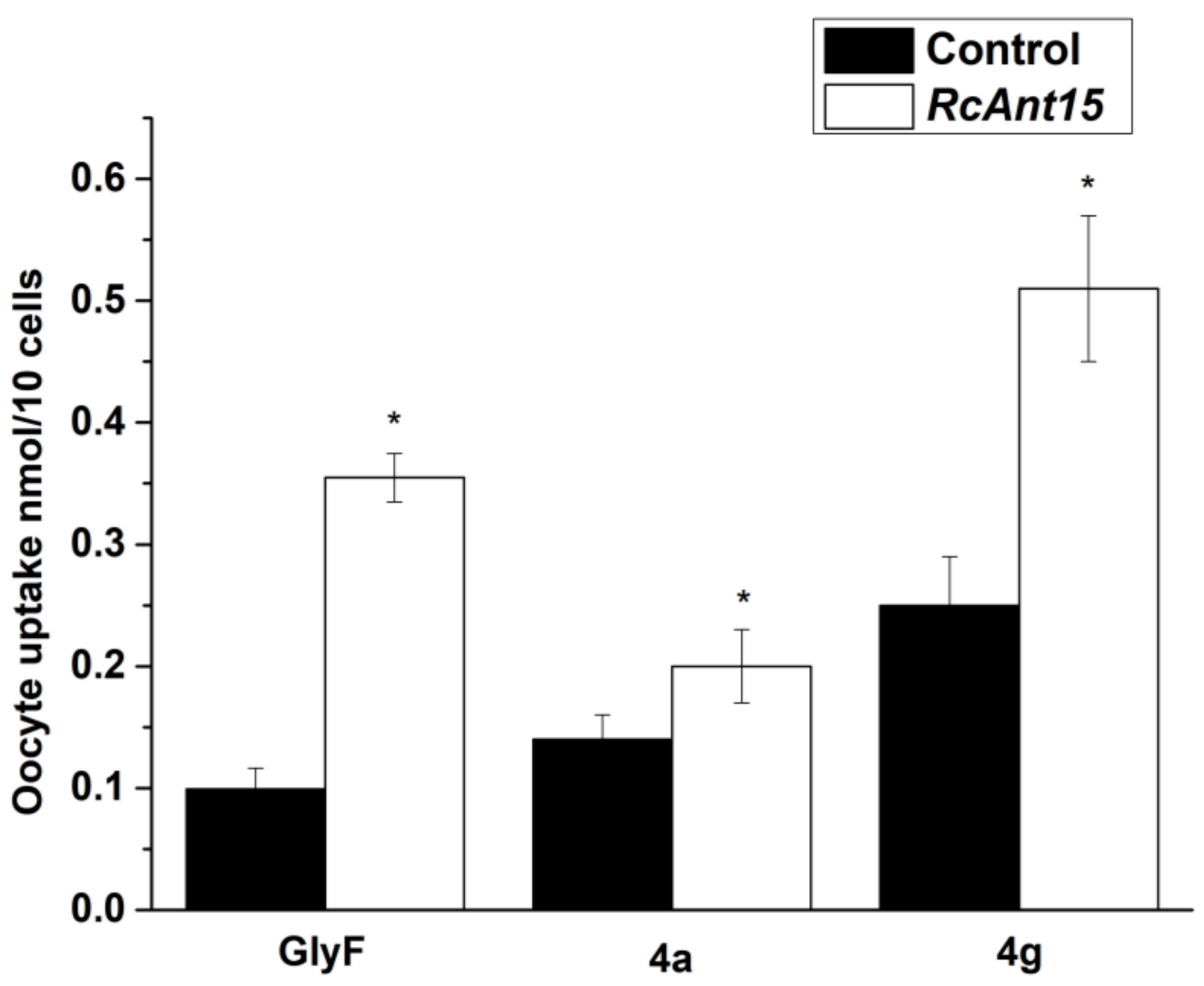
Uptake of GlyF, **4a**, and **4g** by *Xenopus* oocytes after treatment with 100 μM of conjugates at pH 5.6 for one hour. Each oocyte was injected with 18.4 nL of amino acid transporter *RcANT15* cRNA (white column), and oocytes injected with the same volume of Nuclease-Free water were used as a control (black column). Data (mean ± SE, *n* = 4) columns marked with * are significantly different from the control set, as determined by Mann–Whitney U test (*p* < 0.05). Data on GlyF were from Xie et al. [30].

The transport activity for endogenous substances [32], especially for amino acids [33], was weak in the plasma membrane of the *Xenopus* oocytes system. Thus, the ability of *Xenopus* oocytes without *RcANT15* cRNA expression to transport xenobiotics could be ignored [34,35,36], and the uptake of the candidate compounds in the control group was mainly driven by passive diffusion. As shown in Figure 2, the uptake levels of conjugates **4g** and **4a** in oocytes injected without *RcANT15* cRNA (0.25 and 0.15 nmol/10 cells, respectively) were both higher than that of GlyF (0.09 nmol/10 cells [30]). The experimental results were consistent with the theoretical prediction from log *Cf* values that, compared with GlyF, conjugates **4g** and **4a** have better transmembrane behavior through passive diffusion. 

The injection of *RcANT15* in *Xenopus* oocytes significantly enhanced the uptake level of all three conjugates being tested, and the results correlated positively with the phloem mobility measured in *R. communis* seedlings. Compared with the control group, the uptake of conjugates **4g**, **4a**, and GlyF (control: 0.50, 0.20, and 0.36 nmol/10 cells [30], respectively) in oocytes injected with *RcANT15* cRNA was increased by about 0.25, 0.05, and 0.27 nmol/10 cells, respectively. This indicated that the amino acid carrier *RcANT15* did promote the uptake of all tested conjugates in *Xenopus* oocytes, but the significance may be distinct for different conjugates. The transport capability of *RcANT15* towards amino acid conjugates was responsible for the phloem mobility of GlyF, which was consistent with the conclusion from our previous study [15]. Although conjugated with the same amino acid substituent, conjugate **4a** did not show adequate improvement in the uptake amount comparable to GlyF, indicating that the derivatization position and other factors such as linker structure may still affect its ability to be transported by carriers. For conjugate **4g**, injection of *RcANT15* cRNA led to a similar enhancement in the uptake amount compared to GlyF, but with a higher amount being absorbed through passive diffusion, it showed the overall highest phloem mobility. Combined with the results from theoretical prediction, we can conclude that high efficiency in passive diffusion and high affinity to bind with an active carrier were both key aspects that made conjugate **4g** highly phloem mobile. 

## 3. Materials and Methods

### 3.1. Synthesis

#### 3.1.1. General Information

All reagents and solvents were purchased from Energy Chemical Co (Shanghai, China). Nuclear magnetic resonance (NMR) spectra were obtained using a Bruker AV-600 instrument (Bruker, Karlsruhe, Germany). Deuterated solvents were obtained from Cambridge Isotope Laboratories (Andover, MA, USA). DMSO-*d*_6_ and CDCl_3_ solvent peaks (2.50 and 7.26 ppm for ^1^H and 39.52 and 77.16 ppm for ^13^C, respectively) were used as internal chemical shift references. Mass spectrographic analysis was conducted on a Waters SYNAPTQ^TM^ (Waters, Milford, MA, USA). Analytical thin-layer chromatography (TLC) was carried out on percolated plates (silica gel GF254), and spots were visualized with ZF-20D ultraviolet analyzer (Qingdao Marine Chemical Ltd., Qingdao, China). Silica gel (200–300 mesh) was used for column chromatography.

#### 3.1.2. Synthesis of 5-Amino-1-[2,6-dichloro-4-(trifluoromethyl)phenyl)]-4-[(trifluoromethyl) sulfinyl)]-1*H*-pyrazole-3-carboxylic acid (**2**)

Fipronil (8.74 g, 20 mmol) was dissolved in acetic acid (50 mL). Concentrated (98%) sulfuric acid (25 mL) and water (20 mL) was then added, and the mixture was stirred at 120 °C for 1 h and then heated to reflux for an additional 2 h. After cooling to room temperature, 1 M aqueous solution of sodium hydroxide was added dropwise at 0 °C until the pH reached 10. Water was added to dissolve all precipitate, and the aqueous solution was washed with ethyl ether (3 × 200 mL). The aqueous layer was acidified with 1 M hydrochloric acid solution until the pH reached 1, and was then extracted with ethyl acetate (3 × 200 mL). The combined organic layer was washed with brine (3 × 200 mL), dried over anhydrous sodium sulphate, and evaporated under reduced pressure. The residue was recrystallized with mixture of ethyl acetate (5 mL) and petroleum ether (150 mL) twice and dried under vacuum to afford compound **2**. The ^1^H-NMR and ^13^C-NMR spectra of compound **2** can be found in Figures S1 and S2. Off-white solid, yield 72%, m.p. 154–156 °C. ^1^H-NMR (600 MHz, DMSO-*d*_6_) δ 13.75 (s, 1H), 8.27 (s, 2H), 6.81 (s, 2H). ^13^C-NMR (150 MHz, DMSO-*d*_6_) δ 161.74, 152.10, 143.75, 136.21, 135.93, 135.25, 133.05 (q, *J* = 33.8 Hz), 126.68–126.42, 126.16 (q, *J* = 340.7 Hz), 122.36 (q, *J* = 273.9 Hz), 88.63. ESI-HRMS calcd for C_12_H_6_Cl_2_F_6_N_3_O_3_S [M + H]^+^ 455.9411; found, 455.9424.

#### 3.1.3. Synthesis of Compound Series **3**

A mixture of amino acid methyl ester hydrochloride (15 mmol) and *N*-methylmorpholine (2.20 mL, 20 mmol) was dissolved in anhydrous dichloromethane (150 mL). The reaction mixture was then cooled to 0 °C, and compound **2** (4.56 g, 10 mmol), EDC∙HCl (2.88 g, 15 mmol), and DMAP (0.12 g, 1 mmol) were added successively. The resulting solution was stirred at room temperature for 3 h. After completion of the reaction according to TLC tracking, water was added and the aqueous layer was extracted three times with dichloromethane (3 × 50 mL). The combined organic layer was sequentially washed with saturated sodium bicarbonate solution, water, and brine. The organic layer was dried over anhydrous sodium sulphate and evaporated to obtain the residue, which was purified by column chromatography (petroleum/ethyl acetate, *v*/*v* = 3:1−6:1) to afford **3a**–**l** as white solids. The ^1^H-NMR and ^13^C-NMR spectra of compounds **3a**–**l** can be found in Figures S3–S26.

*Methyl{5-amino-1-[2,6-dichloro-4-(trifluoromethyl)phenyl]-4[(trifluoromethyl)sulfinyl]-1H-pyrazole-3-carbonyl}glycinate* (**3a**). White solid, yield 86%, m.p. 195–197 °C. ^1^H-NMR (600 MHz, Chloroform-*d*) δ 7.87 (s, 2H), 7.34–7.25 (m, 1H), 5.27 (s, 2H), 4.24–4.19 (m, 2H), 3.83 (d, *J* = 1.0 Hz, 3H). ^13^C-NMR (150 MHz, Chloroform-*d*) δ 169.51, 159.80, 151.31, 145.46, 136.72, 136.63, 134.79 (q, *J* = 34.7 Hz), 134.21, 126.33–126.24, 125.91 (q, *J* = 338.4 Hz), 121.82 (q, *J* = 272.3 Hz), 90.82, 52.45, 40.79. ESI-HRMS calcd for C_15_H_11_Cl_2_F_6_N_4_O_4_S [M + H]^+^ 526.9782; found, 526.9764.

*Methyl{5-amino-1-[2,6-dichloro-4-(trifluoromethyl)phenyl]-4-[(trifluoromethyl)sulfinyl]-1H-pyrazole-3-carbonyl}-d-alaninate* (**3b**). White solid, yield 81%, m.p. 233–235 °C. ^1^H-NMR (600 MHz, Chloroform-*d*) δ 7.81 (s, 2H), 7.28–7.15 (m, 1H), 5.20 (s, 2H), 4.76–4.57 (m, 1H), 3.76 (d, *J* = 4.9 Hz, 3H), 1.48 (dd, *J* = 9.3, 7.1 Hz, 3H). ^13^C-NMR (150 MHz, Chloroform-*d*) δ 173.02, 159.65, 151.72, 146.06, 137.21, 137.13, 135.19 (q, *J* = 34.7 Hz), 134.66, 126.81–126.59, 126.34 (q, *J* = 338.4 Hz), 122.24 (q, *J* = 274.6 Hz), 91.17, 52.95, 48.37, 18.73. ESI-HRMS calcd for C_16_H_13_Cl_2_F_6_N_4_O_4_S [M + H]^+^ 540.9939; found, 540.9925.

*Methyl{5-amino-1-[2,6-dichloro-4-(trifluoromethyl)phenyl]-4-[(trifluoromethyl)sulfinyl]-1H-pyrazole-3-carbonyl}-l-valinate* (**3c**). White solid, yield 82%, m.p. 202–204 °C. ^1^H-NMR (600 MHz, Chloroform-*d*) δ 7.84 (s, 2H), 7.30–7.22 (m, 1H), 5.25 (s, 2H), 4.65 (dd, *J* = 8.9, 4.9 Hz, 1H), 3.77 (s, 3H), 2.31–2.23 (m, 1H), 1.01 (d, *J* = 6.9 Hz, 3H), 0.98 (d, *J* = 6.9 Hz, 3H). ^13^C-NMR (150 MHz, Chloroform-*d*) δ 171.60, 159.56, 151.32, 145.59, 136.77, 136.63, 134.72 (q, *J* = 34.6 Hz), 134.28, 126.32–126.21, 125.91 (q, *J* = 338.5 Hz), 121.84 (q, *J* = 273.9 Hz), 90.73, 57.01, 52.18, 31.53, 18.86, 17.83. ESI-HRMS calcd for C_18_H_17_Cl_2_F_6_N_4_O_4_S [M + H]^+^ 569.0251; found, 569.0240.

*Methyl{5-amino-1-[2,6-dichloro-4-(trifluoromethyl)phenyl]-4-[(trifluoromethyl)sulfinyl]-1H-pyrazole-3-carbonyl}-l-leucinate* (**3d**). White solid, yield 78%, m.p. 199–201 °C. ^1^H-NMR (600 MHz, Chloroform-*d*) δ 7.26 (s, 2H), 6.46 (d, *J* = 8.4 Hz, 1H), 4.63 (s, 2H), 4.30–4.02 (m, 1H), 3.20 (s, 3H), 1.19–1.10 (m, 2H), 1.10–1.03 (m, 1H), 0.39 (d, *J* = 1.4 Hz, 3H), 0.38 (d, *J* = 1.4 Hz, 3H). ^13^C-NMR (150 MHz, Chloroform-*d*) δ 173.13, 159.83, 151.71, 146.09, 137.15, 137.14, 135.20 (q, *J* = 34.4 Hz), 134.67, 126.87–126.62, 126.29 (q, *J* = 336.3 Hz), 122.25 (q, *J* = 273.7 Hz), 91.36, 52.79, 50.99, 41.77, 25.21, 23.03, 22.24. ESI-HRMS calcd for C_19_H_19_Cl_2_F_6_N_4_O_4_S [M + H]^+^ 583.0408; found, 583.0397.

*Methyl{5-amino-1-[2,6-dichloro-4-(trifluoromethyl)phenyl]-4-[(trifluoromethyl)sulfinyl]-1H-pyrazole-3-carbonyl}-l-isoleucinate* (**3e**). White solid, yield 76%, m.p. 202–203 °C. ^1^H-NMR (600 MHz, Chloroform-*d*) δ 7.80 (s, 2H), 7.22 (d, *J* = 8.7 Hz, 1H), 5.25 (s, 2H), 4.65 (dd, *J* = 8.8, 4.9 Hz, 1H), 3.73 (d, *J* = 1.1 Hz, 3H), 2.04–1.92 (m, 1H), 1.47 (ddd, *J* = 13.8, 7.4, 4.8 Hz, 1H), 1.23–1.17 (m, 2H), 0.93 (dd, *J* = 7.2, 3.3 Hz, 6H). ^13^C-NMR (150 MHz, Chloroform-*d*) δ 171.53, 159.41, 151.38, 145.62, 136.76, 136.65, 134.80 (q, *J* = 34.6 Hz), 134.32, 126.30–126.18, 125.91 (q, *J* = 338.5 Hz), 121.84 (q, *J* = 273.9 Hz), 90.63, 56.29, 52.07, 38.12, 25.20, 15.33, 11.47. ESI-HRMS calcd for C_19_H_19_Cl_2_F_6_N_4_O_4_S [M + H]^+^ 583.0408; found, 583.0401.

*Methyl{5-amino-1-[2,6-dichloro-4-(trifluoromethyl)phenyl]-4-[(trifluoromethyl)sulfinyl]-1H-pyrazole-3-carbonyl}-l-threoninate* (**3f**). White solid, yield 75%, m.p. 232–235 °C. ^1^H-NMR (600 MHz, DMSO-*d*_6_) δ 8.32–8.22 (m, 2H), 7.87 (dd, *J* = 12.4, 8.5 Hz, 1H), 6.86 (d, *J* = 4.3 Hz, 2H), 5.23 (dd, *J* = 6.8, 3.4 Hz, 1H), 4.41 (td, *J* = 8.1, 3.4 Hz, 1H), 4.28–4.03 (m, 1H), 3.66 (d, *J* = 11.6 Hz, 3H), 1.10 (dd, *J* = 15.9, 6.4 Hz, 3H). ^13^C-NMR (150 MHz, DMSO-*d*_6_) δ 170.84, 160.28, 152.56, 145.67, 136.65, 136.36, 135.41, 133.45 (q, *J* = 33.0 Hz), 126.91–126.75, 126.39 (q, *J* = 340.5 Hz), 122.59 (q, *J* = 273.7 Hz), 88.13, 66.50, 58.23, 52.33, 20.42. ESI-HRMS calcd for C_17_H_15_Cl_2_F_6_N_4_O_5_S [M + H]^+^ 571.0045; found, 571.0049.

*Methyl{5-amino-1-[2,6-dichloro-4-(trifluoromethyl)phenyl]-4-[(trifluoromethyl)sulfinyl]-1H-pyrazole-3-carbonyl}-l-serinate (***3g**). White solid, yield 76%, m.p. 222–224 °C. ^1^H-NMR (600 MHz, Chloroform-*d*) δ 7.82 (s, 2H), 7.55 (d, *J* = 7.7 Hz, 1H), 5.20 (s, 2H), 4.88–4.67 (m, 1H), 4.06 (dd, *J* = 11.3, 3.8 Hz, 1H), 3.97 (dd, *J* = 11.3, 3.5 Hz, 1H), 3.80 (d, *J* = 1.0 Hz, 3H), 2.42 (s, 1H). ^13^C-NMR (150 MHz, Chloroform-*d*) δ 170.61, 160.41, 151.80, 145.99, 137.20, 137.17, 135.36 (q, *J* = 34.5 Hz), 134.73, 126.81–126.72, 126.44 (q, *J* = 337.0 Hz), 122.30 (q, *J* = 273.8 Hz), 91.76, 63.50, 54.88, 53.21. ESI-HRMS calcd for C_16_H_13_Cl_2_F_6_N_4_O_5_S [M + H]^+^ 556.9888; found, 556.9888.

*Methyl{5-amino-1-[2,6-dichloro-4-(trifluoromethyl)phenyl]-4-[(trifluoromethyl)sulfinyl]-1H-pyrazole-3-carbonyl}-l-phenylalaninate* (**3h**). White solid, yield 71%, m.p. 115–117 °C. ^1^H-NMR (600 MHz, Chloroform-*d*) δ 7.83–7.77 (m, 2H), 7.29–7.25 (m, 1H), 7.27–7.17 (m, 3H), 7.14–7.09 (m, 2H), 5.29 (s, 2H), 4.95 (dt, *J* = 8.3, 6.0 Hz, 1H), 3.70 (s, 3H), 3.18 (qd, *J* = 13.8, 6.0 Hz, 2H). ^13^C-NMR (150 MHz, Chloroform-*d*) δ 171.28, 159.34, 151.54, 145.57, 136.88, 136.76, 135.60, 134.85 (q, *J* = 34.6 Hz), 134.45, 129.37, 128.69, 127.30, 126.48–126.40, 126.08 (q, *J* = 338.5 Hz), 122.03 (q, *J* = 274.0 Hz), 90.69, 53.28, 52.49, 38.32. ESI-HRMS calcd for C_22_H_17_Cl_2_F_6_N_4_O_4_S [M + H]^+^ 617.0251; found, 617.0255.

*Methyl{5-amino-1-[2,6-dichloro-4-(trifluoromethyl)phenyl]-4-[(trifluoromethyl)sulfinyl]-1H-pyrazole-3-carbonyl}-l-tyrosinate* (**3i**). White solid, yield 72%, m.p. 103–106 °C. ^1^H-NMR (600 MHz, Chloroform-*d*) δ 7.82–7.77 (m, 2H), 7.17 (dd, *J* = 16.2, 8.3 Hz, 1H), 6.99–6.91 (m, 2H), 6.75–6.63 (m, 2H), 5.18 (d, *J* = 6.4 Hz, 2H), 4.90 (ddt, *J* = 14.1, 8.2, 5.9 Hz, 1H).3.71 (d, *J* = 4.8 Hz, 3H), 3.19–3.01 (m, 2H). ^13^C-NMR (150 MHz, Chloroform-*d*) δ 171.40, 159.26, 155.09, 151.36, 145.38, 136.68, 136.50, 134.65 (q, *J* = 34.3 Hz), 134.14, 130.17, 126.79, 126.35–126.12, 125.88 (q, *J* = 338.5 Hz), 121.80 (q, *J* = 273.9 Hz), 115.44, 90.41, 53.34, 52.41, 37.14. ESI-HRMS calcd for C_22_H_17_Cl_2_F_6_N_4_O_5_S [M + H]^+^ 633.0201; found, 633.0184.

*Methyl{5-amino-1-[2,6-dichloro-4-(trifluoromethyl)phenyl]-4-[(trifluoromethyl)sulfinyl]-1H-pyrazole-3-carbonyl}-l-tryptophanate* (**3j**). White solid, yield 70%, m.p. 94–97 °C. ^1^H-NMR (600 MHz, Chloroform-*d*) δ 8.11 (d, *J* = 12.2 Hz, 1H), 7.80–7.73 (m, 2H), 7.49 (ddq, *J* = 18.7, 7.9, 0.9 Hz, 1H), 7.31 (ddt, *J* = 8.1, 5.6, 0.9 Hz, 1H), 7.29–7.25 (m, 1H), 7.16–7.09 (m, 1H), 7.06–6.96 (m, 2H), 5.16 (d, *J* = 2.7 Hz, 2H), 5.03 (ddt, *J* = 10.5, 8.0, 5.5 Hz, 1H), 3.67 (d, *J* = 12.4 Hz, 3H), 3.45–3.34 (m, 2H). ^13^C-NMR (150 MHz, Chloroform-*d*) δ 171.69, 159.47, 151.42, 145.81, 136.98, 136.83, 136.20, 134.89 (q, *J* = 33.8 Hz), 134.42, 127.63, 126.56–126.29, 126.14 (q, *J* = 338.4 Hz), 122.92, 122.31, 120.23 (q, *J* = 274.0 Hz), 119.83, 118.74, 111.29, 109.95, 90.97, 53.04, 52.59, 28.11. ESI-HRMS calcd for C_24_H_18_Cl_2_F_6_N_5_O_4_S [M + H]^+^ 656.0361. found, 656.0389.

*Dimethyl{5-amino-1-[2,6-dichloro-4-(trifluoromethyl)phenyl]-4-[(trifluoromethyl)sulfinyl]-1H-pyrazole-3-carbonyl}-l-aspartate* (**3k**). White solid, yield 78%, m.p. 78–81 °C. ^1^H-NMR (600 MHz, Chloroform-*d*) δ 7.85–7.76 (m, 2H), 7.69–7.57 (m, 1H), 5.18 (s, 2H), 4.95 (ddt, *J* = 19.8, 8.2, 4.7 Hz, 1H), 3.77 (d, *J* = 1.7 Hz, 3H), 3.68 (d, *J* = 2.7 Hz, 3H), 3.08 (ddd, *J* = 17.1, 5.6, 4.6 Hz, 1H), 2.92 (ddd, *J* = 31.8, 17.2, 4.7 Hz, 1H). ^13^C-NMR (150 MHz, Chloroform-*d*) δ 171.01, 170.39, 159.57, 151.29, 145.34, 136.82, 136.56, 134.77 (q, *J* = 34.6 Hz), 134.24, 126.41–126.14, 125.93 (q, *J* = 339.6 Hz), 121.84 (q, *J* = 273.9 Hz), 90.86, 52.90, 52.06, 48.38, 36.00. ESI-HRMS calcd for C_18_H_15_Cl_2_F_6_N_4_O_6_S [M + H]^+^ 598.9993; found, 598.9980.

*Dimethyl{5-amino-1-[2,6-dichloro-4-(trifluoromethyl)phenyl]-4-[(trifluoromethyl)sulfinyl]-1H-pyrazole-3-carbonyl}-l-glutamate* (**3l**). White solid, yield 77%, m.p. 73–75 °C. ^1^H-NMR (600 MHz, Chloroform-*d*) δ 7.83–7.79 (m, 2H), 7.31–7.25 (m, 1H), 5.21 (s, 2H), 4.72 (tdd, *J* = 8.0, 5.3, 2.3 Hz, 1H), 3.76 (d, *J* = 4.3 Hz, 3H), 3.64 (d, *J* = 8.5 Hz, 3H), 2.52–2.34 (m, 2H), 2.32 – 2.02 (m, 2H). ^13^C-NMR (150 MHz, Chloroform-*d*) δ 173.19, 171.82, 159.99, 151.81, 145.80, 137.19, 137.00, 135.14 (q, *J* = 34.6 Hz), 134.68, 126.79–126.54, 126.43 (q, *J* = 336.6 Hz), 122.24 (q, *J* = 273.8 Hz), 91.09, 52.98, 52.14, 51.84, 30.36, 27.87. ESI-HRMS calcd for C_19_H_17_Cl_2_F_6_N_4_O_6_S [M + H]^+^ 613.0150; found, 613.0136.

#### 3.1.4. Synthesis of Compound Series **4**

Lithium hydroxide (0.63 g, 15 mmol) was added to a solution of compound **3** (5 mmol) in water (10 mL) and THF (20 mL), and the mixture was stirred for 2 h at room temperature. The solution was then adjusted to pH 2 with 1 M hydrochloric acid. The organic solvent was then removed via rotary evaporation, and the residual aqueous solution was extracted with ethyl acetate (3 × 25 mL). The combined ethyl acetate layer was sequentially washed with saturated sodium bicarbonate solution, water, and brine. The organic layer was dried over anhydrous sodium sulphate and evaporated to obtain a residue, which was purified by column chromatography (petroleum/ethyl acetate/acetic acid, *v*/*v*/*v* = 2:1:0.005–4:1:0.005) to afford **4a**–**l** as white solids. The ^1^H-NMR and ^13^C-NMR spectra of compounds **4a**–**l** can be found in Figures S27–S50.

*{5-Amino-1-[2,6-dichloro-4-(trifluoromethyl)phenyl]-4-[(trifluoromethyl)sulfinyl]-1H-pyrazole-3-carbonyl}glycine* (**4a**). White solid, yield 89%, m.p. 107–109 °C. ^1^H-NMR (600 MHz, Chloroform-*d*) δ 12.60 (s, 1H), 8.72–8.65 (m, 1H), 8.35–8.20 (m, 2H), 6.81 (s, 2H), 3.93–3.72 (m, 2H). ^13^C-NMR (150 MHz, Chloroform-*d*) δ 172.82, 159.94, 151.33, 145.36, 136.69, 136.63, 134.83 (q, *J* = 34.8 Hz), 134.09, 126.39–126.20, 125.80 (q, *J* = 338.5 Hz), 121.64 (q, *J* = 272.6 Hz), 90.54, 40.71. ESI-HRMS calcd for C_14_H_9_Cl_2_F_6_N_4_O_4_S [M + H]^+^ 512.9626; found, 512.9624.

*{5-Amino-1-[2,6-dichloro-4-(trifluoromethyl)phenyl]-4-[(trifluoromethyl)sulfinyl]-1H-pyrazole-3-carbonyl}-d-alanine* (**4b**). White solid, yield 87%, m.p. 138–140 °C. ^1^H-NMR (600 MHz, DMSO-*d*_6_) δ 12.67 (s, 1H), 8.58 (d, *J* = 7.4 Hz, 1H), 8.27 (d, *J* = 2.4 Hz, 2H), 6.79 (s, 2H), 4.34 (t, *J* = 7.3 Hz, 1H), 1.35 (d, *J* = 7.3 Hz, 3H). ^13^C-NMR (150 MHz, DMSO-*d*_6_) δ 166.30, 152.47, 144.84, 138.06, 129.13, 128.90, 127.49, 126.48 (q, *J* = 34.4 Hz), 118.57–118.49, 118.46 (q, *J* = 338.6 Hz), 114.69 (q, *J* = 273.3 Hz), 80.85, 52.49, 8.75. ESI-HRMS calcd for C_15_H_11_Cl_2_F_6_N_4_O_4_S [M + H]^+^ 526.9782; found, 526.9780.

*{5-Amino-1-[2,6-dichloro-4-(trifluoromethyl)phenyl]-4-[(trifluoromethyl)sulfinyl]-1H-pyrazole-3-carbonyl}-l-valine* (**4c**). White solid, yield 88%, m.p. 221–223 °C. ^1^H-NMR (600 MHz, DMSO-*d*_6_) δ 12.88 (s, 1H), 8.27 (d, *J* = 3.1 Hz, 2H), 8.05 (d, *J* = 8.1 Hz, 1H), 6.82 (s, 2H), 4.18 (dd, *J* = 8.1, 6.1 Hz, 1H), 2.21–2.13 (m, 1H), 0.90 (t, *J* = 6.6 Hz, 6H). ^13^C-NMR (150 MHz, DMSO-*d*_6_) δ 164.90, 152.48, 144.80, 137.78, 128.95, 128.74, 127.26, 126.39 (q, *J* = 34.4 Hz), 118.47–118.39, 118.39 (q, *J* = 337.6 Hz), 114.61 (q, *J* = 272.1 Hz), 80.74, 49.52, 22.94, 10.30, 9.15. ESI-HRMS calcd for C_17_H_15_Cl_2_F_6_N_4_O_4_S [M + H]^+^ 555.0095; found, 555.0075.

*{5-Amino-1-[2,6-dichloro-4-(trifluoromethyl)phenyl]-4-[(trifluoromethyl)sulfinyl]-1H-pyrazole-3-carbonyl}-l-leucine* (**4d**). White solid, yield 88%, m.p. 216–218 °C. ^1^H-NMR (600 MHz, DMSO-*d*_6_) δ 12.66 (s, 1H), 8.60 (d, *J* = 8.1 Hz, 1H), 8.27 (d, *J* = 10.6 Hz, 2H), 6.80 (s, 2H), 4.34 (ddd, *J* = 10.5, 8.0, 4.1 Hz, 1H), 1.78 (ddd, *J* = 12.9, 10.6, 4.0 Hz, 1H), 1.62–1.47 (m, 2H), 0.86 (d, *J* = 6.3 Hz, 3H), 0.83 (d, *J* = 6.3 Hz, 3H). ^13^C-NMR (150 MHz, DMSO-*d*_6_) δ 165.72, 152.14, 144.14, 137.44, 128.46, 128.20, 126.81, 125.78 (q, *J* = 34.5 Hz), 117.95 (q, *J* = 338.7 Hz), 117.92–117.71, 113.92 (q, *J* = 265.0 Hz), 80.19, 42.16, 31.73, 16.35, 13.56, 12.04. ESI-HRMS calcd for C_18_H_17_Cl_2_F_6_N_4_O_4_S [M + H]^+^ 569.0251; found, 569.0227.

*{5-Amino-1-[2,6-dichloro-4-(trifluoromethyl)phenyl]-4-[(trifluoromethyl)sulfinyl]-1H-pyrazole-3-carbonyl}-l-isoleucine* (**4e**). White solid, yield 87%, m.p. 226–227 °C. ^1^H-NMR (600 MHz, Chloroform-*d*) δ 12.82 (s, 1H), 7.81 (s, 2H), 7.15 (d, *J* = 8.6 Hz, 1H), 5.18 (s, 2H), 4.67 (dd, *J* = 8.6, 4.8 Hz, 1H), 2.05–1.98 (m, 2H), 1.51 (ddt, *J* = 15.0, 7.7, 3.9 Hz, 1H), 0.98 (d, *J* = 6.9 Hz, 3H), 0.93 (t, *J* = 7.4 Hz, 3H). ^13^C-NMR (150 MHz, Chloroform-*d*) δ 175.75, 159.79, 151.50, 145.82, 136.95, 136.94, 135.02 (q, *J* = 34.6 Hz), 134.42, 126.66–126.36, 126.14 (q, *J* = 338.6 Hz), 122.05 (q, *J* = 273.9 Hz), 91.01, 56.45, 37.89, 25.20, 15.59, 11.69. ESI-HRMS calcd for C_18_H_17_Cl_2_F_6_N_4_O_4_S [M + H]^+^ 569.0251; found, 569.0228.

*{5-Amino-1-[2,6-dichloro-4-(trifluoromethyl)phenyl]-4-[(trifluoromethyl)sulfinyl]-1H-pyrazole-3-carbonyl}-l-threonine* (**4f**). White solid, yield 88%, m.p. 137–139 °C. ^1^H-NMR (600 MHz, DMSO-*d*_6_) δ 12.82 (s, 1H), 8.29–8.25 (m, 2H), 7.70 (dd, *J* = 8.7, 6.4 Hz, 1H), 6.85 (s, 2H), 4.32–4.26 (m, 1H), 4.18 (dq, *J* = 6.1, 3.2 Hz, 1H), 1.08 (dd, *J* = 17.1, 6.4 Hz, 3H). ^13^C-NMR (150 MHz, DMSO-*d*_6)_ δ 171.04, 159.38, 151.78, 145.19, 135.92, 135.56, 134.69, 132.66 (q, *J* = 33.7 Hz), 126.28–126.15, 125.93 (q, *J* = 338.2 Hz), 121.93 (q, *J* = 273.8 Hz), 87.39, 65.96, 57.28, 20.24. ESI-HRMS calcd for C_16_H_13_Cl_2_F_6_N_4_O_5_S [M + H]^+^ 556.9888; found, 556.9870.

*{5-Amino-1-[2,6-dichloro-4-(trifluoromethyl)phenyl]-4-[(trifluoromethyl)sulfinyl]-1H-pyrazole-3-carbonyl}-l-serine* (**4g**). White solid, yield 89%, m.p. 188–190 °C. ^1^H-NMR (600 MHz, DMSO-*d*_6_) δ 12.83 (s, 1H), 8.30–8.26 (m, 2H), 8.13 (d, *J* = 7.7 Hz, 1H), 6.84 (s, 2H), 4.36 (dt, *J* = 8.2, 4.2 Hz, 1H), 3.81 (dd, *J* = 11.3, 5.0 Hz, 1H), 3.72 (dd, *J* = 11.3, 3.5 Hz, 1H). ^13^C-NMR (150 MHz, DMSO-*d*_6_) δ 170.87, 159.12, 151.69, 145.24, 135.92, 135.59, 134.70, 132.64 (q, *J* = 33.3 Hz), 126.29–126.08, 125.57 (q, *J* = 338.3 Hz), 121.92 (q, *J* = 273.2 Hz), 87.40, 60.43, 54.33. ESI-HRMS calcd for C_15_H_11_Cl_2_F_6_N_4_O_5_S [M + H]^+^ 542.9731; found, 542.9733.

*{5-Amino-1-[2,6-dichloro-4-(trifluoromethyl)phenyl]-4-[(trifluoromethyl)sulfinyl]-1H-pyrazole-3-carbonyl}-l-phenylalanine* (**4h**). White solid, yield 81%, m.p. 240–242 °C. ^1^H-NMR (600 MHz, DMSO-*d*_6_) δ 12.85 (s, 1H), 8.46 (d, *J* = 8.0 Hz, 1H), 8.30–8.24 (m, 2H), 7.31–7.12 (m, 5H), 6.79 (s, 2H), 4.54 (td, *J* = 8.4, 5.2 Hz, 1H), 3.24–3.01 (m, 2H). ^13^C-NMR (150 MHz, DMSO-*d*_6_) δ 171.97, 159.26, 151.75, 145.44, 137.41, 136.13, 135.70, 134.89, 132.74 (q, *J* = 33.8 Hz), 128.82, 127.86, 126.36–126.19, 126.13, 125.82 (q, *J* = 341.0 Hz), 122.05 (q, *J* = 273.9 Hz), 87.40, 53.16, 35.58. ESI-HRMS calcd for C_21_H_15_Cl_2_F_6_N_4_O_4_S [M + H]^+^ 603.0095; found, 603.0119.

*{5-Amino-1-[2,6-dichloro-4-(trifluoromethyl)phenyl]-4-[(trifluoromethyl)sulfinyl]-1H-pyrazole-3-carbonyl}-l-tyrosine* (**4i**). White solid, yield 75%, m.p. 231–234 °C. ^1^H-NMR (600 MHz, DMSO-*d*_6_) δ 12.95 (s, 1H), 8.48 (d, *J* = 8.1 Hz, 1H), 8.33–8.27 (m, 2H), 7.34–7.19 (m, 5H), 6.83 (s, 2H), 4.60 (td, *J* = 8.5, 5.3 Hz, 1H), 3.17 (td, *J* = 14.2, 8.8 Hz, 2H). ^13^C-NMR (150 MHz, DMSO-*d*_6_) δ 172.10, 159.40, 151.91, 145.59, 137.53, 136.27, 135.84, 135.04, 132.89 (q, *J* = 33.8 Hz), 128.97, 128.00, 126.49–126.33, 126.28, 125.96 (q, *J* = 340.8 Hz), 122.19 (q, *J* = 273.9 Hz), 87.57, 53.30, 35.74. ESI-HRMS calcd for C_21_H_15_Cl_2_F_6_N_4_O_5_S [M + H]^+^ 619.0045; found, 619.0023.

*{5-Amino-1-[2,6-dichloro-4-(trifluoromethyl)phenyl]-4-[(trifluoromethyl)sulfinyl]-1H-pyrazole-3-carbonyl}-l-tryptophan* (**4j**). White solid, yield 77%, m.p. 110–112 °C. ^1^H-NMR (600 MHz, Chloroform-*d*) δ 12.95 (s, 1H), 10.83 (d, *J* = 2.4 Hz, 1H), 8.25 (ddd, *J* = 8.4, 1.9, 0.7 Hz, 2H), 8.23 (d, *J* = 7.8 Hz, 1H), 7.49 (dd, *J* = 7.9, 1.0 Hz, 1H), 7.30 (dt, *J* = 8.1, 0.9 Hz, 1H), 7.10 (d, *J* = 2.4 Hz, 1H), 7.03 (ddd, *J* = 8.1, 6.9, 1.2 Hz, 1H), 6.91 (ddd, *J* = 8.0, 7.0, 1.0 Hz, 1H), 6.78 (s, 2H), 4.59 (td, *J* = 7.6, 5.4 Hz, 1H), 3.26 (dd, *J* = 6.3, 3.2 Hz, 2H). ^13^C-NMR (150 MHz, DMSO-*d*_6_) δ 172.19, 159.01, 151.61, 145.39, 136.00, 135.66, 134.73, 132.60 (q, *J* = 33.4 Hz), 129.25, 127.37 (q, *J* = 338.5 Hz), 126.87, 126.25–125.98, 123.16, 121.91 (q, *J* = 271.9 Hz), 120.48, 117.98, 117.80, 110.96, 109.39, 87.31, 52.66, 28.62. ESI-HRMS calcd for C_23_H_16_Cl_2_F_6_N_5_O_4_S [M + H]^+^ 642.0204; found, 642.0215.

*{5-Amino-1-[2,6-dichloro-4-(trifluoromethyl)phenyl]-4-[(trifluoromethyl)sulfinyl]-1H-pyrazole-3-carbonyl}-l-aspartic acid* (**4k**). White solid, yield 78%, m.p. 127–129 °C. ^1^H-NMR (600 MHz, DMSO-*d*_6_) δ 12.68 (s, 2H), 8.58–8.52 (m, 1H), 8.28–8.25 (m, 2H), 6.82 (s, 2H), 4.69–4.64 (m, 1H), 2.88–2.63 (m, 2H). ^13^C-NMR (150 MHz, DMSO-*d*_6_) δ 172.34, 172.27, 159.73, 152.42, 146.14, 136.71, 136.39, 135.49, 133.42 (q, *J* = 33.4 Hz), 126.97–126.85, 126.36 (q, *J* = 339.1 Hz), 122.77 (q, *J* = 272.1 Hz), 88.22, 48.90, 36.02. ESI-HRMS calcd for C_16_H_11_Cl_2_F_6_N_4_O_6_S [M + H]^+^ 570.9681; found, 570.9656.

*{5-Amino-1-[2,6-dichloro-4-(trifluoromethyl)phenyl]-4-[(trifluoromethyl)sulfinyl]-1H-pyrazole-3-carbonyl}-l-glutamic acid* (**4l**). White solid, yield 71%, m.p. 121–123 °C. ^1^H-NMR (600 MHz, DMSO-*d*_6)_ δ 12.43 (s, 2H), 8.73–8.58 (m, 1H), 8.25 (d, *J* = 4.8 Hz, 2H), 6.80 (d, *J* = 5.3 Hz, 2H), 4.42–4.30 (m, 1H), 2.36–2.23 (m, 2H), 2.01–1.85 (m, 2H). ^13^C-NMR (150 MHz, DMSO-*d*_6)_ δ 174.08, 172.97, 160.24, 152.34, 146.18, 136.69, 136.42, 135.49, 133.36 (q, *J* = 32.1 Hz), 126.92–126.70, 126.40 (q, *J* = 340.9 Hz), 121.67 (q, *J* = 272.1 Hz), 88.18, 51.73, 30.56, 25.98. ESI-HRMS calcd for C_17_H_13_Cl_2_F_6_N_4_O_6_S [M + H]^+^ 584.9837; found, 584.9808.

### 3.2. Physicochemical Properties

Physicochemical properties [17] (molecular weight (MW), octanol/water partitioning coefficient (log K*_o/w_*)) of the twelve amino acid–fipronil conjugates were predicted using ACD/Labs version 14.0 software (classical method, ACD/Labs, Toronto, ON, Canada). The ionization constant in aqueous solution (pK_a_) was calculated with Marvin Sketch (version 6.3.0, Chemaxon Ltd., Budapest, Hungary). The results are summarized in Table 2. 

### 3.3. Plant Materials

Castor bean seeds (*R. communis*) No. 9 were purchased from the Agricultural Science Academy of Zibo, Shandong, China. The seeds were placed in humid cotton for 24 h at 27 °C prior to sowing in wet vermiculite. Seedlings were nurtured as previously described [37]. Seedlings after 6 days of growth were selected for further experiments. 

### 3.4. Phloem Sap Collection

The method to collect phloem sap was similar as that recently described [38,39]. Cotyledons, from which the endosperm had been removed, were put in wells of a 12-well cell culture plate and incubated in buffer solution (containing 20 mM MES (pH = 5.6), 0.25 mM MgCl_2_, and 0.5 mM CaCl_2_) supplemented with 100 μM amino acid–fipronil conjugates. Roots of seedlings were immersed in 0.5 mM CaCl_2_ solution. After 1 h of pre-incubation, the hypocotyls were severed in the hook region for phloem exudation. Phloem sap was collected at 1 h intervals for 5 h, and was saved at 4 °C until analysis. After being diluted with pure water to four times its volume, the phloem sap was quantified using an Agilent HPLC system (1260, Agilent Technologies Inc., Santa Clara, CA, USA).

### 3.5. Animal Materials

The *Xenopus laevis* were purchased from the Institute of Biochemistry and Cell Biology, SIBS (Shanghai, China) and incubated in a clean glass sink. Feedstuff (Tianbangmeiwa 2) was used 2 to 3 times per week. 

Mature and healthy oocytes (stage V–VII) were treated with 1 mg/mL collagenase S-1 in washing buffer (96 mM NaCl, 2 mM KCl, 5 mM MgCl_2_, and 5 mM HEPES, pH = 7.6) for 1–2 h at room temperature. Oocytes were later microinjected with 18.4 ng cRNAs [31].

### 3.6. *Xenopus* Heterologous Expression System and Drug Uptake Experiment

Total RNA extraction of castor beans was performed using an E.A.N.A.^TM^ Plant RNA Kit (OMEGA, Norcross, GA, USA). The ratio of A260/A280 was 1.8–2.0 during the experiment, which was suitable for subsequent experiments. The concentration of extracted RNA was diluted to 250 ng/μL. A quantity of 2 μg of extracted RNA was used for cDNA synthesis using a PrimeScript^TM^ II 1st Strand cDNA Synthesis Kit (TAKARA, Tokyo, Japan). A full-length coding sequence of *RcANT15* was cloned into eukaryotic expression vector pT7Ts. cRNAs were synthesized from linearized vectors using an mMESSAGE mMACHINETM T7 Transcription Kit (Ambion, Austin, TX, USA). 

Stage V or stage VI oocytes from *Xenopus laevis* were treated with 1 mg/mL collagenase type 1 in washing buffer (96 mM NaCl, 2 mM KCl, 1 mM MgCl_2_, and 5 mM HEPES, pH = 7.6) for 60–90 min at room temperature. A quantity of 18.4 ng cRNA of *RcANT15* was later microinjected into oocytes (with Nuclease-Free water as control). After injection, oocytes were cultured in Ringer’s buffer (96 mM NaCl, 2 mM KCl, 1 mM MgCl_2_, 1.8 mM CaCl_2_, and 5 mM HEPES, pH = 7.6) supplemented with 5% dialyzed horse serum, 50 mg/mL tetracycline, 100 mg/mL streptomycin, and 2.5 mM sodium pyruvate at 16 °C for 2–6 days.

Ten oocytes that were injected with cRNA of *RcANT15* or Nuclease-Free water were pre-incubated in 500 μL Kulori buffer (90 mM NaCl, 1 mM KCl, 1 mM CaCl_2_, 1 mM MgCl_2_, and 5 mM MES, pH = 5.6) for 5 min to ensure intracellular steady-state pH. Then, the oocytes were put into solution containing 0.1 mM of test compound and treated for 1 h. After incubation, oocytes were washed three times with Kulori buffer and solubilized in 10% SDS to dissociate the cell. The total volume was adjusted to 100 μL after being concentrated in vacuo for 4 h. The uptake of tested compounds was determined by HPLC. Each treatment was repeated at least three times [40].

### 3.7. Analytical Methods

A C 8 reversed-phase column (5 μm, 250 × 4.6 mm inner diameter, Agilent Technologies Inc., Santa Clara, CA, USA) were used for separations at 30 °C. The solvent system comprised acetonitrile and water (60:40, *v*/*v*) containing 0.1% TFA. The flow rate of the solvent system was 1 mL/min, and the injection volume was 10 μL.

## 4. Conclusions

A new series of phloem-mobile fipronil derivatives (**4a**–**l**) were designed and synthesized by conjugating amino acids with fipronil molecules at the 3-position on the pyrazole ring. Among them, conjugate **4g** presented the best phloem mobility, and the concentration of **4g** in phloem sap of *R. communis* seedlings was fivefold that of a previously reported glycinergic–fipronil conjugate (GlyF). Results from prediction with log *Cf* values and uptake experiments with *Xenopus* oocytes demonstrated that the phloem loading process of **4g** involves both passive diffusion and active carriers such as *RcANT15*. However, compared with GlyF, for which the phloem loading process was primarily carrier mediated, passive diffusion may have played a more important role for conjugate **4g** in addition to the involvement of carrier systems. Thus, the enhanced phloem mobility of conjugate **4g** compared to that of GlyF was probably due to its higher hydrophilicity. Overall, this study provides another example that conjugating amino acids to existing pesticide structures is a feasible and efficient strategy to acquire phloem mobility in non-phloem-mobile pesticides. It also suggested that due to the distinct physicochemical properties, the phloem loading process of different series of conjugates may occur via different mechanisms. Thus, optimizations to enable better passive diffusion may be a potential strategy to obtain pesticides with enhanced phloem mobility.

## Figures and Tables

**Table 1:** Concentrations of compounds **4a**–**l** and GlyF in phloem sap of castor bean seedlings.

Compounds	Conjugated Amino Acid	Concentration in Phloem Sap (μM) ^a^
**4a**	Glycine (Gly)	15.00 ± 2.50
**4b**	Alanine (Ala)	35.00 ± 4.00
**4c**	Valine (Val)	27.00 ± 4.00
**4d**	Leucine (Leu)	15.00 ± 1.50
**4e**	Isoleucine (Ile)	ND ^b^
**4f**	Threonine (Thr)	18.00 ± 2.60
**4g**	Serine (Ser)	52.00 ± 5.80
**4h**	Phenylalanine (Phe)	6.11 ± 0.80
**4i**	Tyrosine (Tyr)	12.00 ± 2.00
**4j**	Tryptophan (Trp)	8.70 ± 2.00
**4k**	Aspartic acid (Asp)	41.77 ± 5.00
**4l**	Glutamic acid (Glu)	20.00 ± 3.00
GlyF	Glycine (Gly)	10.14 ± 0.30 [15]

^a^ Phloem sap was collected after cotyledons were soaked in buffer solution containing 100 μM of tested conjugates at pH 5.6 for 6 h. Each datapoint is the mean of 12 seedlings ± SE (*n* = 4); ^b^ “ND” means not detected.

**Table 2:** Molecular weight, Log K*_o/w_*, and Log *Cf* of compounds **4a**–**l**. ^a^

Fipronil Analog	R	pK_a_^b^	Molecular Weight (g/mol)	Log K*_o/w_* ^c^	Log *Cf* ^d^
**4a**	H	2.8	513.2	2.65	−0.36
**4b**	CH_3_	2.9	527.23	3	0.11
**4c**	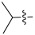	3	555.28	3.88	−3.44
**4d**	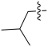	3.1	569.31	4.41	−18.2
**4e**	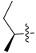	3.1	569.31	4.41	−18.2
**4f**	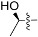	2.9	557.25	2.6	−0.15
**4g**	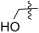	2.8	543.23	2.25	−0.59
**4h**	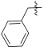	3.2	603.32	4.68	−39.58
**4i**	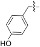	3.1	619.32	3.95	−4.42
**4j**	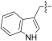	3.4	642.36	4.61	−32.43
**4k**	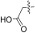	2.9	571.24	3.01	−0.19
**4l**	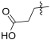	2.9	585.26	2.24	−0.52

^a^ Plant parameters are for a short plant *(L* = 15 cm, *I* = 5 cm, *r* = 5 μm, *v_0_* = 1.8 cm min^−1^). The membrane parameters are *a =* 1.20 and *b* = −7.50. The pH of the phloem sap was taken as 8.0 and that of the surrounding apoplast as 6.0. Log *Cf* values are evaluated at *s* = 13.5 cm; ^b^ Marvin pK_a_ macro constants; ^c^ Log K***_o/w_*** values were calculated with ACD/Labs version 14.0 software (classical method); ^d^ Log *Cf* values calculated with Marvin pK_a_ macro constants.

## Supplementary Materials

Supplementary materials are available online.
