# Supplementary material for: Synthesis of Novel Amino Acid–Fipronil Conjugates and Study on Their Phloem Loading Mechanism
Source: Molecules. 2018 Mar 28;23(4):778. doi: 10.3390/molecules23040778 (PMC6017586; doi:10.3390/molecules23040778)
Supplement: Supplementary file 1 [file molecules-23-00778-s001.pdf]

molecules

Supplementary Materials

Synthesis of Novel Amino Acid-Fipronil Conjugates and Study on their Phloem Loading Mechanism

Qingqing Sheng[1,2], Xinxin Liu[1,2], Yun Xie[1,2], Fei Lin[1,2], Zhixiang Zhang[1,2], Chen Zhao[1,2] * and Hanhong Xu[1,2] *

[1] State Key Laboratory for Conservation and Utilization of Subtropical Agro-Bioresources, South China Agricultural University, Guangzhou 510642, China; shengqing277@163.com (Q.S.); 15913194557@163.com (X.L.); xieyun91@foxmail.com (Y.X.); resistanc@scau.edu.cn (F.L.); zdsys@scau.edu.cn (Z.Z.)

[2] Key Laboratory of Natural Pesticide and Chemical Biology, Ministry of Education, South China Agricultural University, Guangzhou 510642, China

* Correspondence: zhaoc@scau.edu.cn (C.Z.); hhxu@scau.edu.cn (H.X.); Tel.: +86-20-8528-5127 (H.X.)

Figures S1-S50. ^{1}H NMR and ^{13}C NMR spectra of all compounds

Table S1. The fatality rates of **4a-l** and fipronil against *Plutella xylostella*

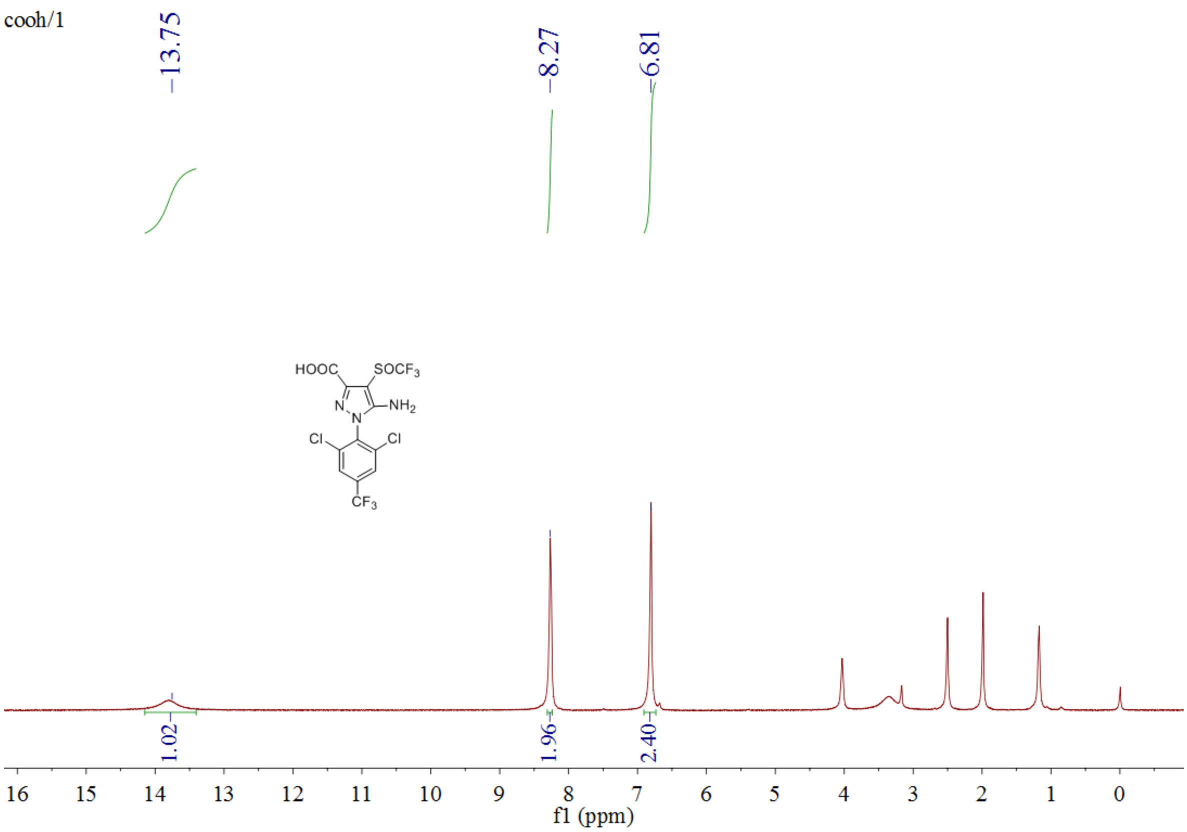

Figure S1 ^{1}H NMR spectra of **2** in DMSO-d_6

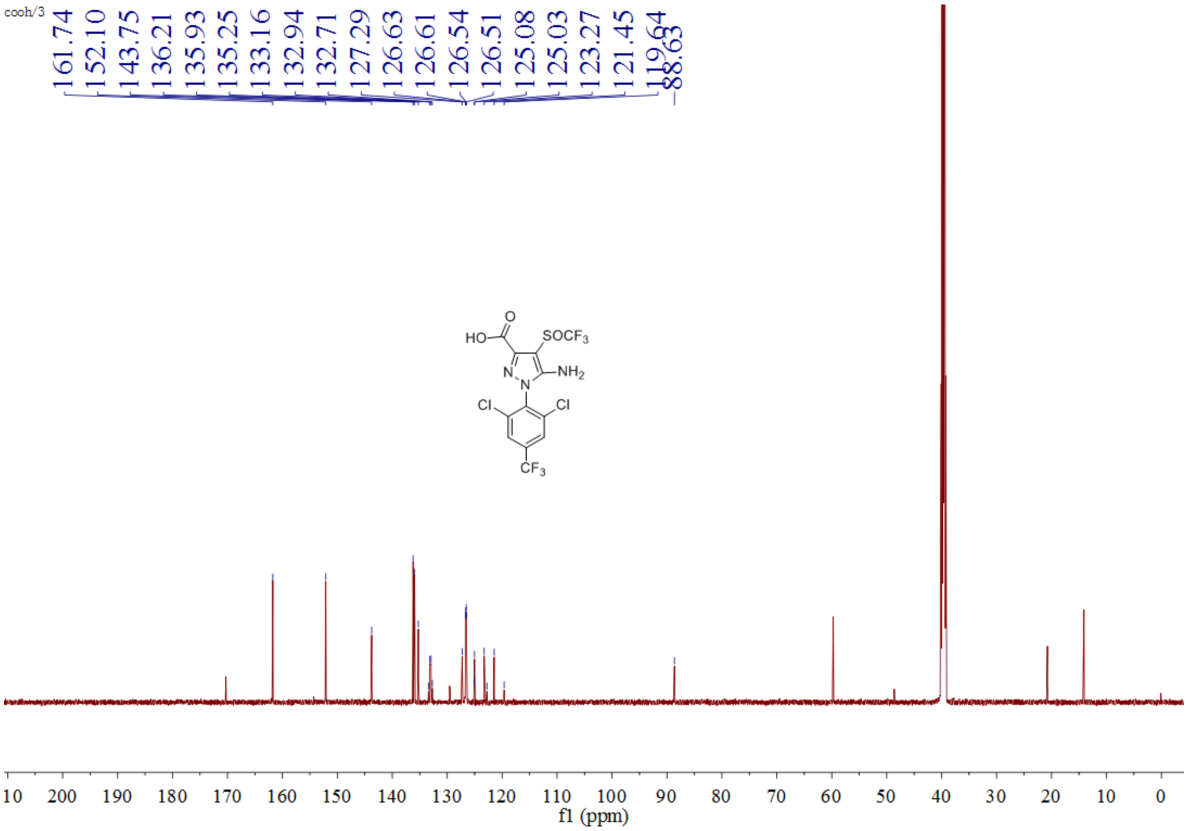

Figure S2 ^{13}C NMR spectra of **2** in DMSO-d_6

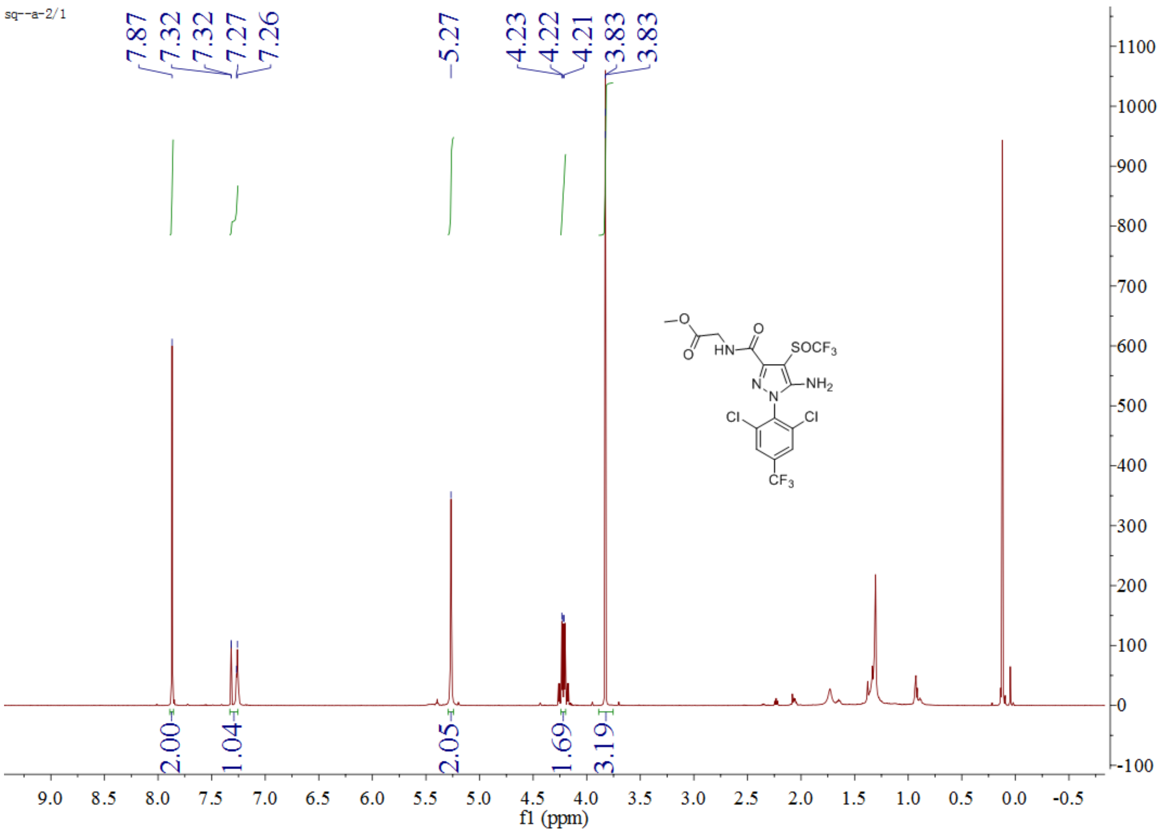

Figure S3 ^{1}H NMR spectra of **3a** in Chloroform-*d*

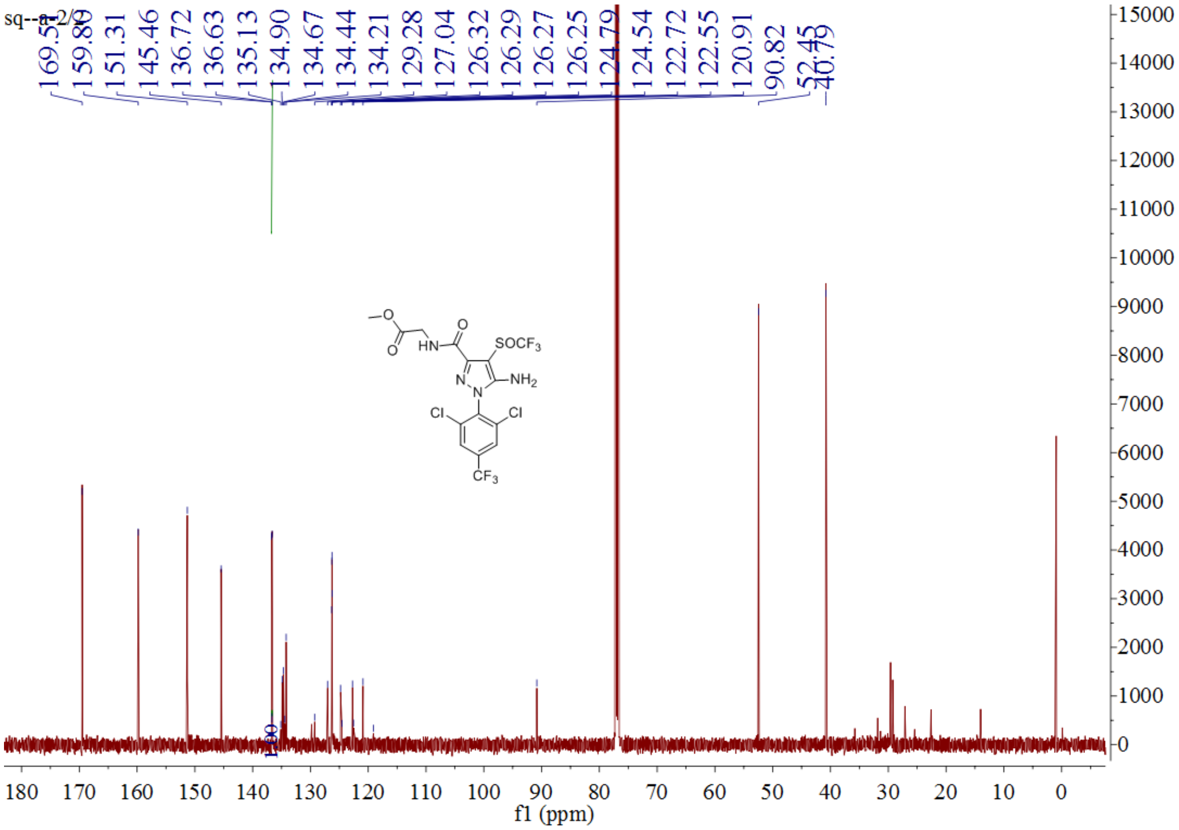

Figure S4 ^{13}C NMR spectra of **3a** in Chloroform-*d*

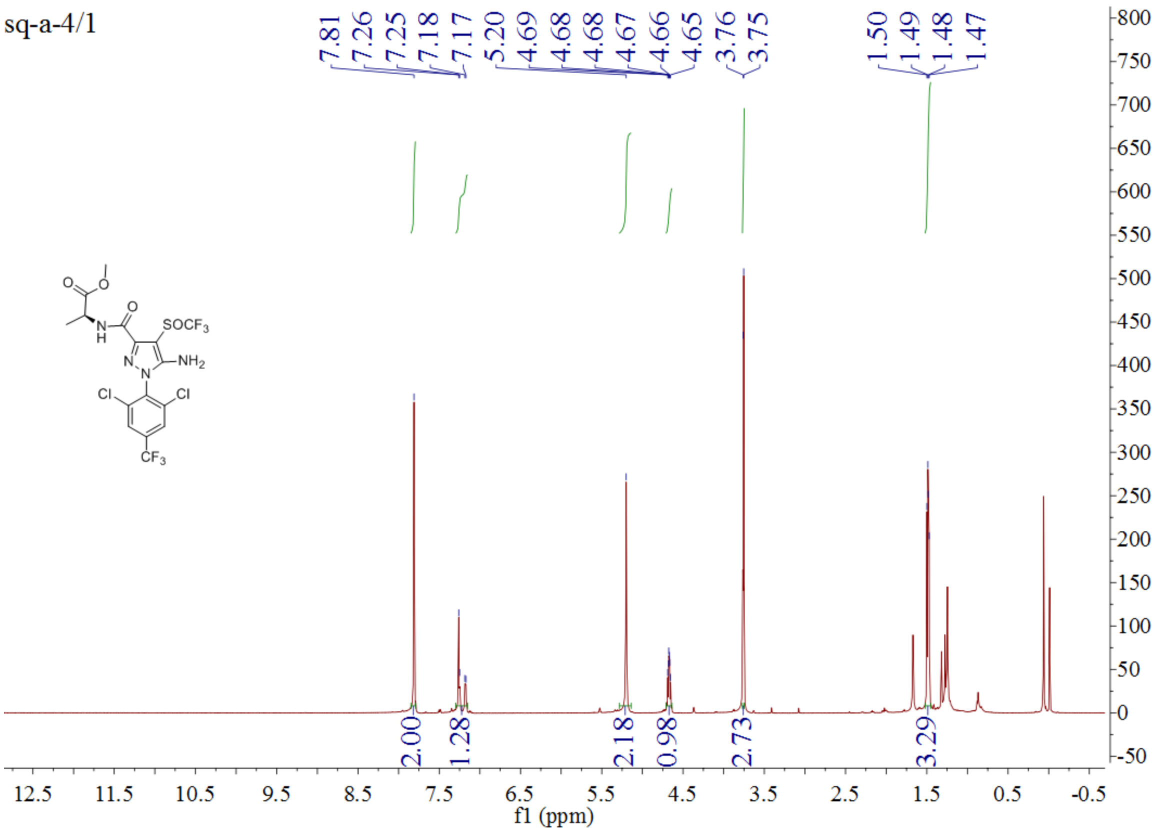

Figure S5 ^{1}H NMR spectra of **3b** in Chloroform-*d*

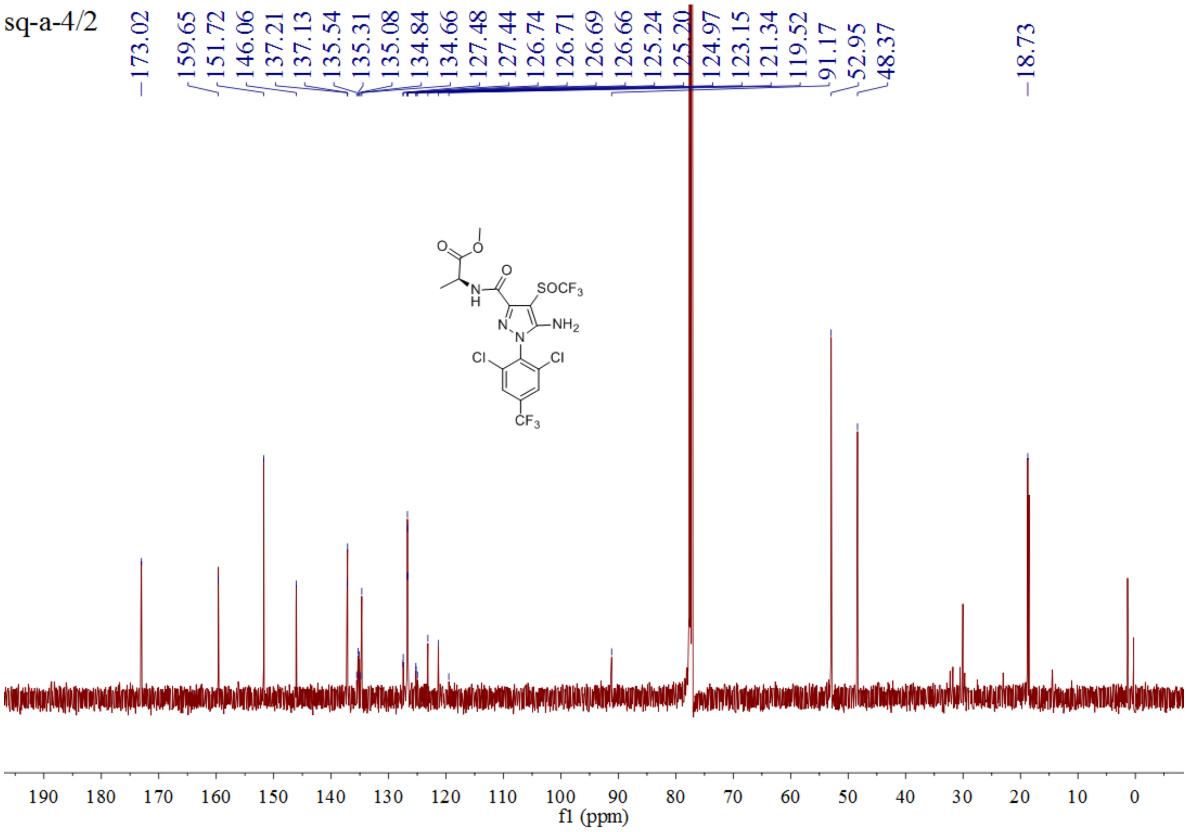

Figure S6 ^{13}C NMR spectra of **3b** in Chloroform-*d*

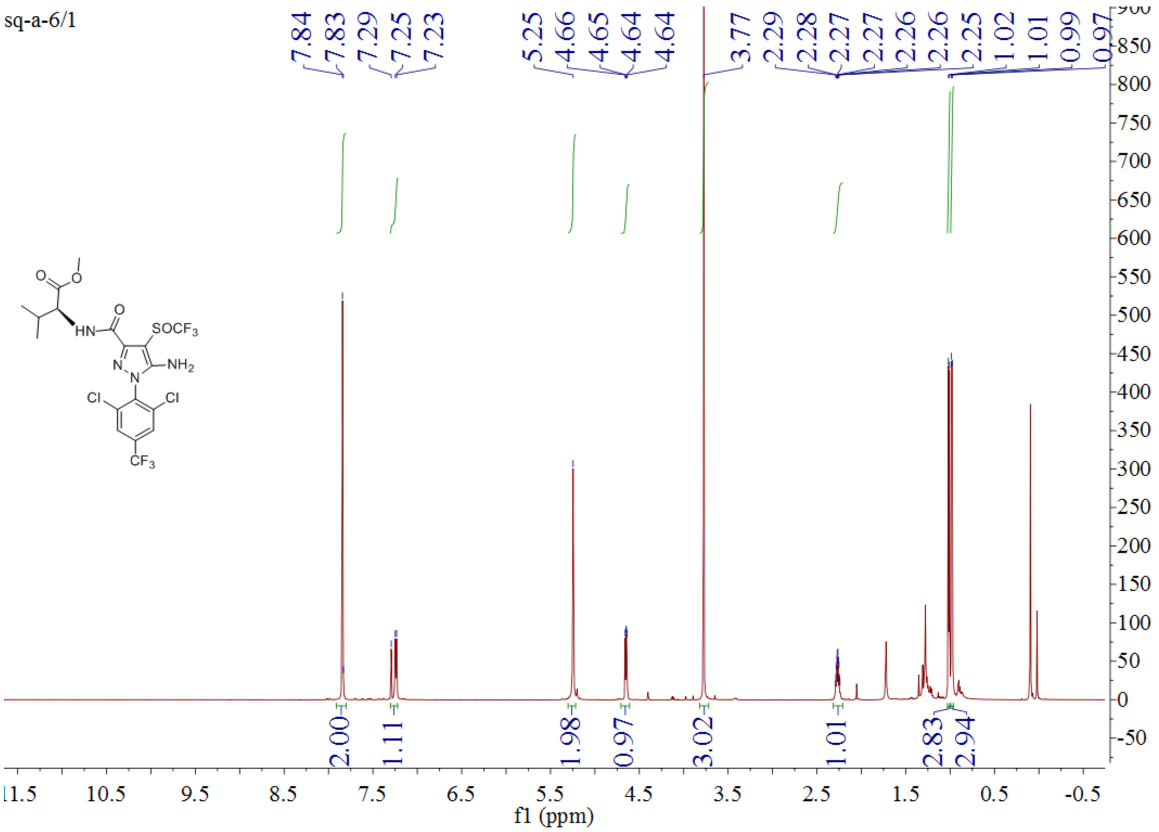

Figure S7 ^{1}H NMR spectra of **3c** in Chloroform-*d*

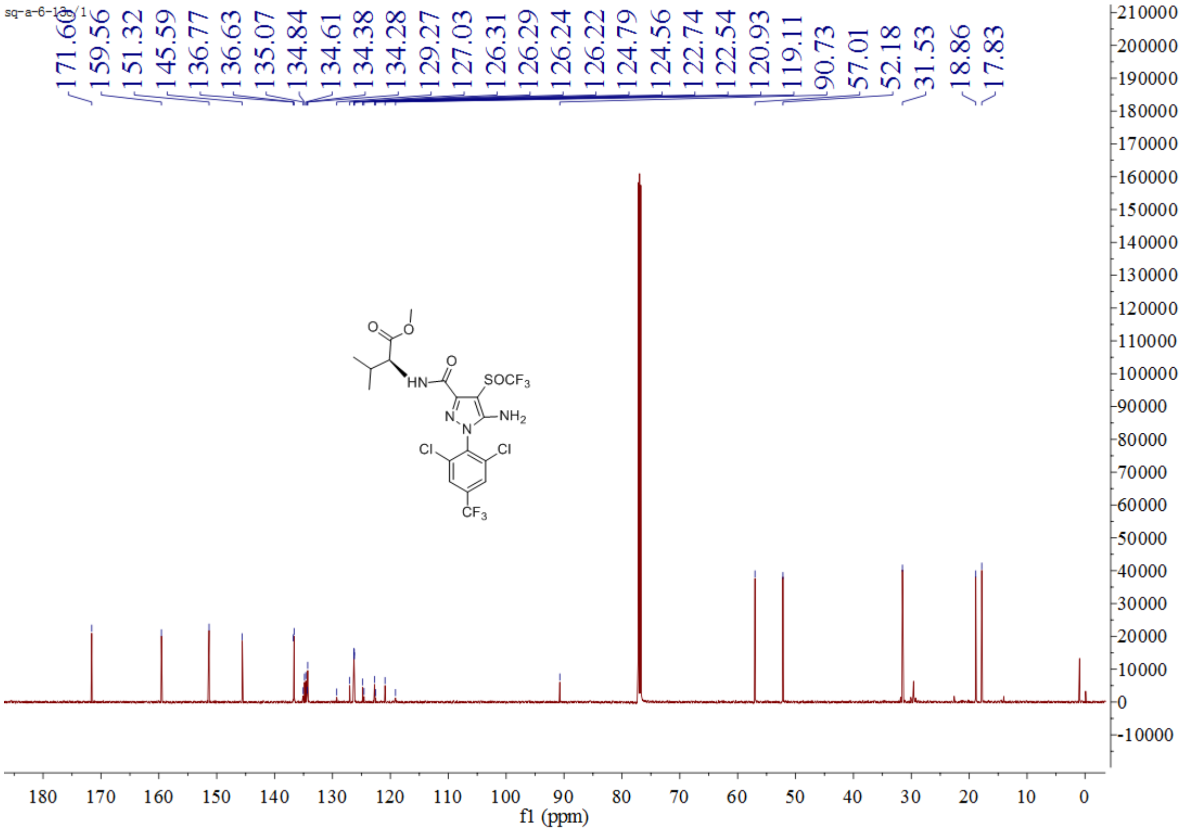

Figure S8 ^{13}C NMR spectra of **3c** in Chloroform-*d*

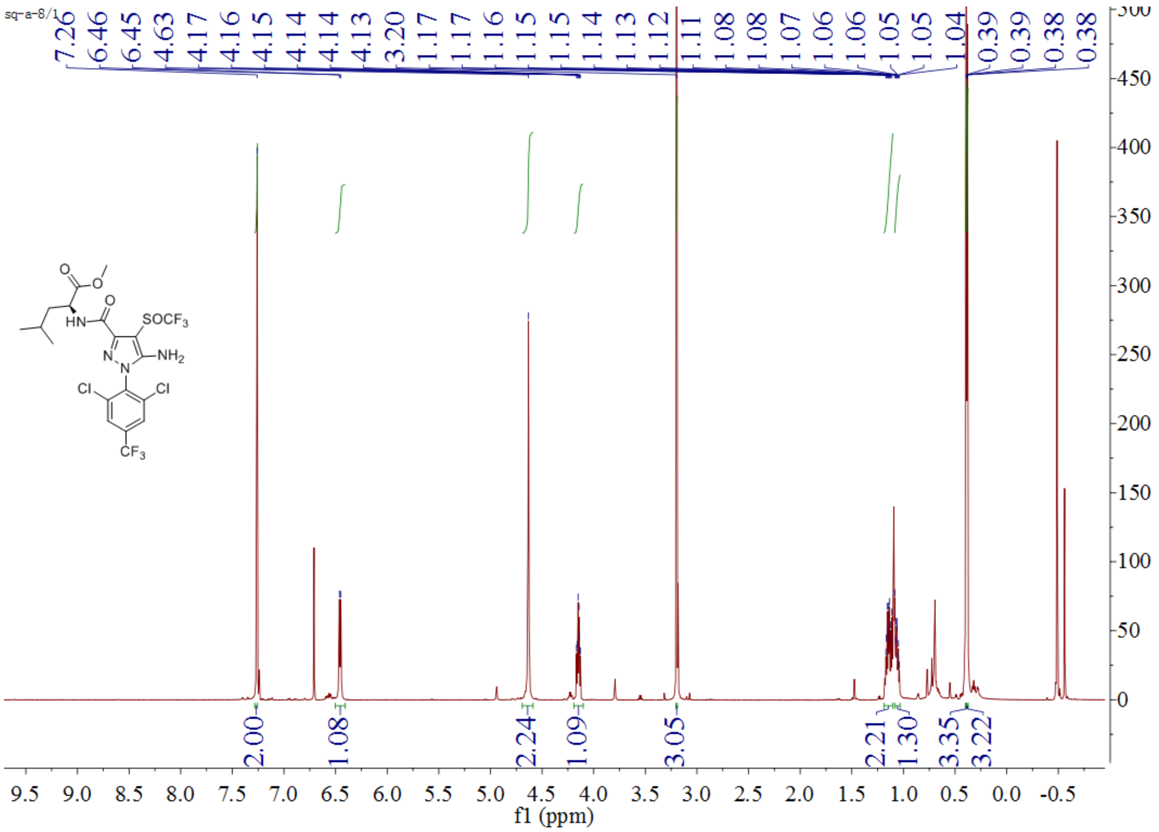

Figure S9 ^{1}H NMR spectra of **3d** in Chloroform-*d*

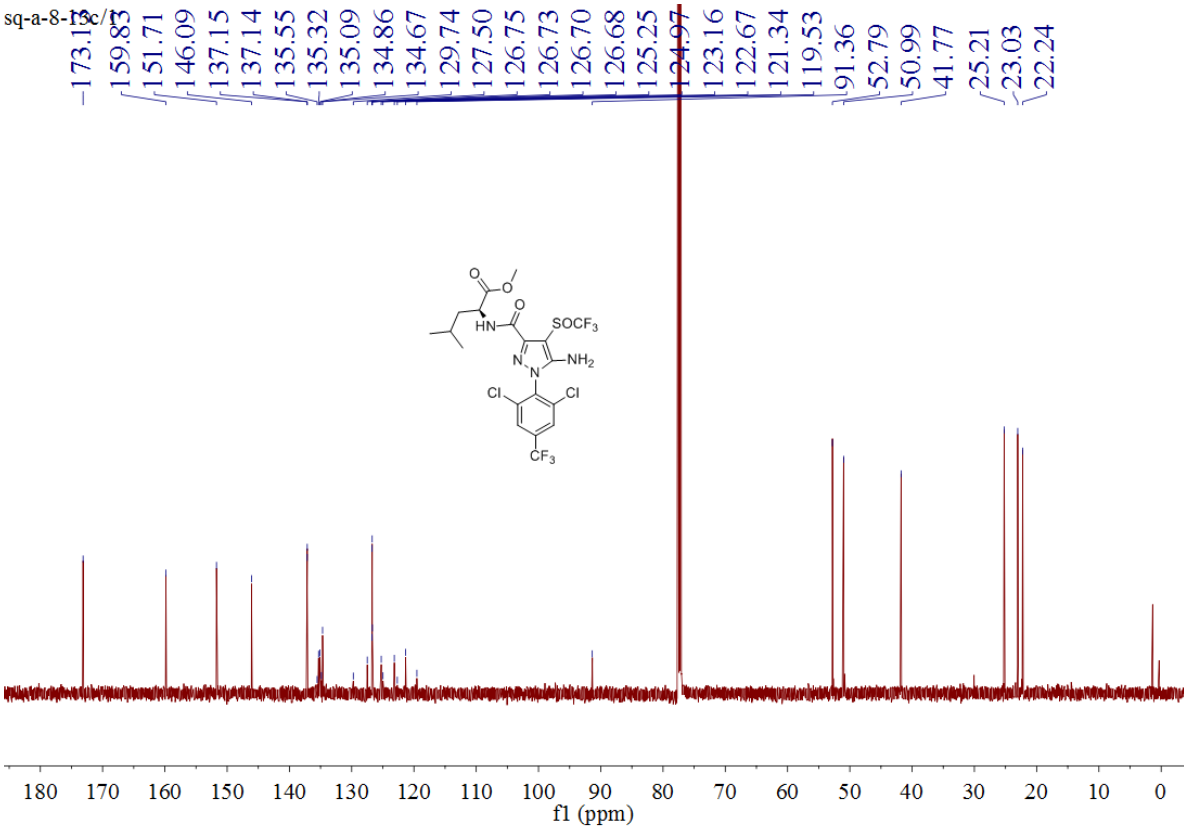

Figure S10 ^{13}C NMR spectra of **3d** in Chloroform-*d*

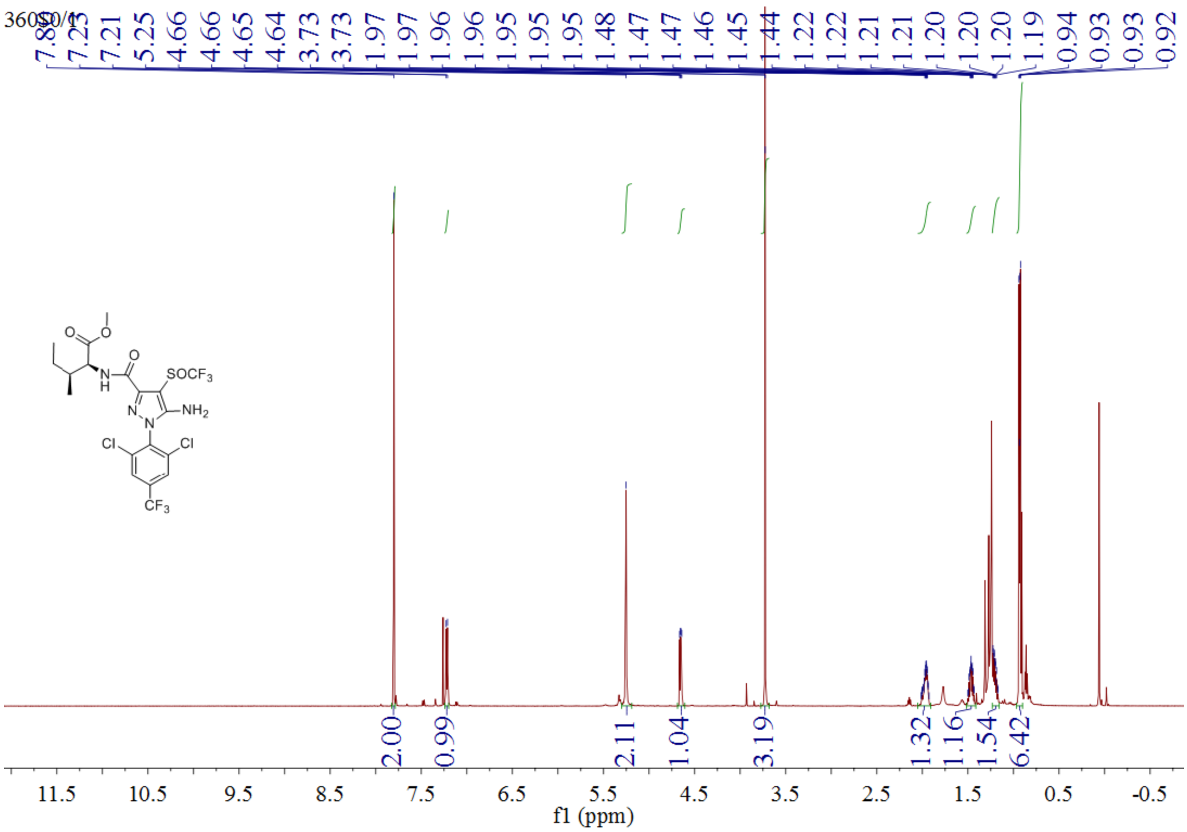

Figure S11 ^{1}H NMR spectra of **3e** in Chloroform-*d*

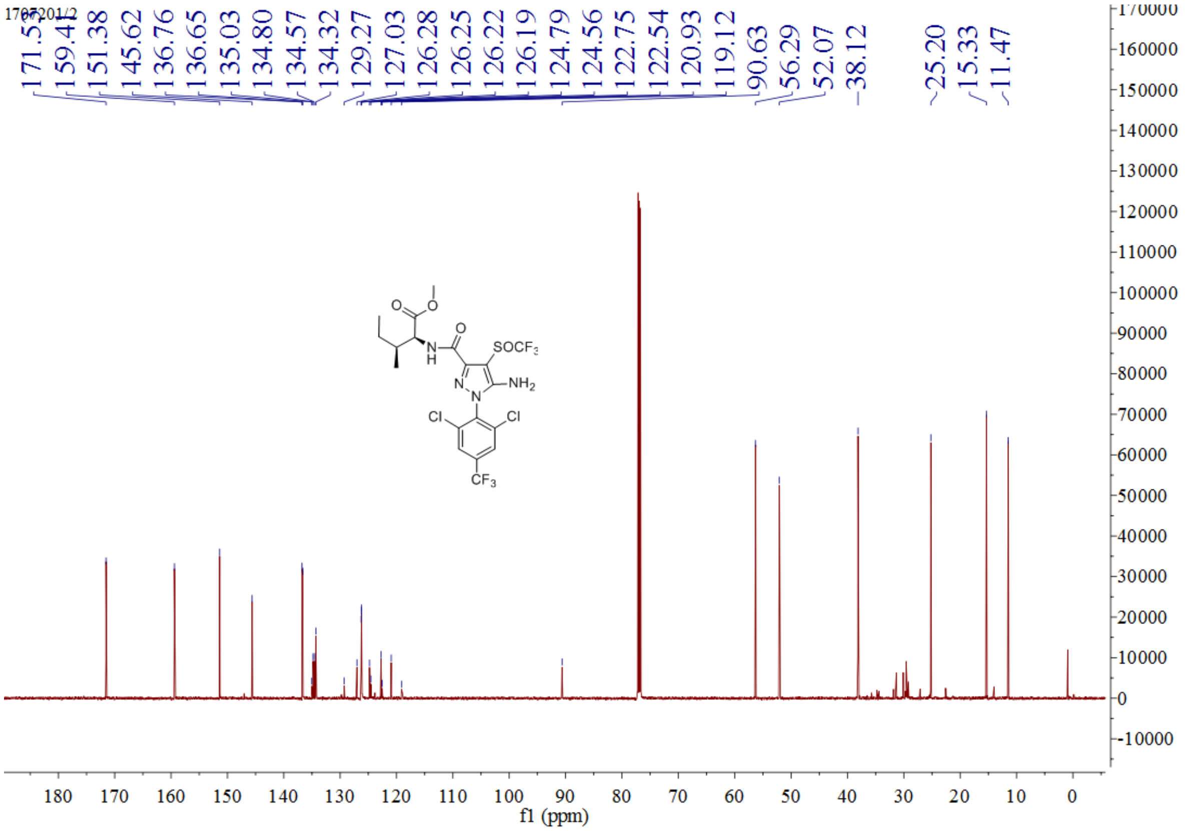

Figure S12 ^{13}C NMR spectra of **3e** in Chloroform-*d*

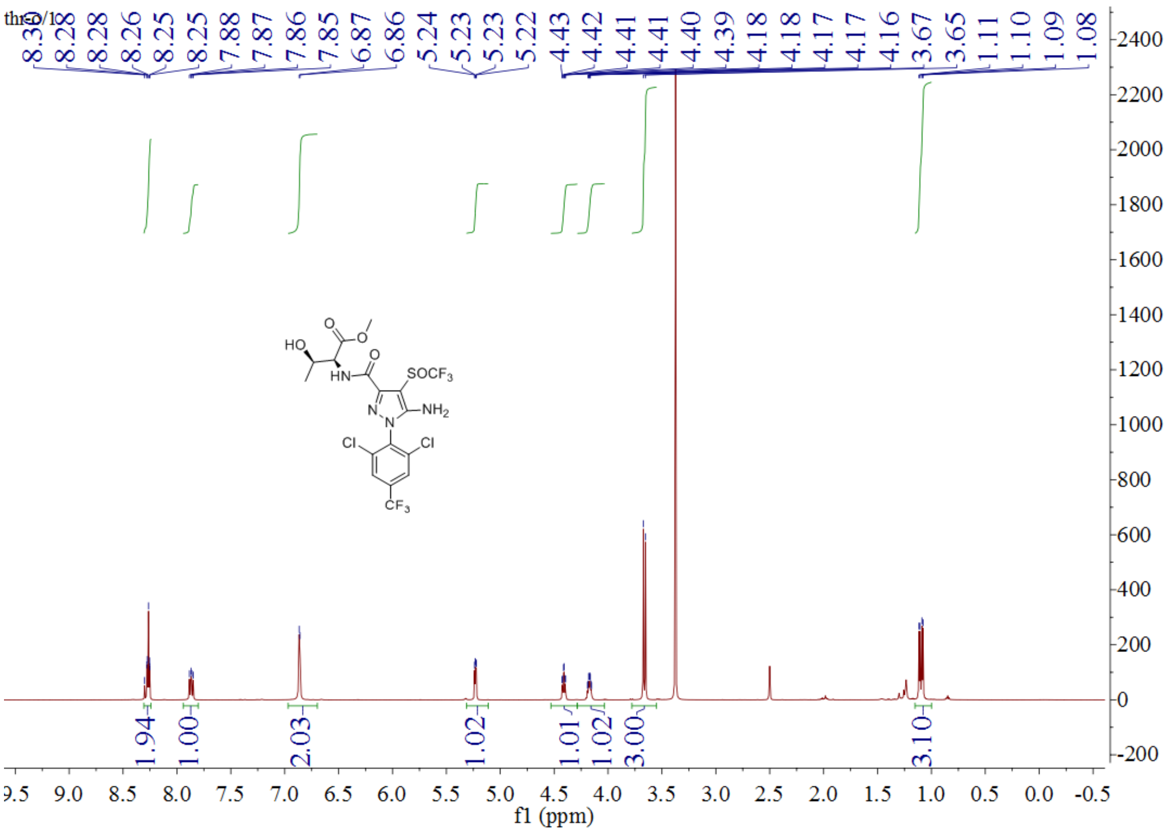

Figure S13 ^{1}H NMR spectra of **3f** in DMSO-d_6

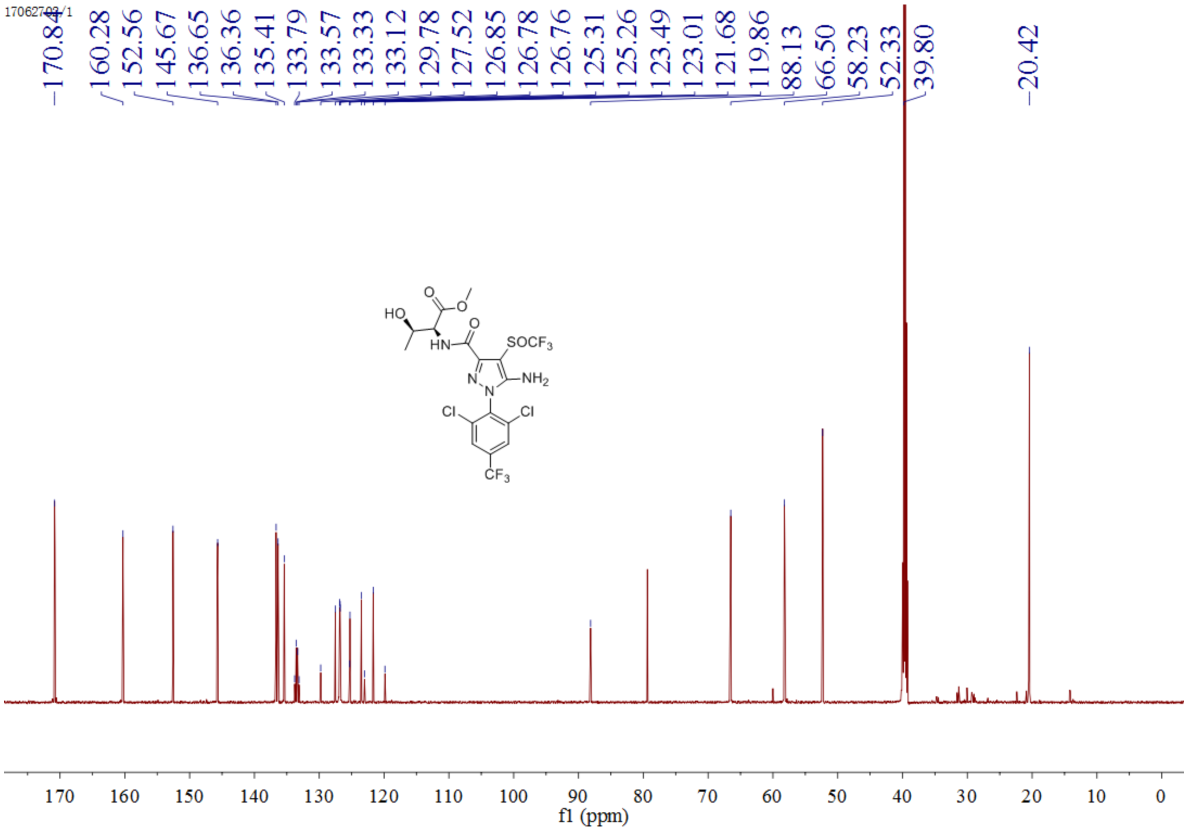

Figure S14 ^{13}C NMR spectra of **3f** in DMSO-d_6

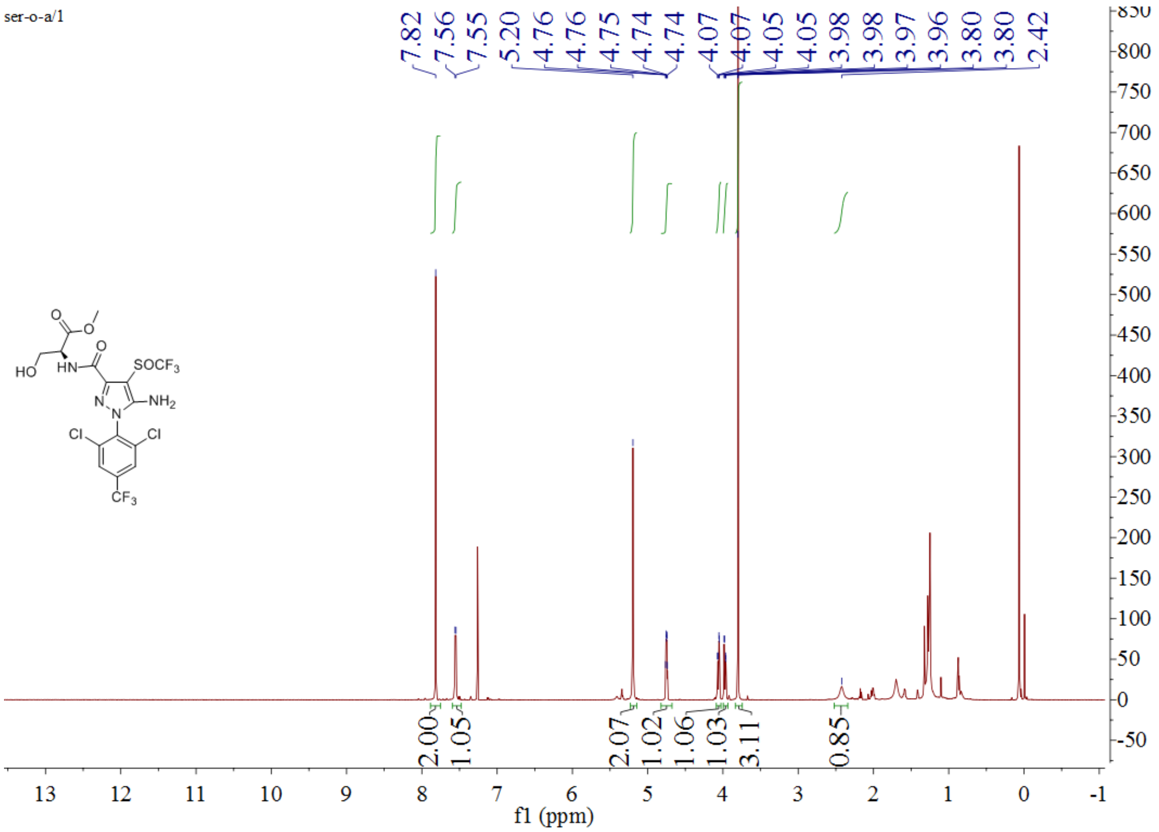

Figure S15 ^{1}H NMR spectra of **3g** in Chloroform-*d*

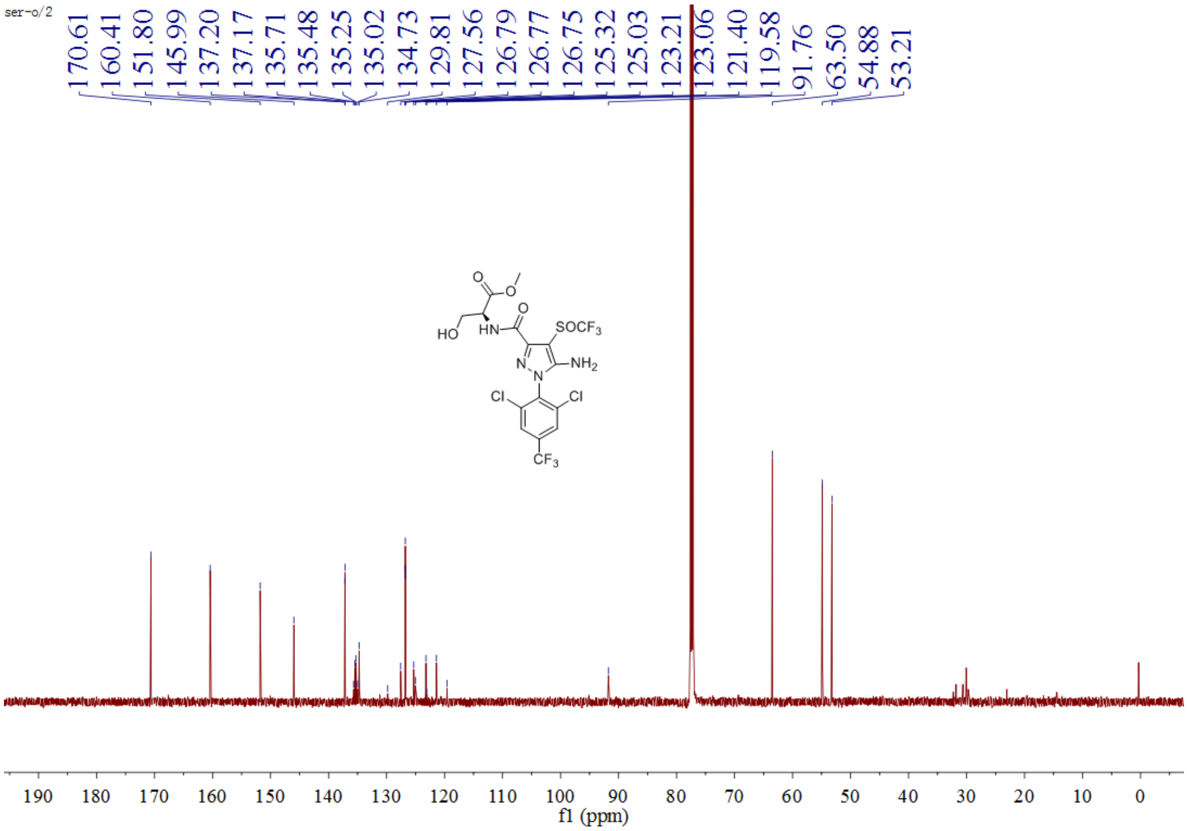

Figure S16 ^{13}C NMR spectra of **3g** in Chloroform-*d*

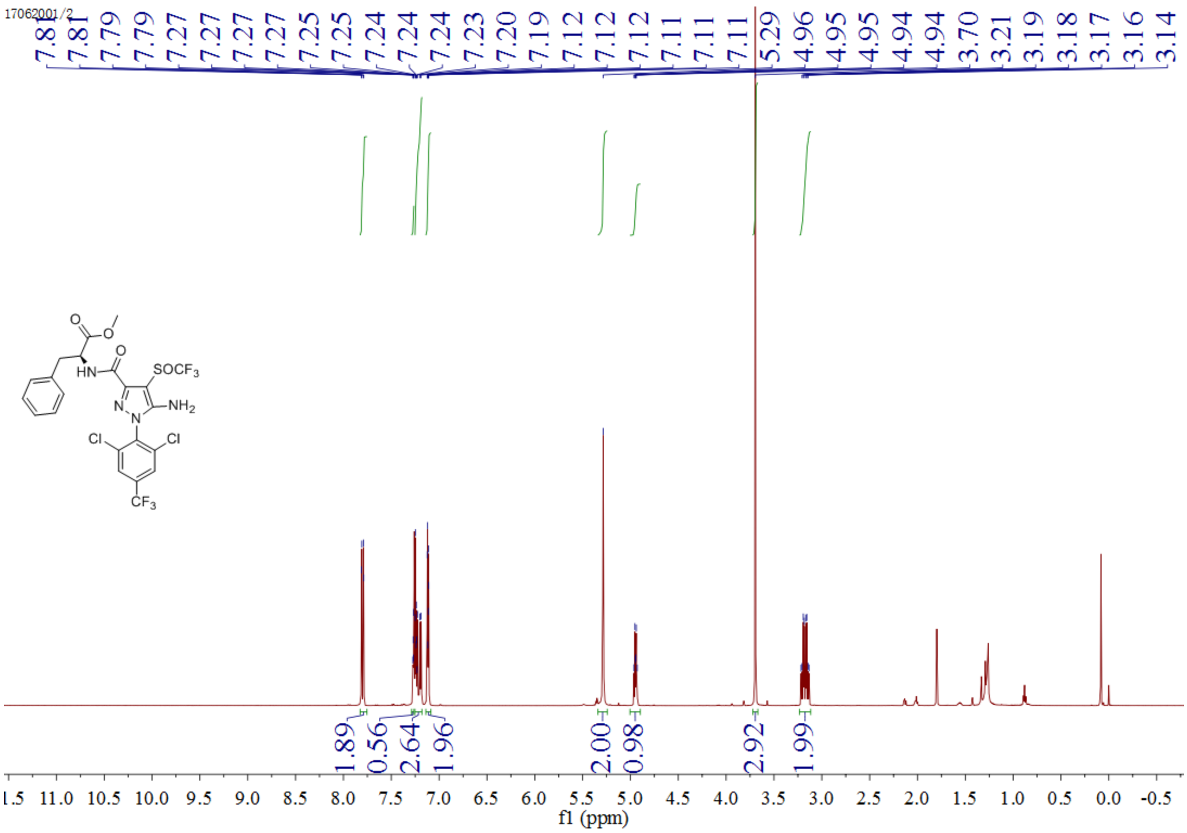

Figure S17 ^{1}H NMR spectra of **3h** in Chloroform-*d*

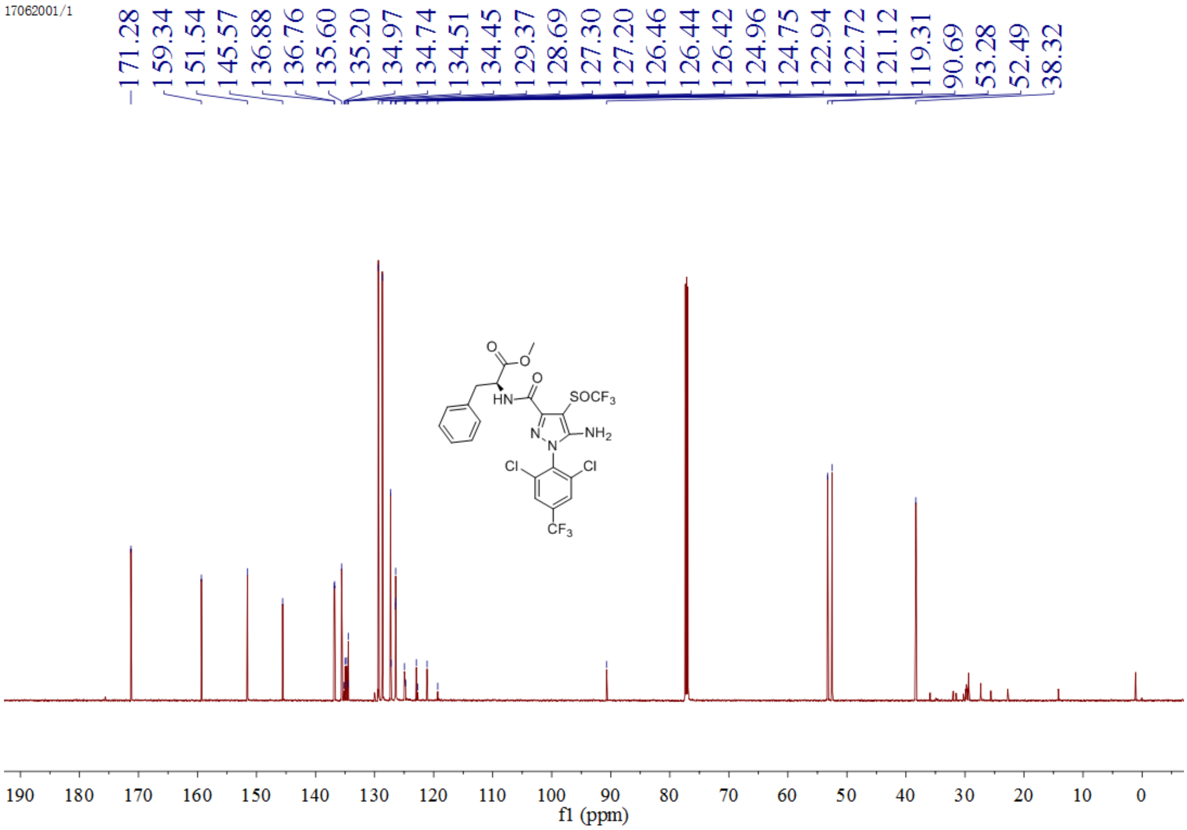

Figure S18 ^{13}C NMR spectra of **3h** in Chloroform-*d*

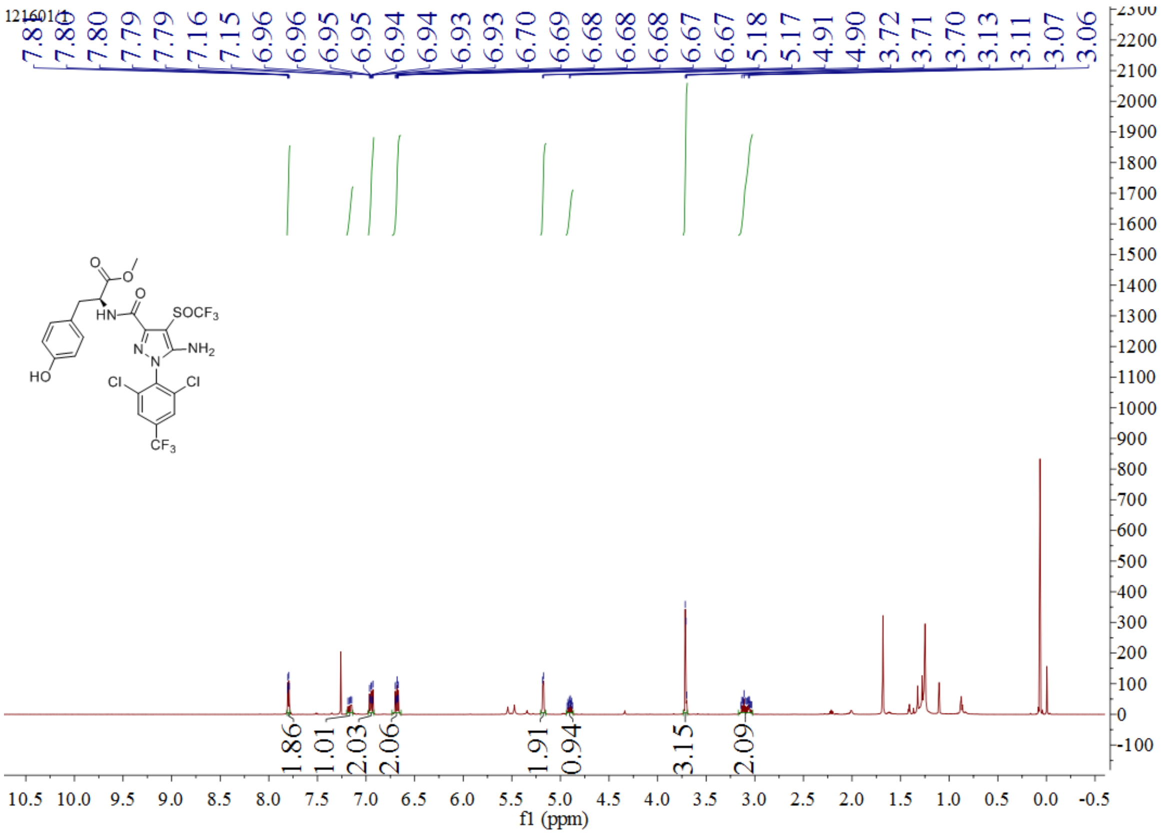

Figure S19 ^{1}H NMR spectra of **3i** in Chloroform-*d*

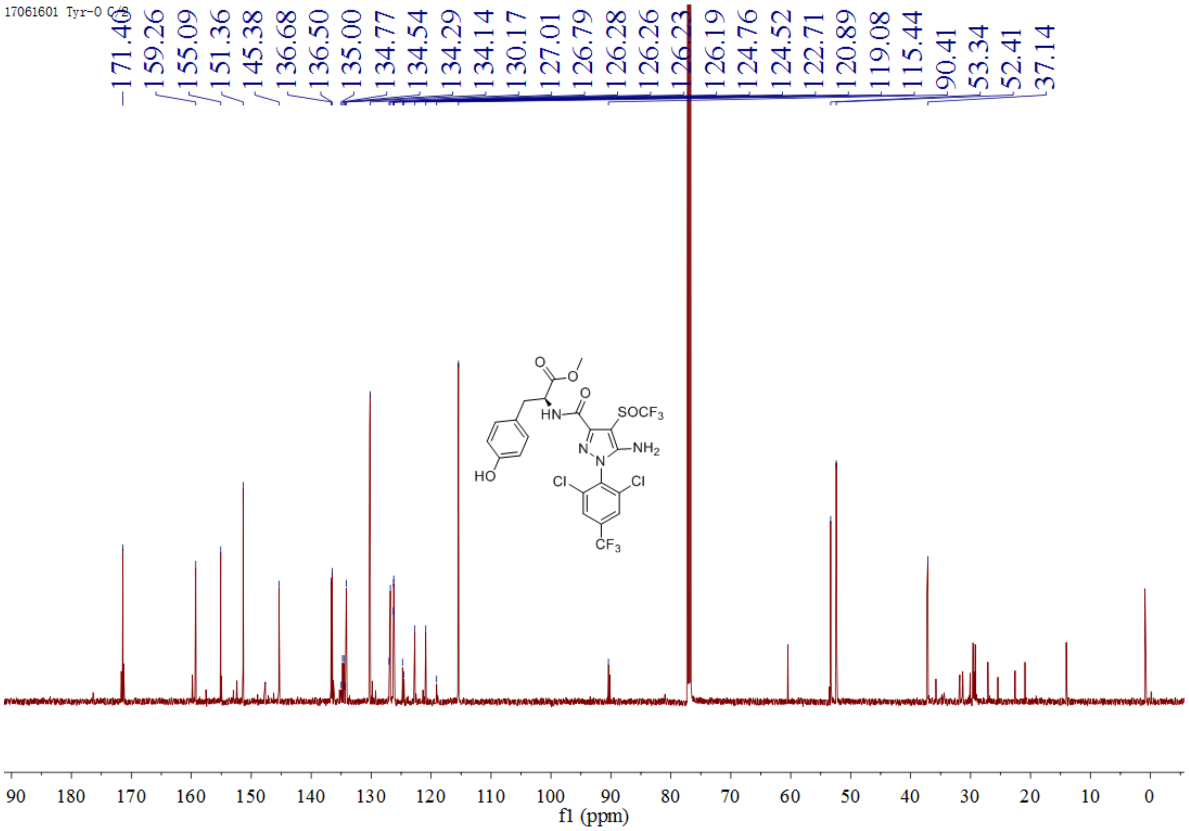

Figure S20 ^{13}C NMR spectra of **3i** in Chloroform-*d*

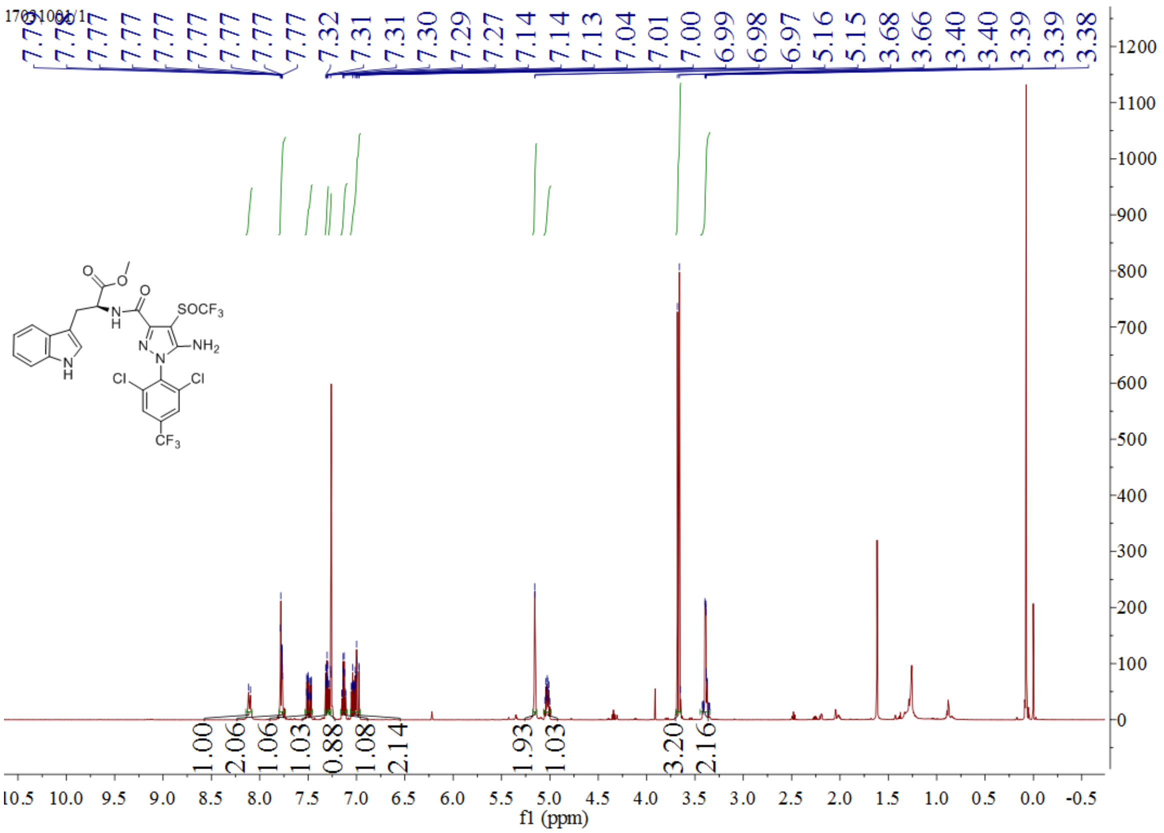

Figure S21 ^{1}H NMR spectra of **3j** in Chloroform-*d*

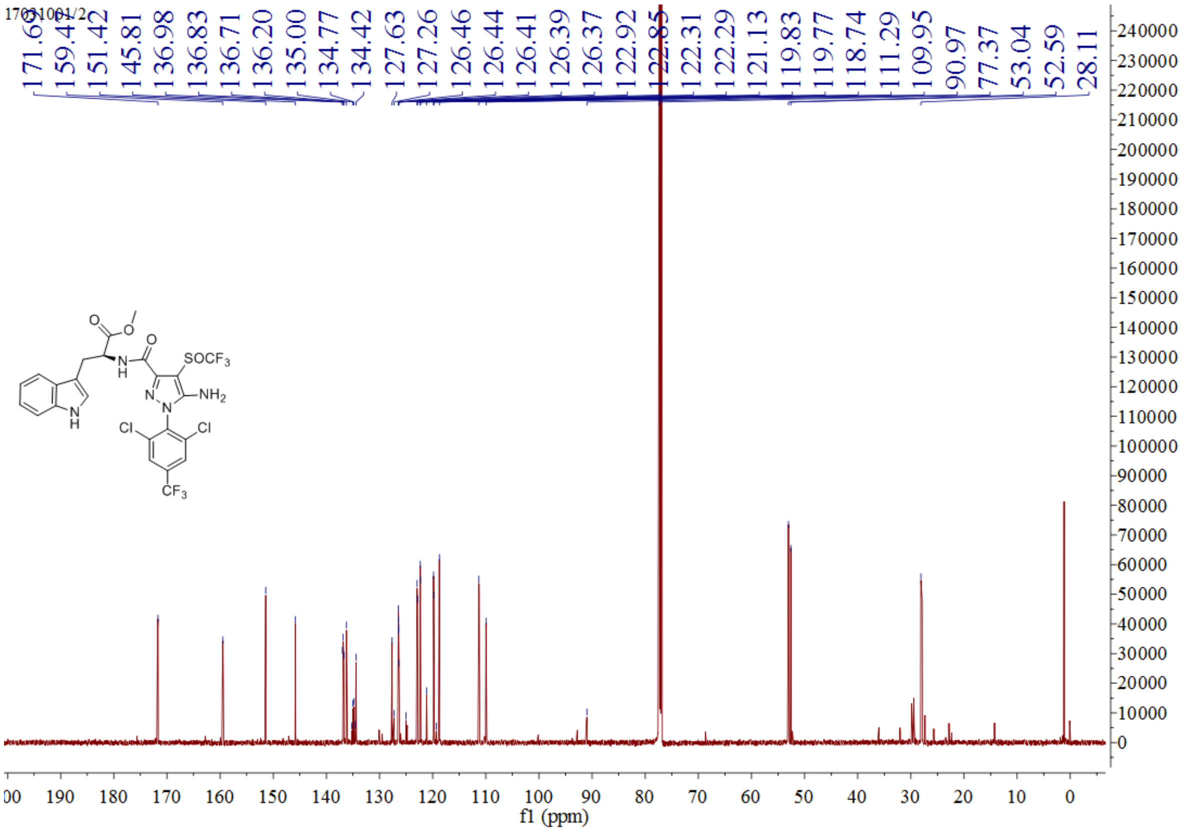

Figure S22 ^{13}C NMR spectra of **3j** in Chloroform-*d*

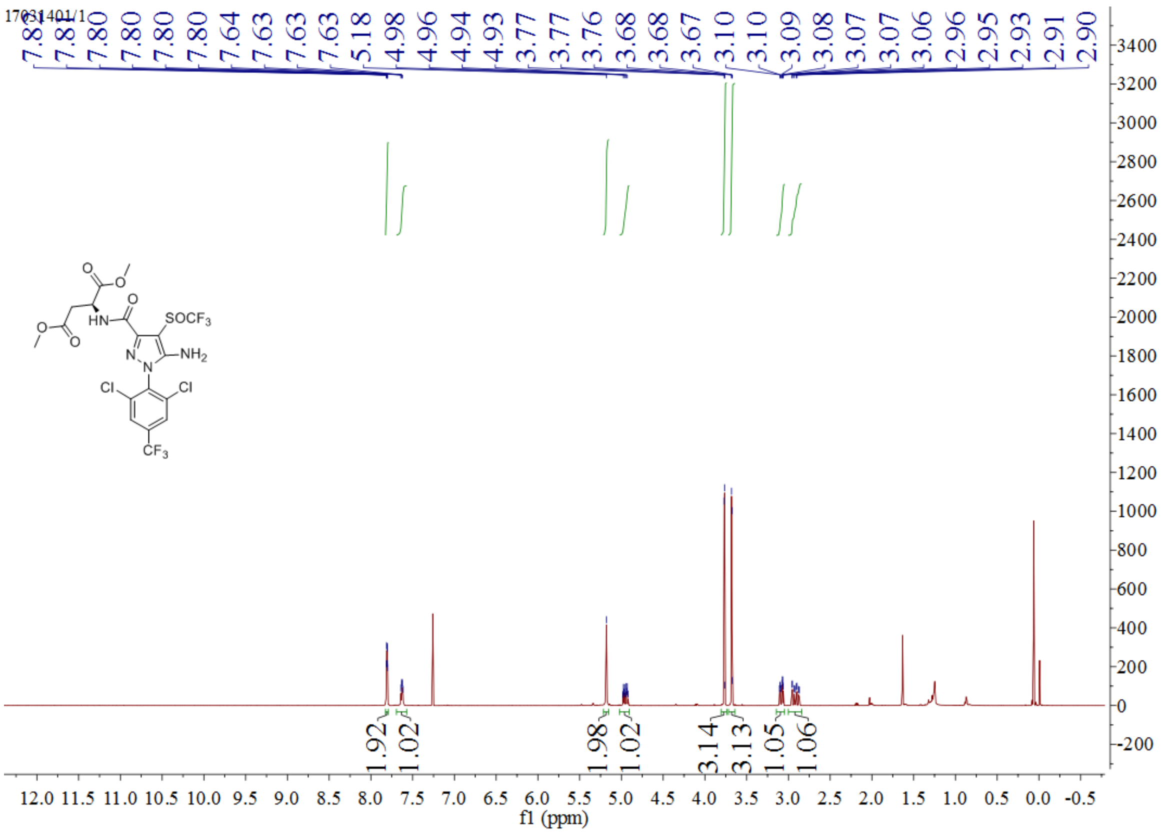

Figure S23 ^{1}H NMR spectra of **3k** in Chloroform-*d*

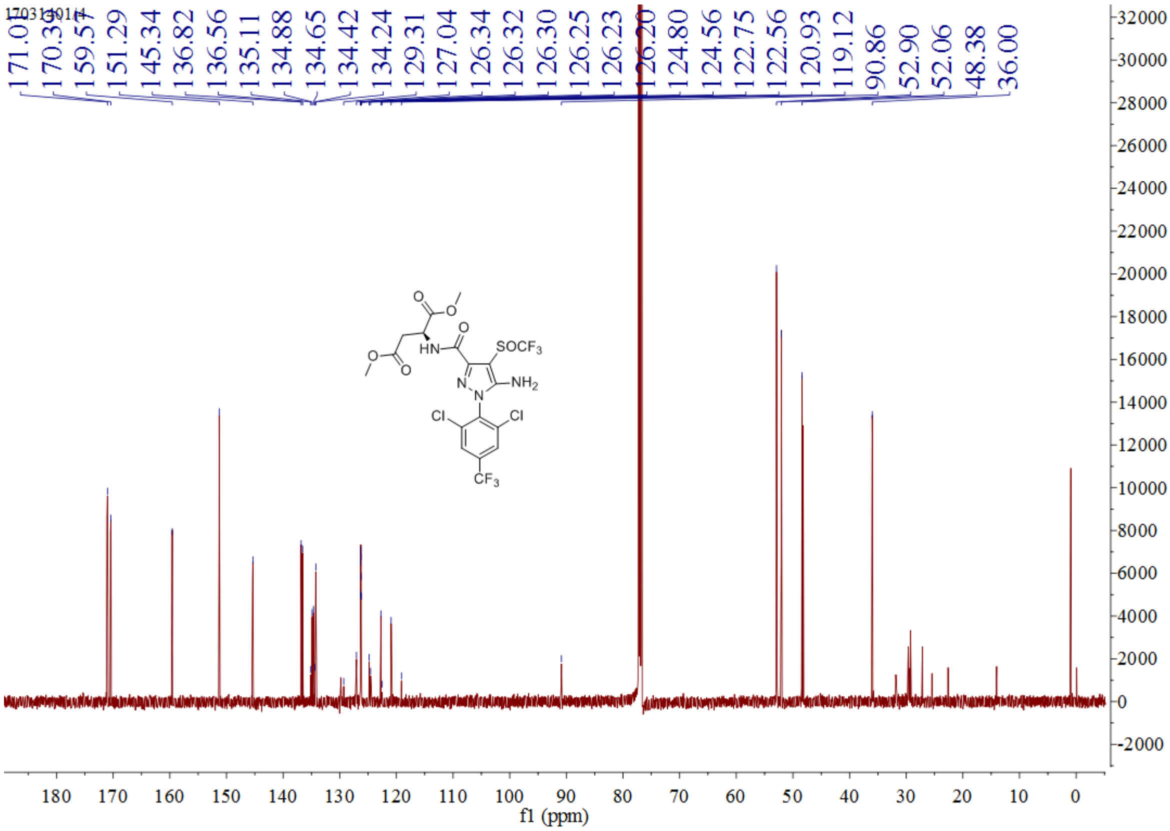

Figure S24 ^{13}C NMR spectra of **3k** in Chloroform-*d*

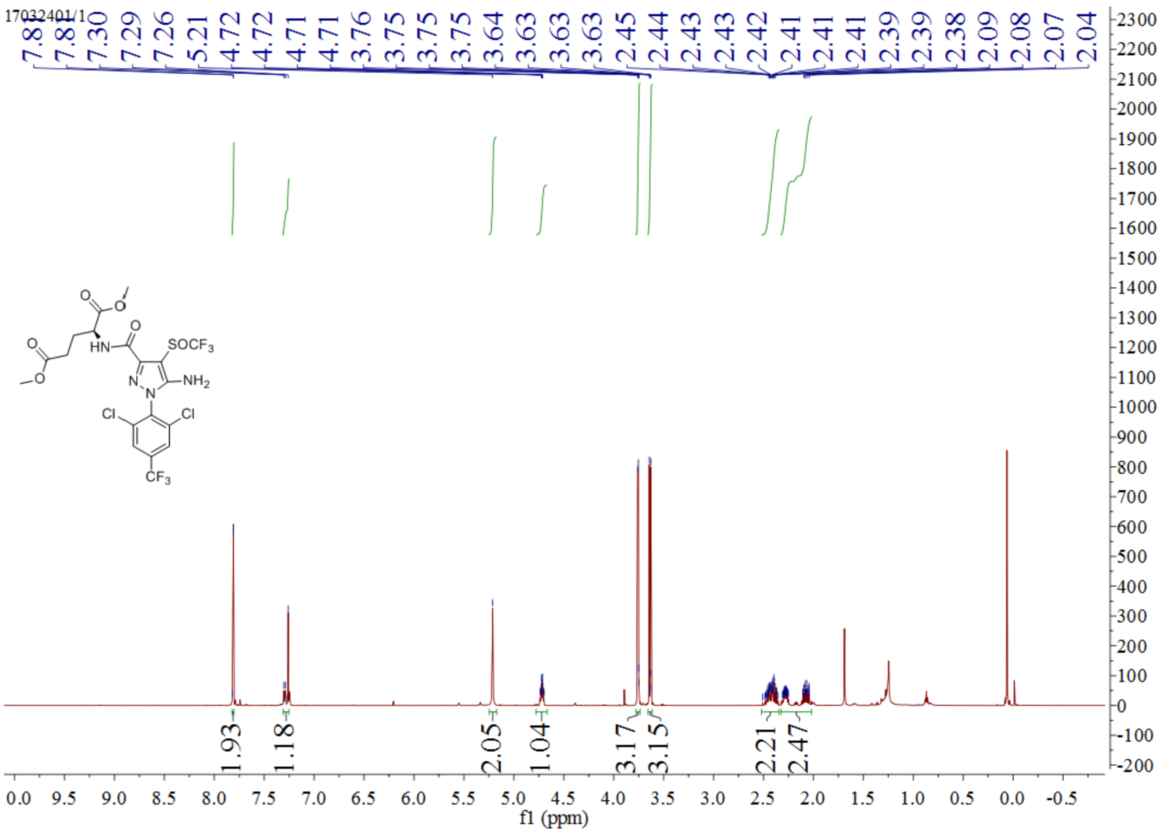

Figure S25 ^{1}H NMR spectra of **3l** in Chloroform-*d*

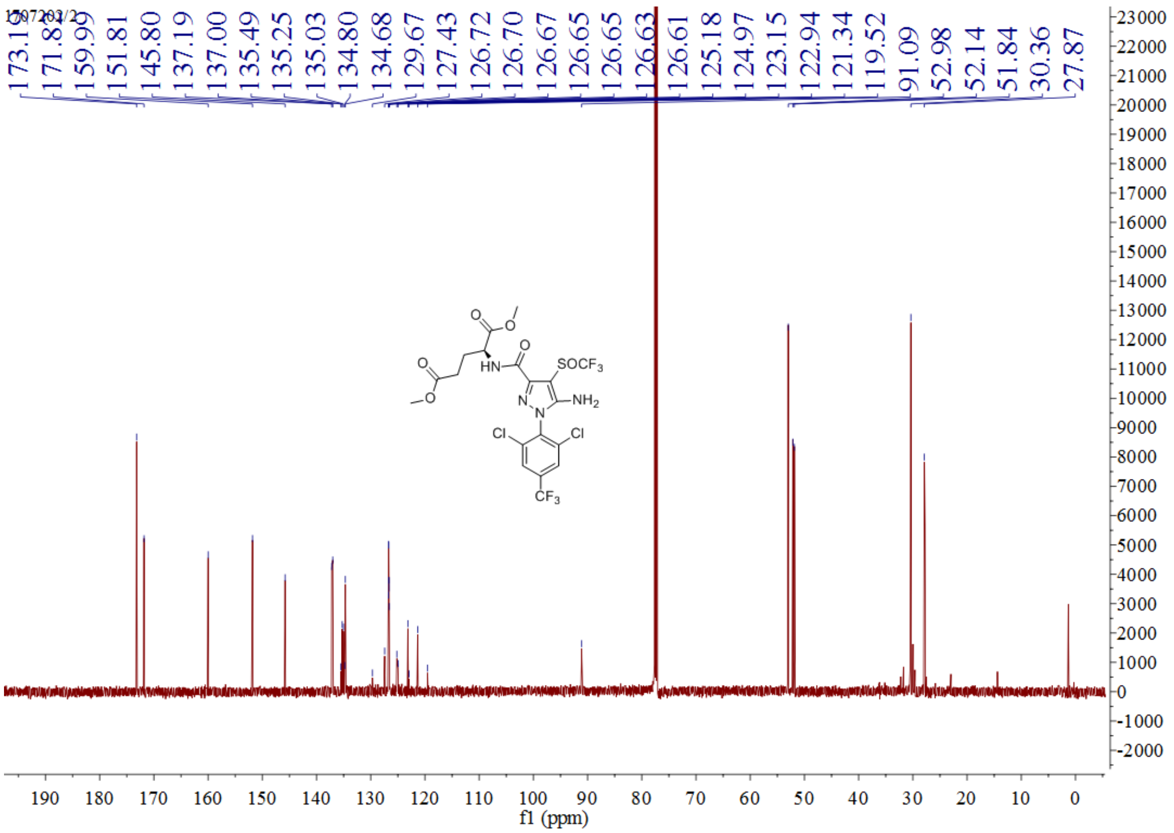

Figure S26 ^{13}C NMR spectra of **3l** in Chloroform-*d*

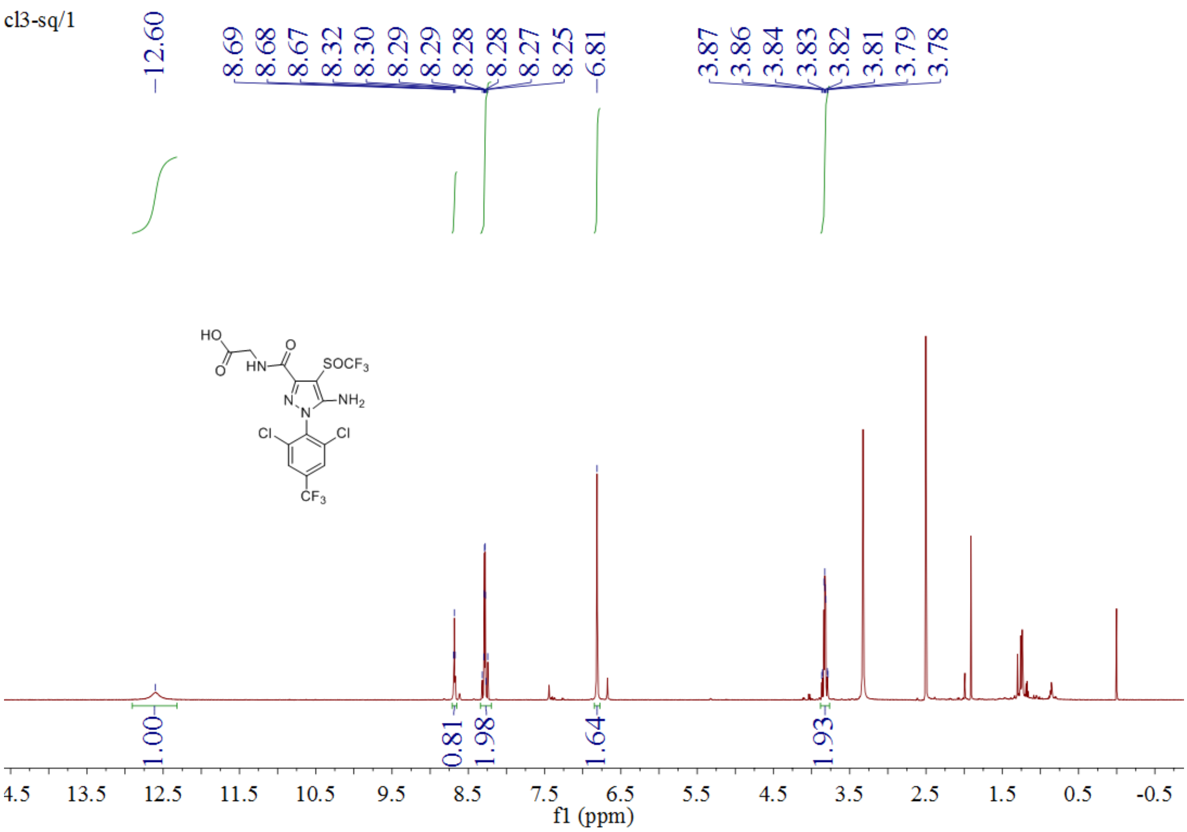

Figure S27 ¹H NMR spectra of **4a** in Chloroform-*d*

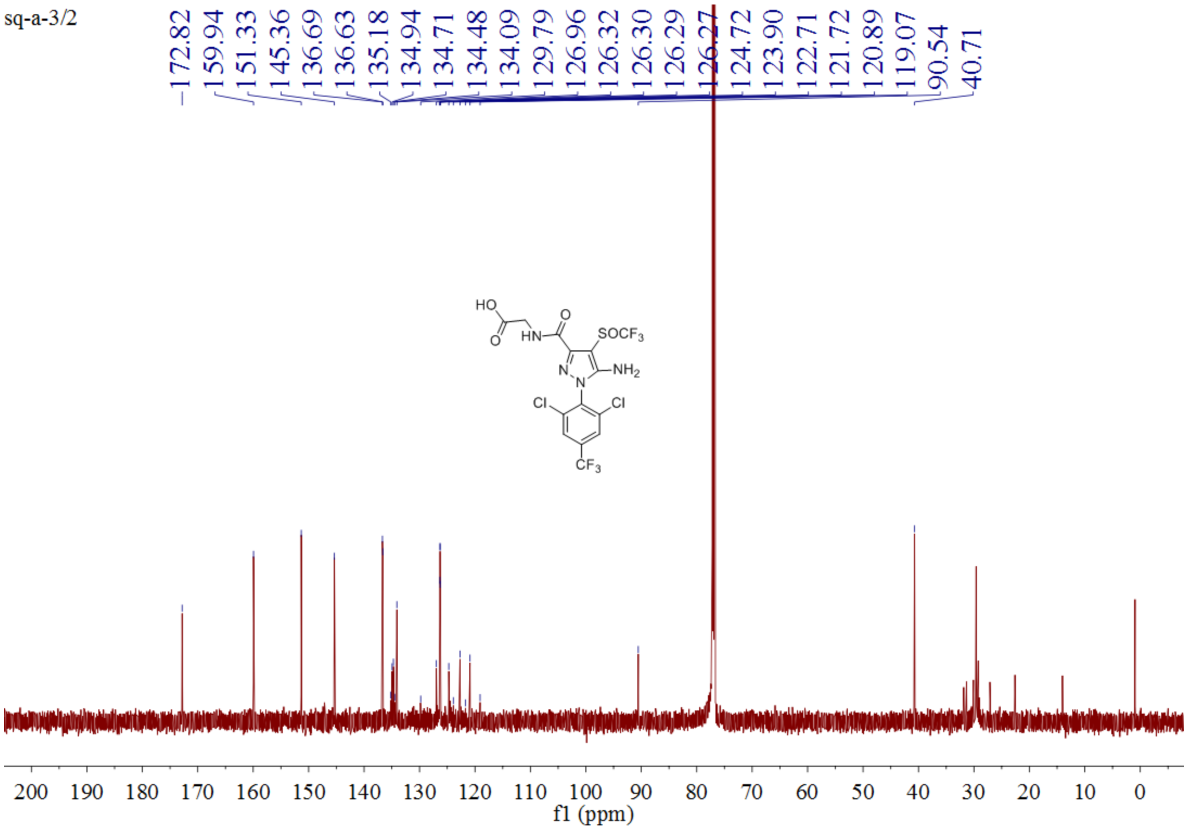

Figure S28 ¹³C NMR spectra of **4a** in Chloroform-*d*

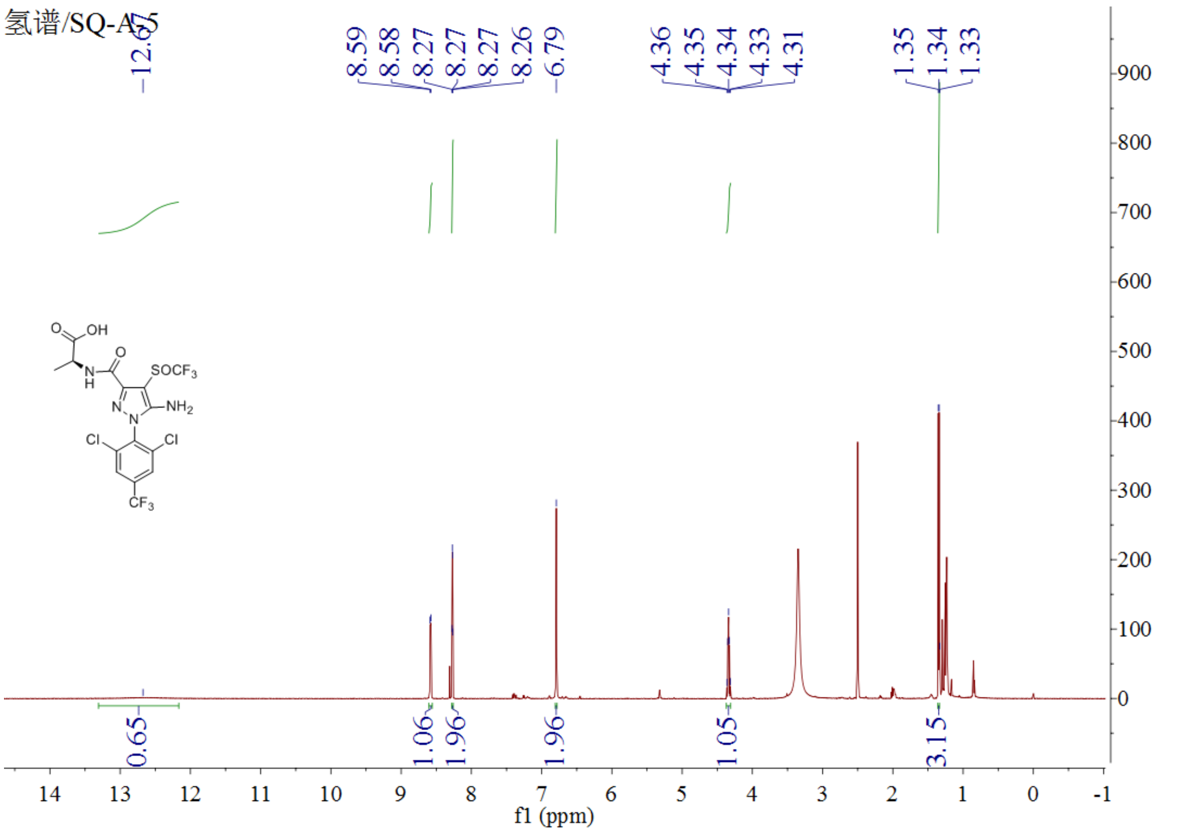

Figure S29 ^{1}H NMR spectra of **4b** in DMSO-d_6

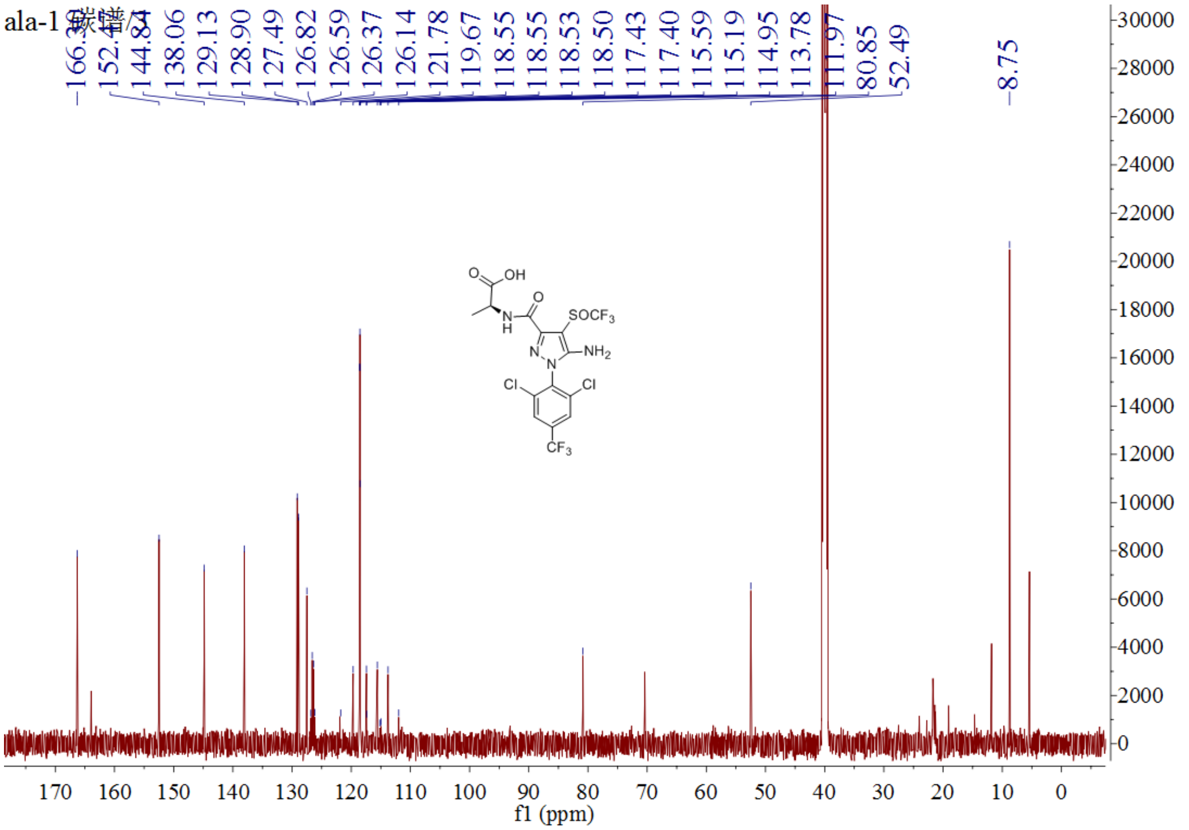

Figure S30 ^{13}C NMR spectra of **4b** in DMSO-d_6

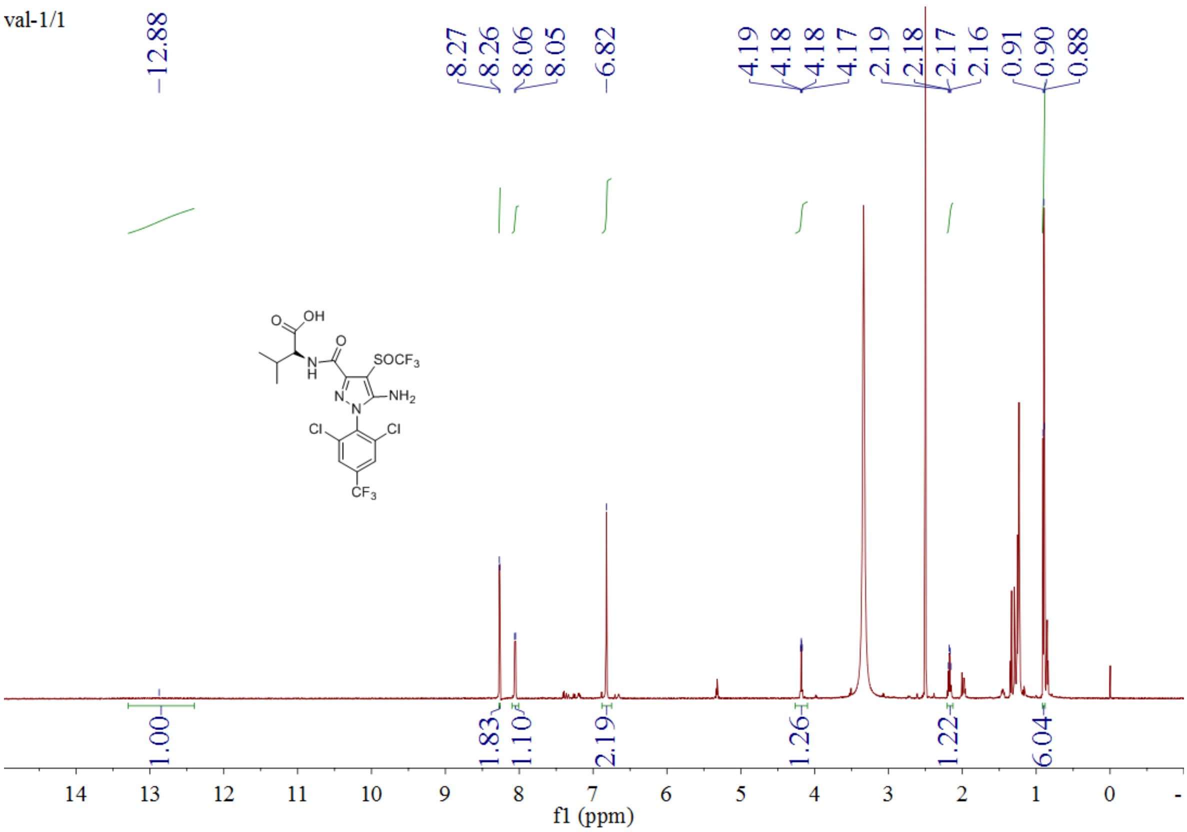

Figure S31 ^{1}H NMR spectra of **4c** in DMSO-*d*6

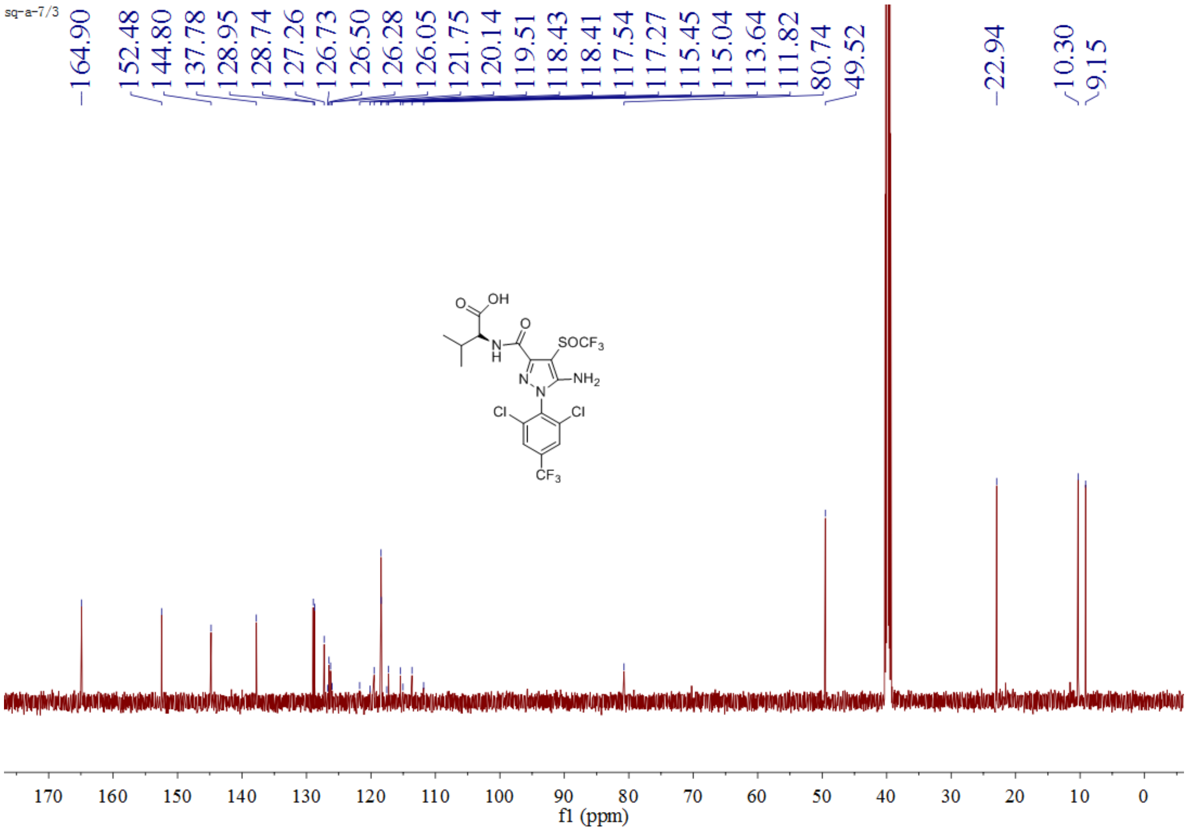

Figure S32 ^{13}C NMR spectra of **4c** in DMSO-*d*6

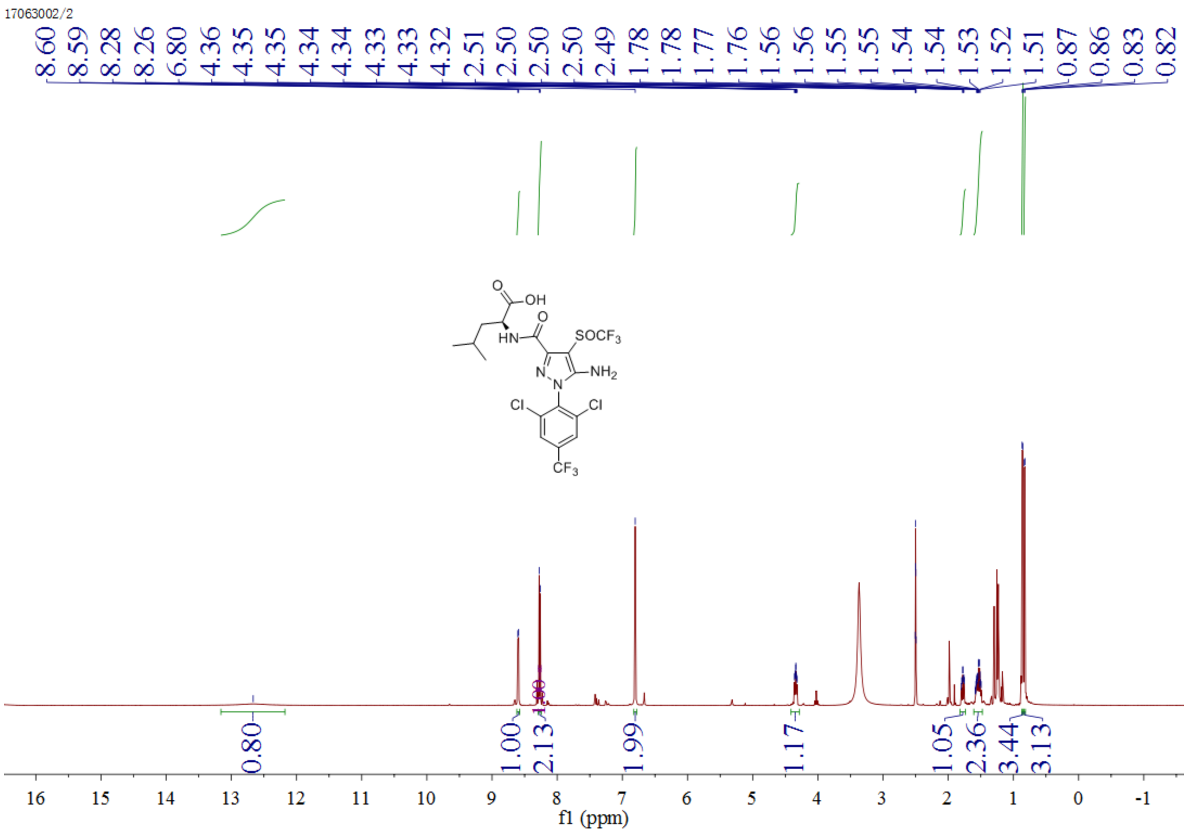

Figure S33 ^{1}H NMR spectra of **4d** in DMSO-d_6

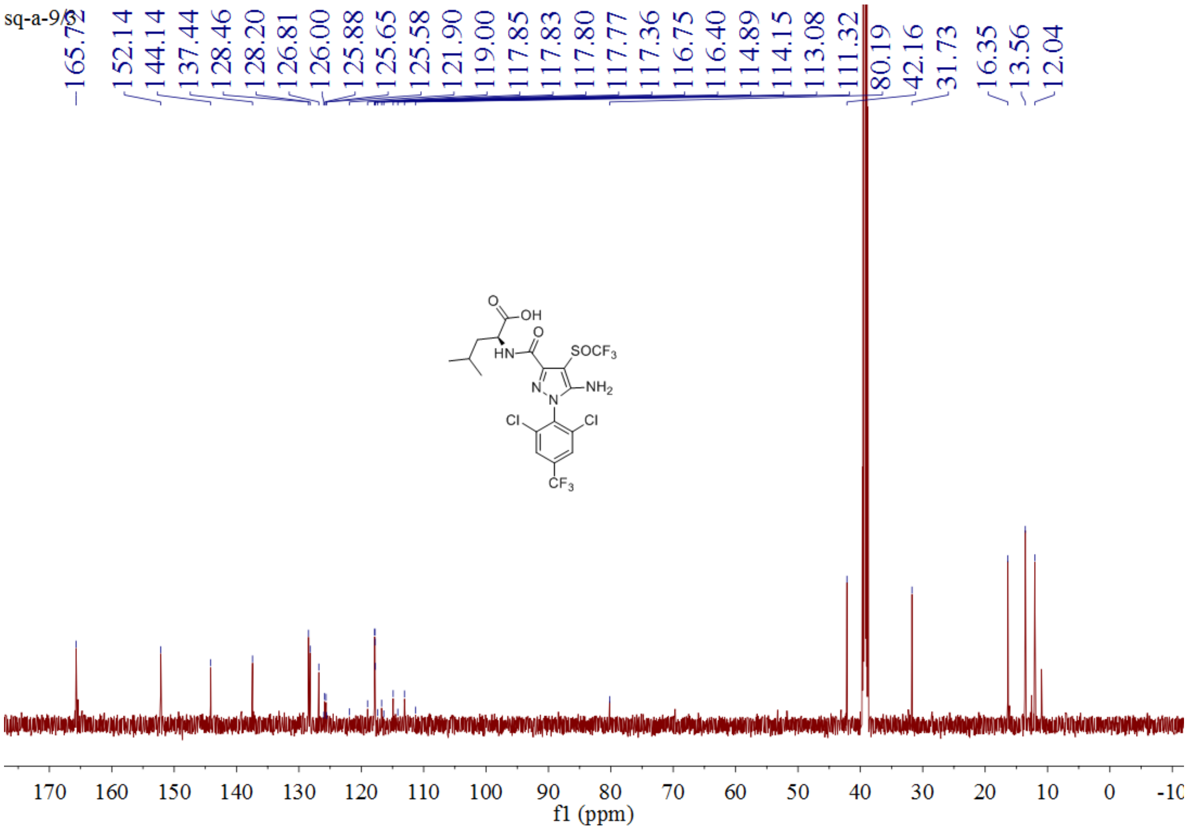

Figure S34 ^{13}C NMR spectra of **4d** in DMSO-d_6

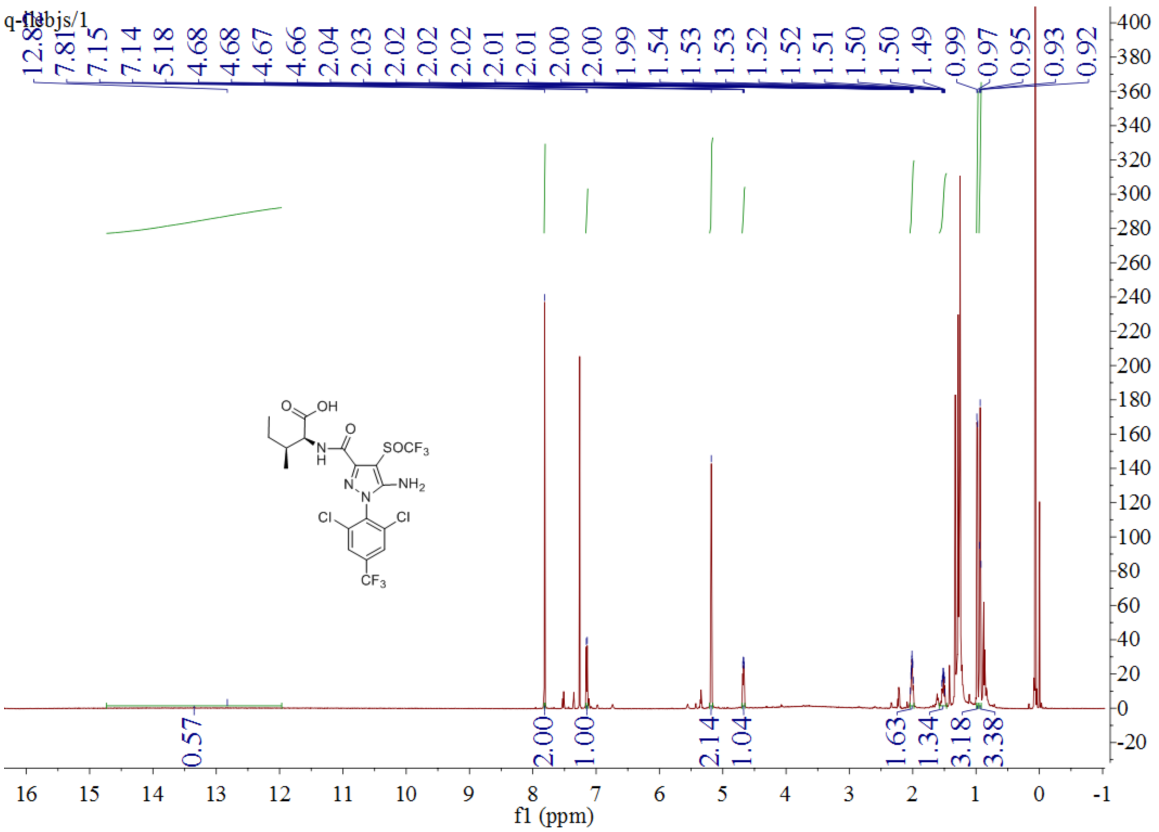

Figure S35 ^{1}H NMR spectra of **4e** in Chloroform-*d*

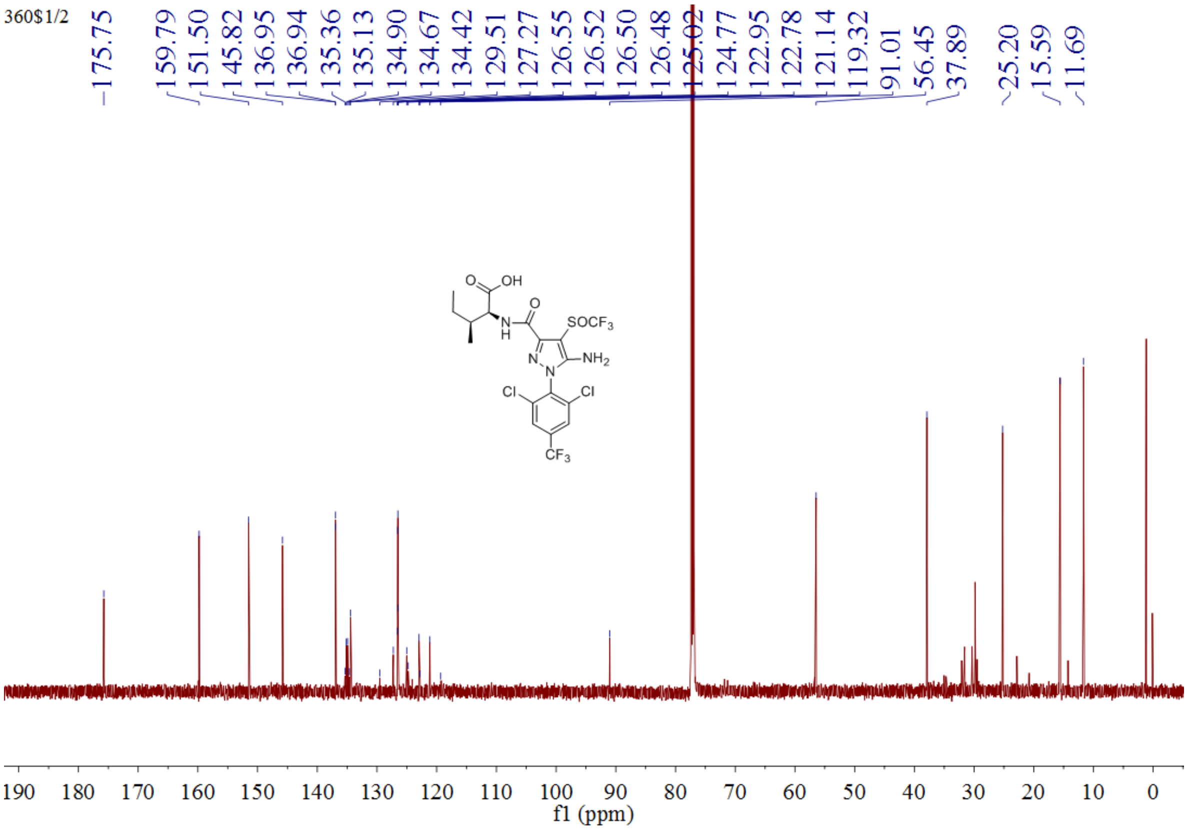

Figure S36 ^{13}C NMR spectra of **4e** in Chloroform-*d*

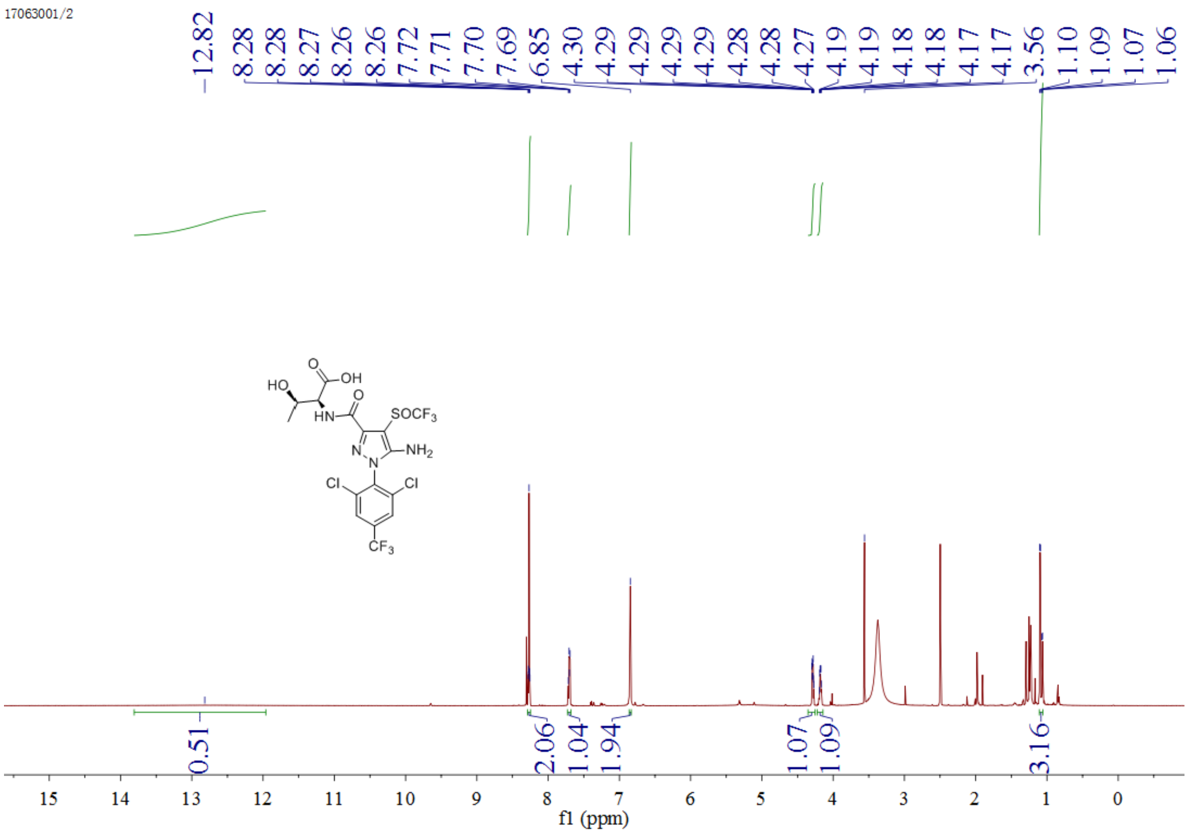

Figure S37 ^{1}H NMR spectra of **4f** in DMSO-d_6

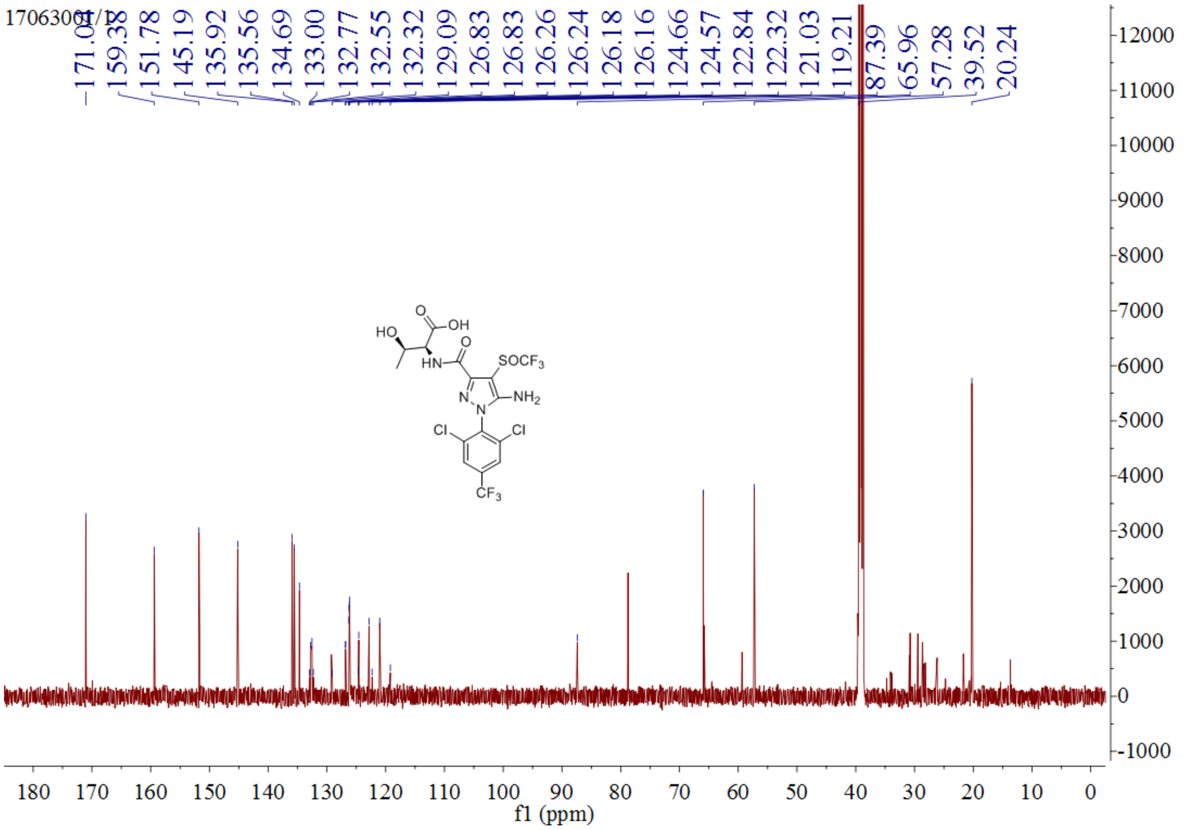

Figure S38 ^{13}C NMR spectra of **4f** in DMSO-d_6

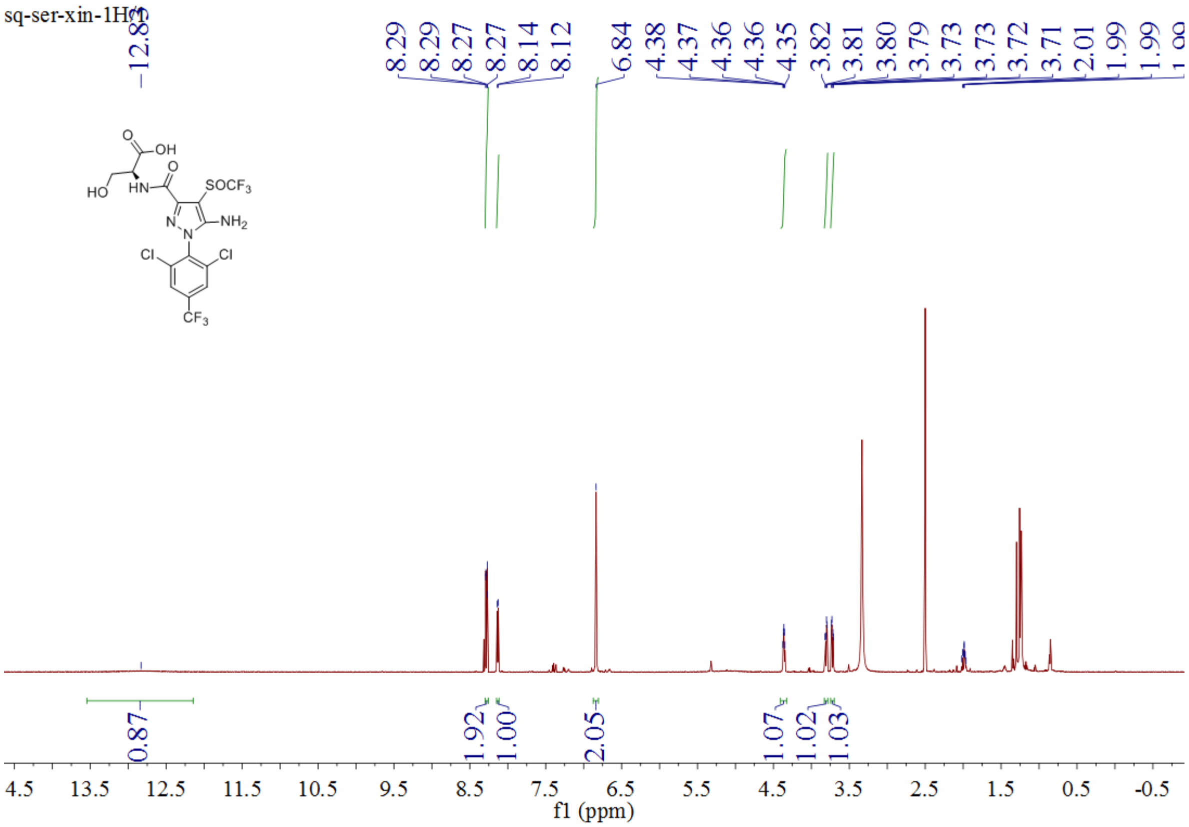

Figure S39 ^{1}H NMR spectra of **4g** in DMSO-d_6

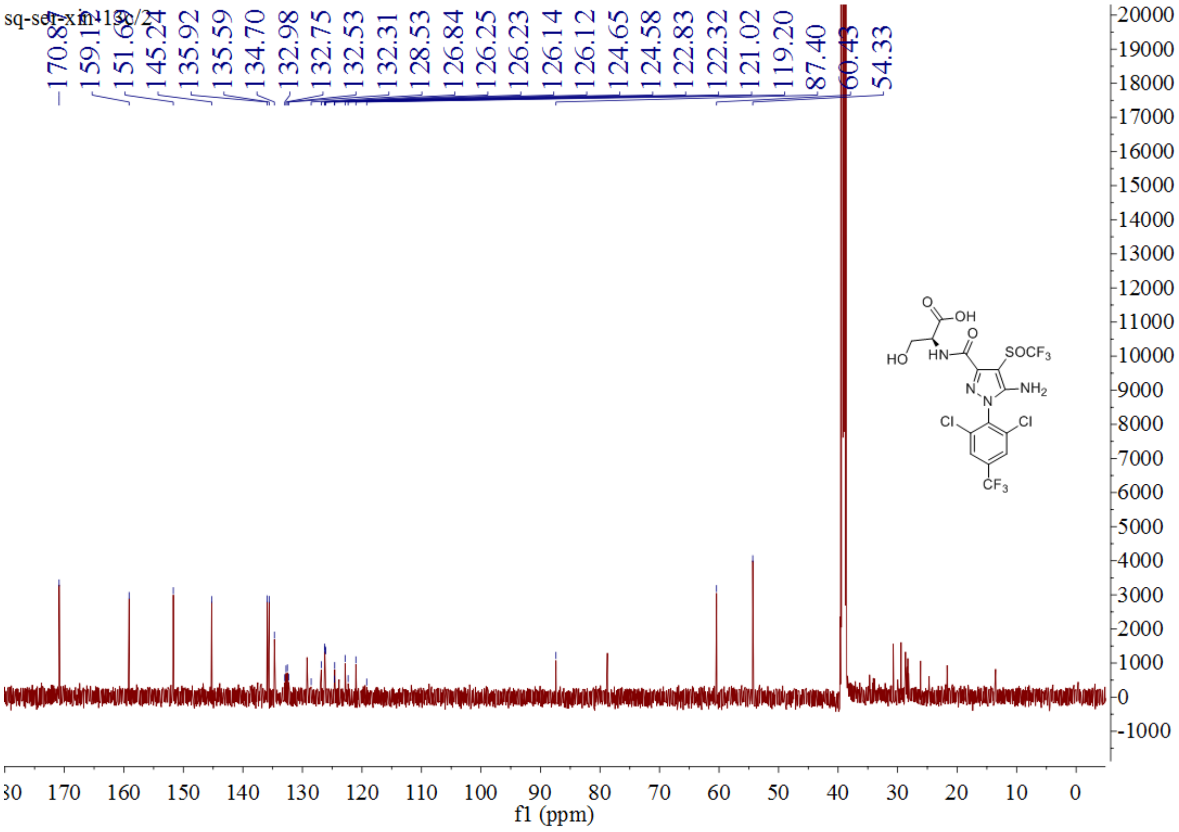

Figure S40 ^{13}C NMR spectra of **4g** in DMSO-d_6

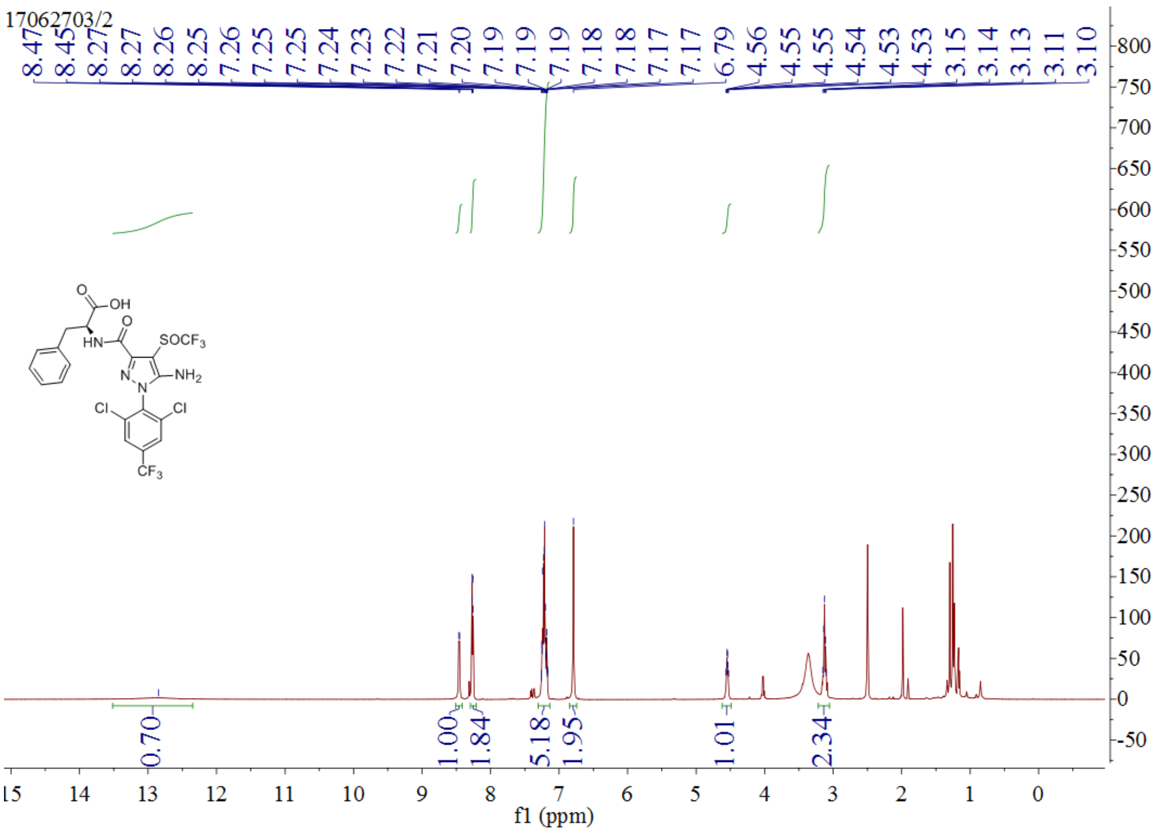

Figure S41 ^{1}H NMR spectra of **4h** in DMSO-d_6

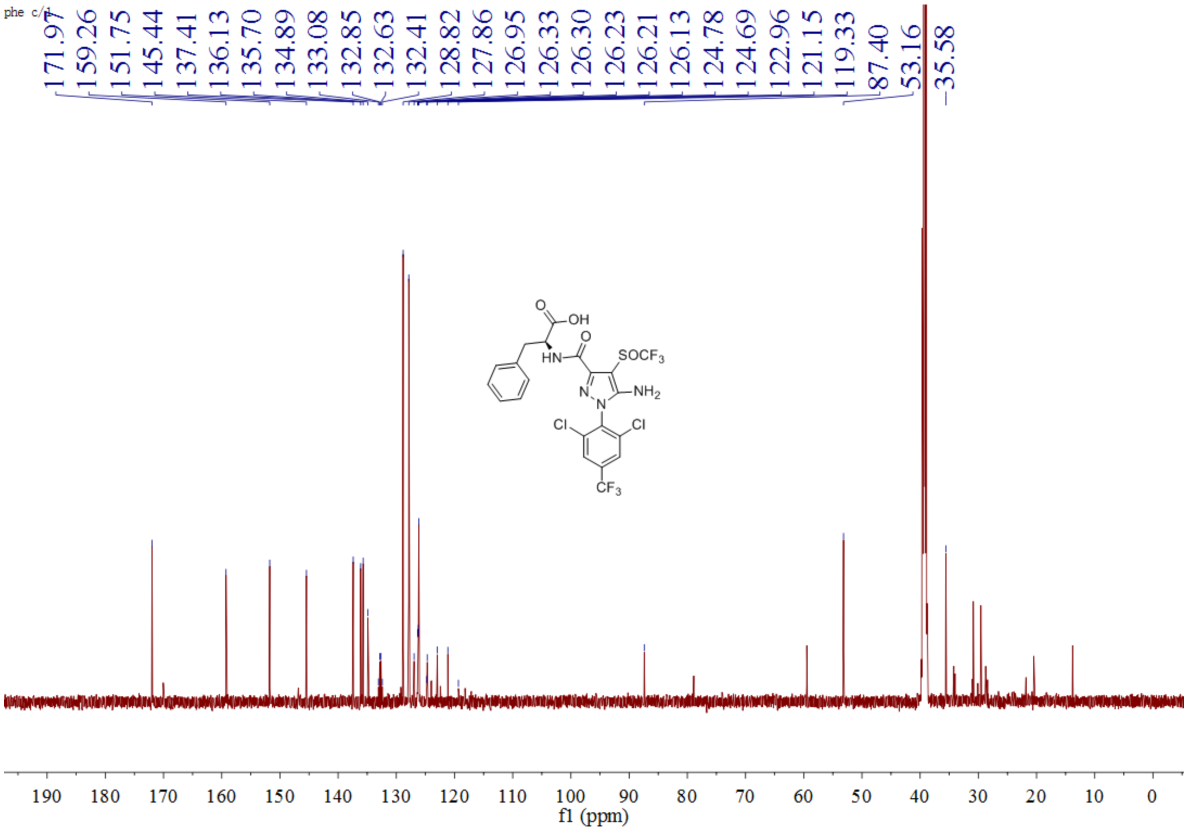

Figure S42 ^{13}C NMR spectra of **4h** in DMSO-d_6

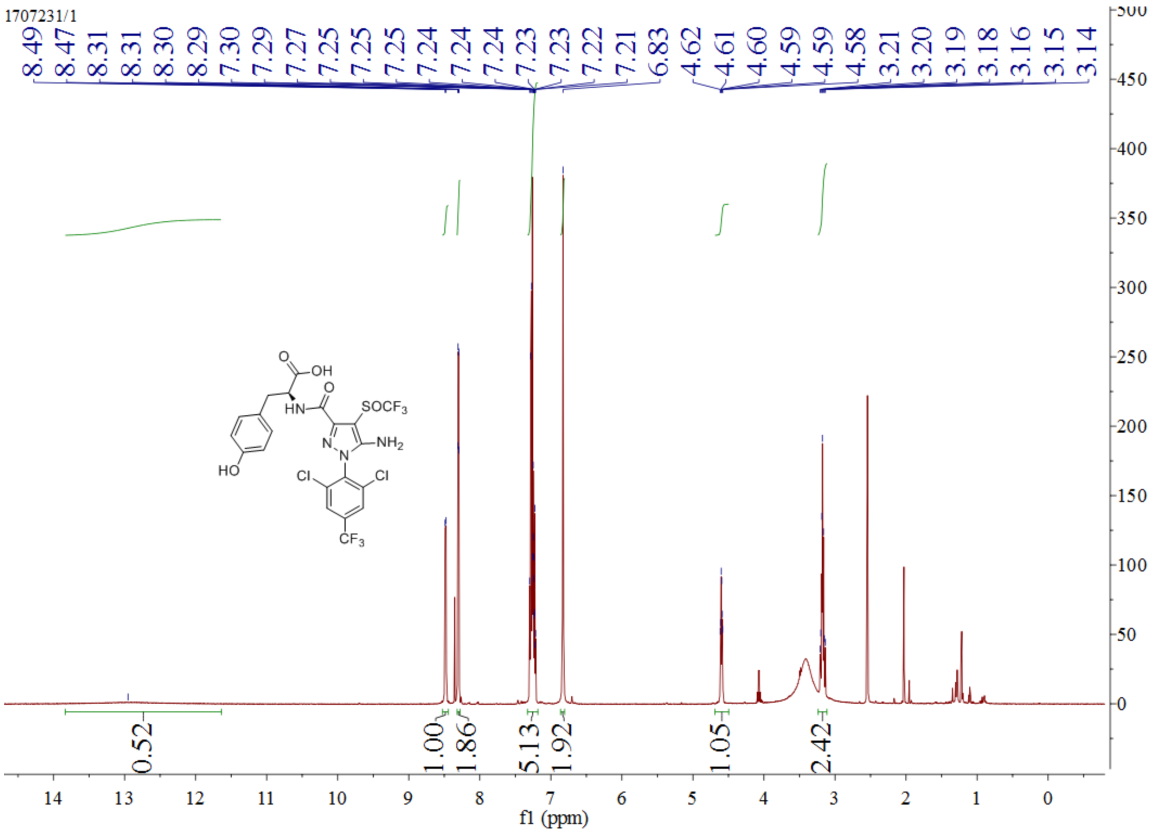

Figure S43 ^{1}H NMR spectra of **4i** in DMSO-d_6

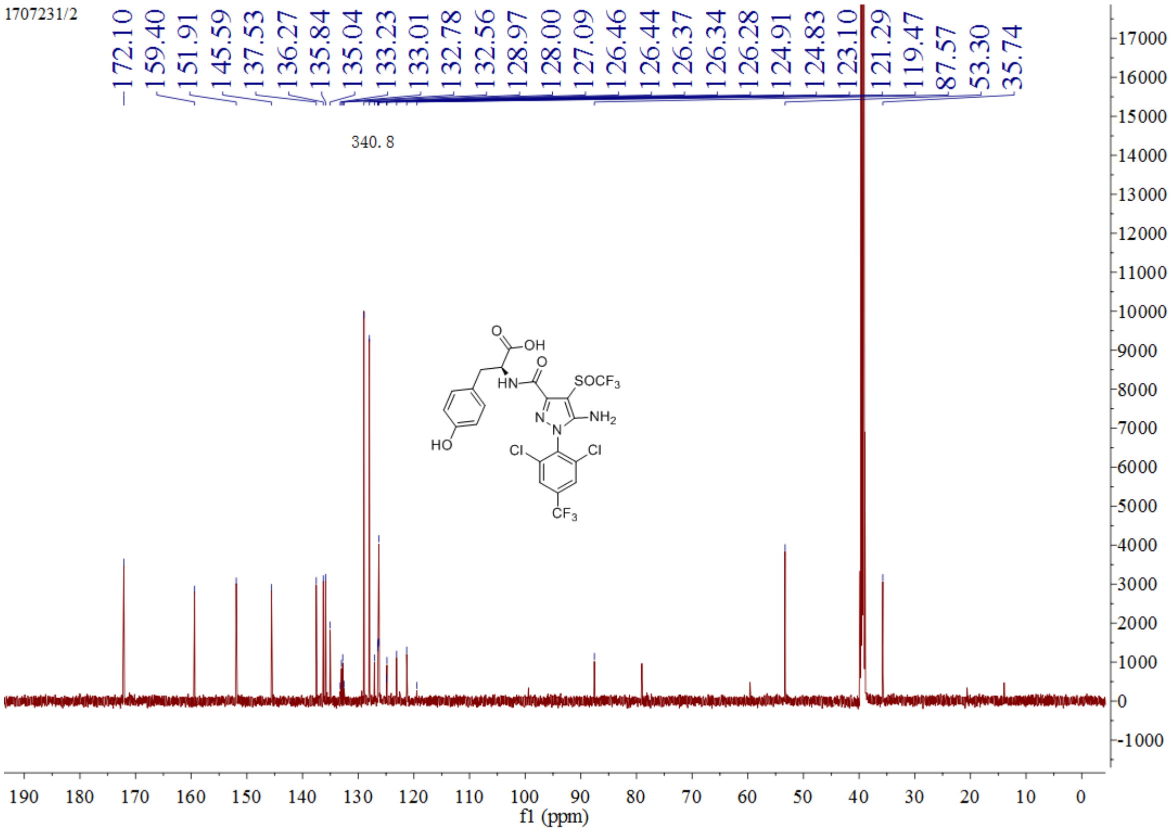

Figure S44 ^{13}C NMR spectra of **4i** in DMSO-d_6

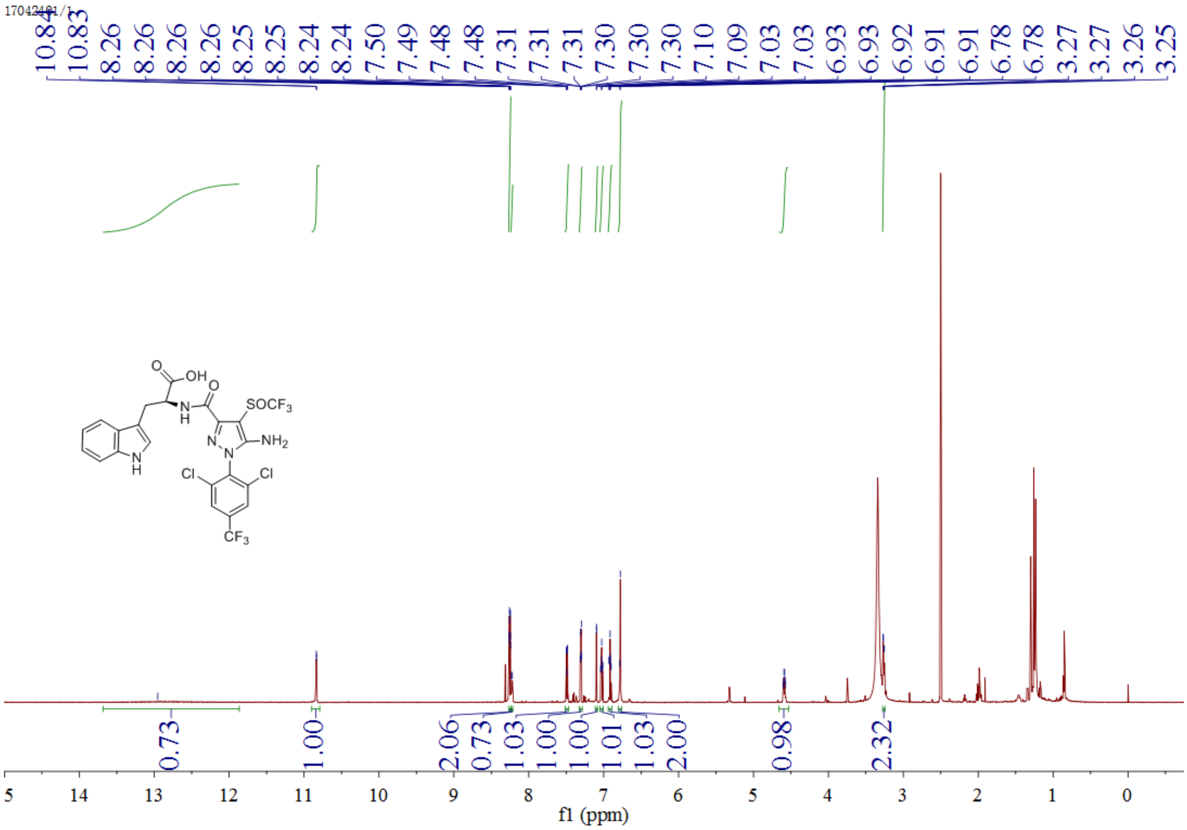

Figure S45 ^{1}H NMR spectra of **4j** in Chloroform-*d*

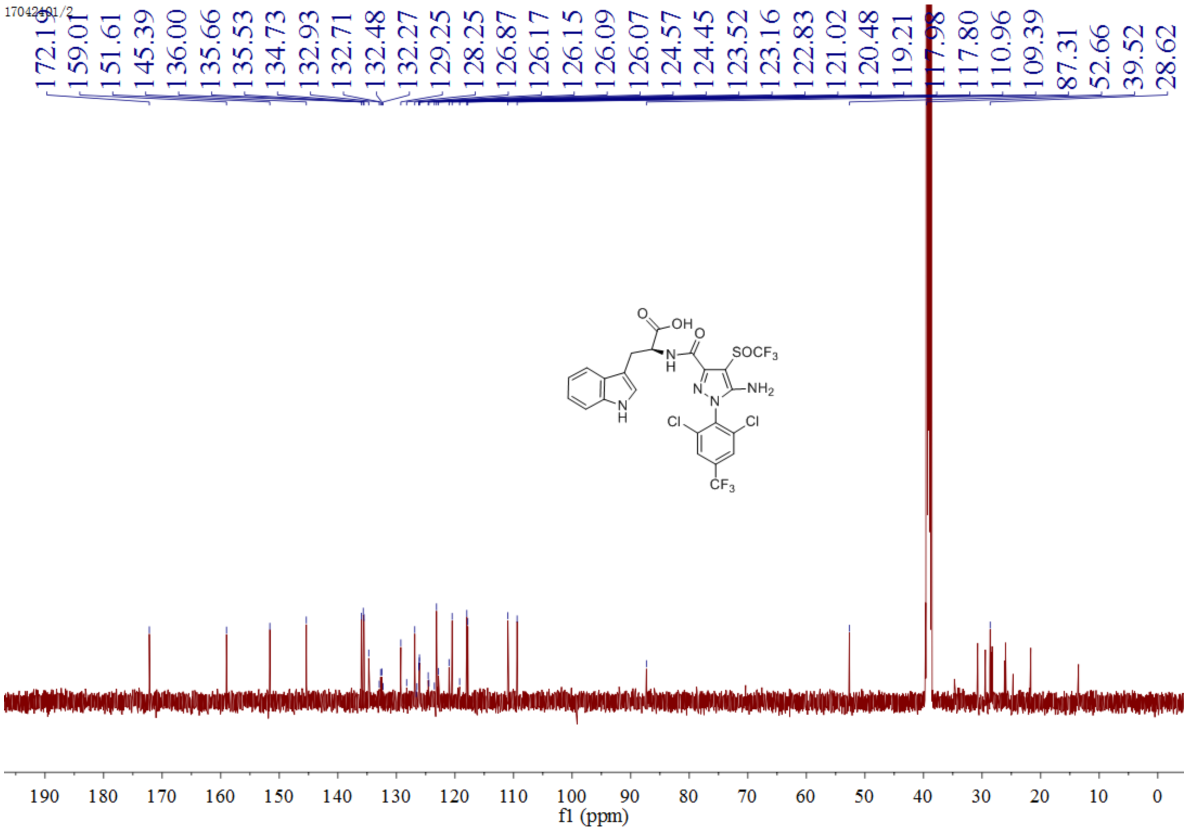

Figure S46 ^{13}C NMR spectra of **4j** in DMSO-d_6

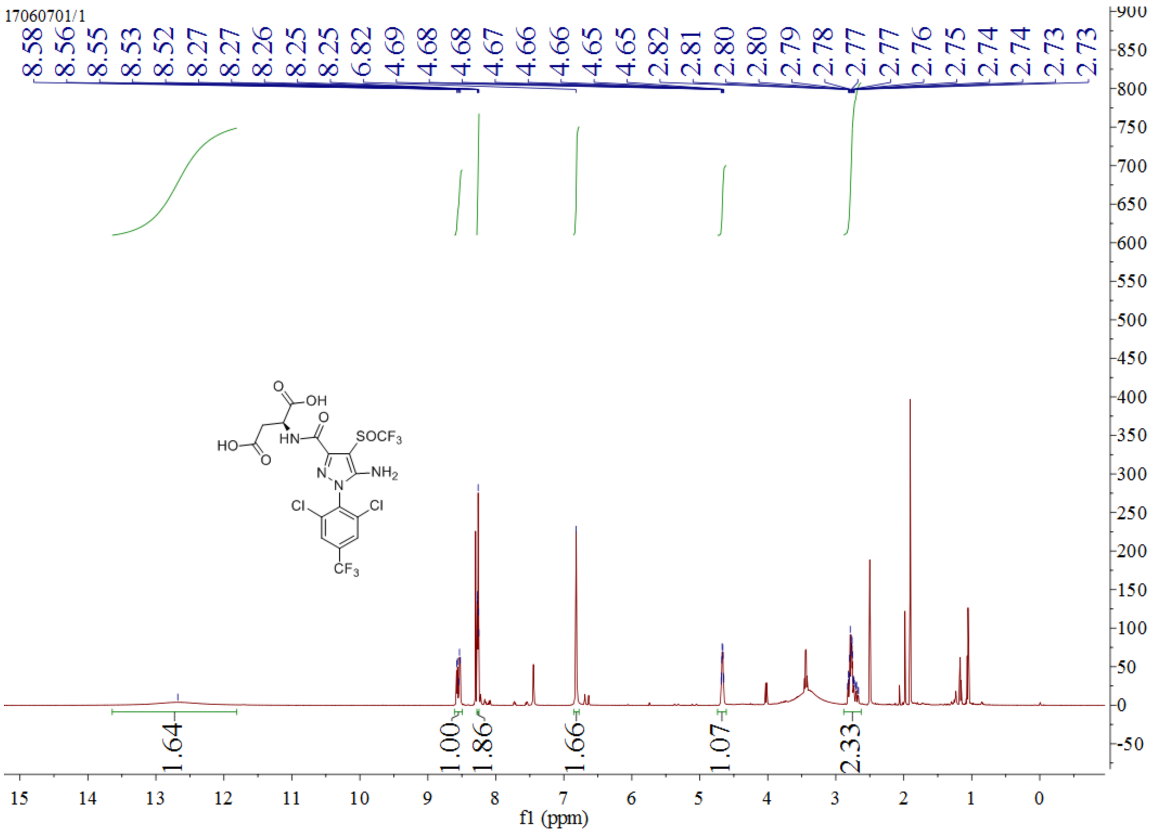

Figure S47 ^{1}H NMR spectra of **4k** in DMSO-d_6

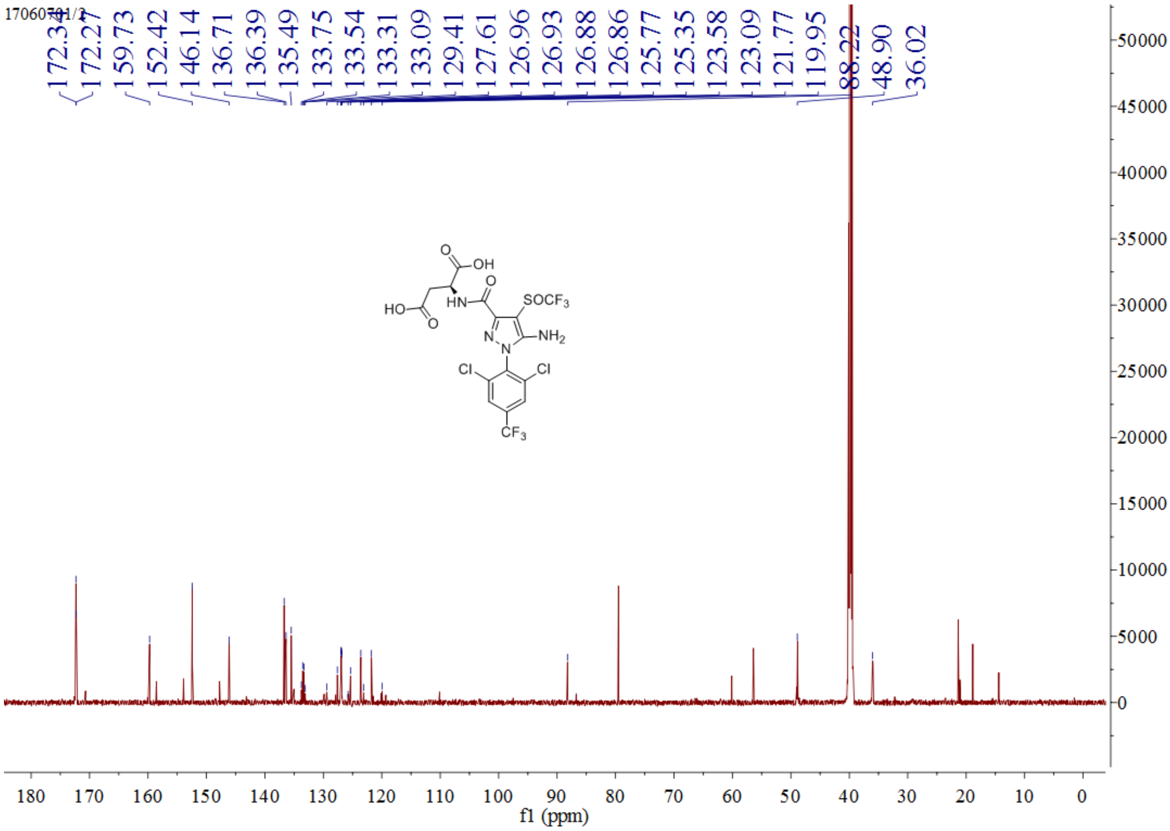

Figure S48 ^{13}C NMR spectra of **4k** in DMSO-d_6

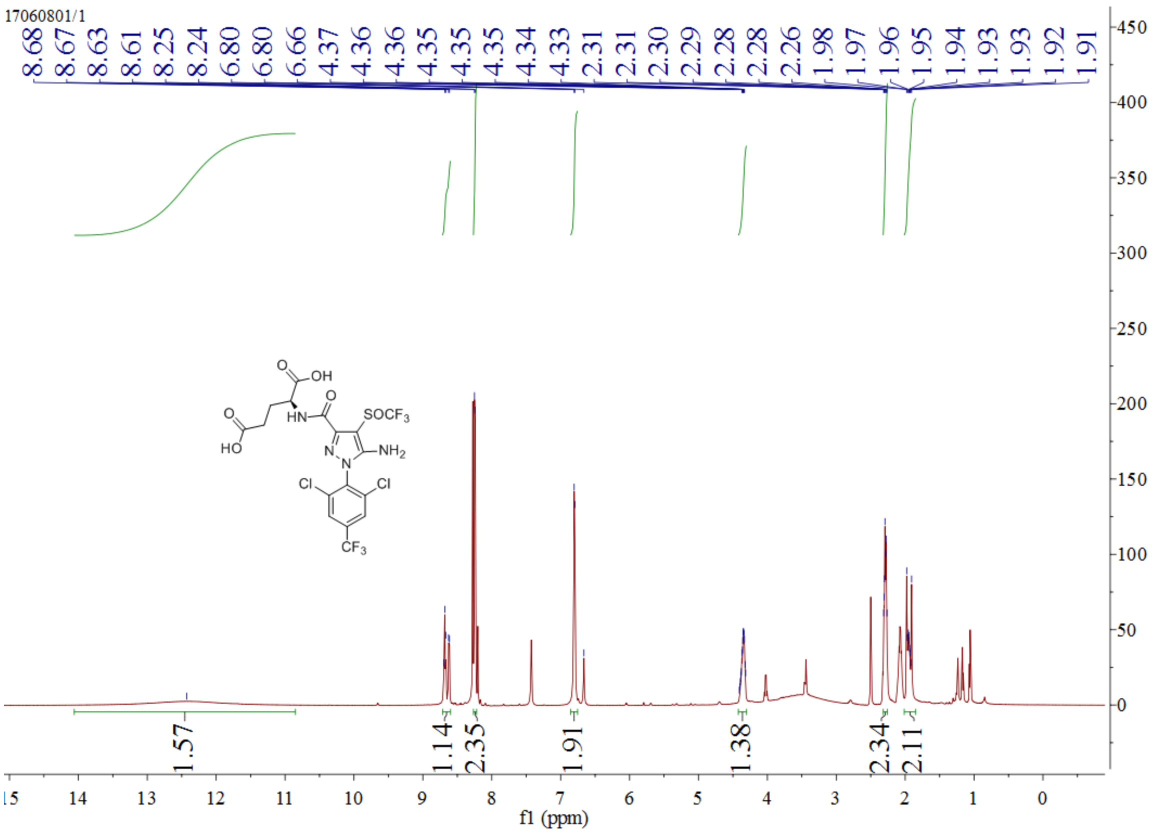

Figure S49 ^{1}H NMR spectra of **4l** in DMSO-d_6

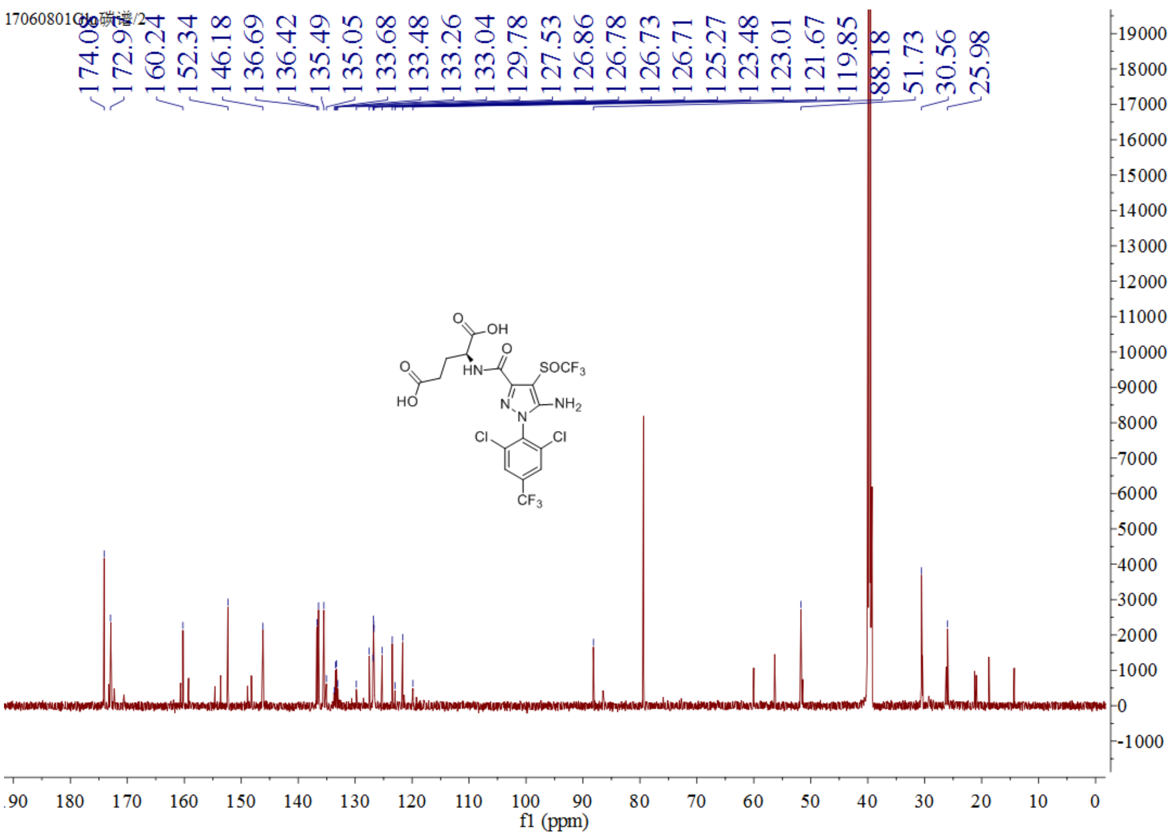

Figure S50 ^{13}C NMR spectra of **4l** in DMSO-d_6

Table S1. The fatality rates of **4a-l** and fipronil against *Plutella xylostella*

**Compounds** [a]	**12 h Fatality rate** (%)	**24 h Fatality rate** (%)
**4a**	15.15	48.48
**4b**	32.26	80.65
**4c**	3.33	66.67
**4d**	6.06	84.85
**4e**	9.09	78.79
**4f**	8.11	40.54
**4g**	3.33	55.67
**4h**	0.00	32.26
**4i**	8.57	68.57
**4j**	0.00	12.50
**4k**	15.63	81.25
**4l**	46.67	86.67
Fipronil	100.00	100.00

[a] at the concentration of 100 mg/mL.
